# High-surface-area corundum nanoparticles by resistive hotspot-induced phase transformation

**DOI:** 10.1038/s41467-022-32622-4

**Published:** 2022-08-26

**Authors:** Bing Deng, Paul A. Advincula, Duy Xuan Luong, Jingan Zhou, Boyu Zhang, Zhe Wang, Emily A. McHugh, Jinhang Chen, Robert A. Carter, Carter Kittrell, Jun Lou, Yuji Zhao, Boris I. Yakobson, Yufeng Zhao, James M. Tour

**Affiliations:** 1 Department of Chemistry, Rice University, Houston, TX 77005 USA; 2 Department of Electrical and Computer Engineering, Rice University, Houston, TX 77005 USA; 3 Department of Materials Science and NanoEngineering, Rice University, Houston, TX 77005 USA; 4 Smalley-Curl Institute, Rice University, Houston, TX 77005 USA; 5 Corban University, 5000 Deer Park Drive SE, Salem, OR 97317 USA; 6 NanoCarbon Center and the Welch Institute for Advanced Materials, Rice University, Houston, TX 77005 USA; 7 Present Address: Department of Chemistry, Rice University, Houston, TX 77005 USA

**Keywords:** Inorganic chemistry, Electronic materials

## Abstract

High-surface-area α-Al_2_O_3_ nanoparticles are used in high-strength ceramics and stable catalyst supports. The production of α-Al_2_O_3_ by phase transformation from γ-Al_2_O_3_ is hampered by a high activation energy barrier, which usually requires extended high-temperature annealing (~1500 K, > 10 h) and suffers from aggregation. Here, we report the synthesis of dehydrated α-Al_2_O_3_ nanoparticles (phase purity ~100%, particle size ~23 nm, surface area ~65 m^2^ g^−1^) by a pulsed direct current Joule heating of γ-Al_2_O_3_. The phase transformation is completed at a reduced bulk temperature and duration (~573 K, < 1 s) via an intermediate δʹ-Al_2_O_3_ phase. Numerical simulations reveal the resistive hotspot-induced local heating in the pulsed current process enables the rapid transformation. Theoretical calculations show the topotactic transition (from γ- to δʹ- to α-Al_2_O_3_) is driven by their surface energy differences. The α-Al_2_O_3_ nanoparticles are sintered to nanograined ceramics with hardness superior to commercial alumina and approaching that of sapphire.

## Abstract (web summary)

High-surface-area corundum are used in ceramics and catalyst supports, yet the synthesis is hampered by high energy barrier and aggregation. Here the authors report the ultrafast synthesis of corundum nanoparticles via the resistive hotspot triggered phase transformation in electric heating process.

## Introduction

High-surface-area corundum nanoparticles (α-Al_2_O_3_ NPs) have widespread applications. For examples, corundum is widely used in ceramics for biomedical implants^1,2^ and high-speed cutting tools^3^. α-Al_2_O_3_ NPs precursors provide access to nanometer-grained alumina ceramics with significantly improved fracture toughness^4^, wear resistance^5^, and high density under reduced sintering temperature^6^. Even though γ-Al_2_O_3_ NPs are primarily used as catalyst supports due to their high surface areas^7^, the α-Al_2_O_3_ with high surface area could be used as a catalytic support in auto-exhaust Pt-Mo-Co catalytic converters^8^ and enhance Ru catalyst activity for ammonia synthesis^9^. The high mechanical stability of α-Al_2_O_3_ enables a low sintering behavior, which is vital for its use in reforming reactions to obtain synthesis gas under harsh conditions^10,11^.

Much effort has been made toward improving the synthesis of α-Al_2_O_3_, yet very few of the processes afford high-surface-area NPs due to various intrinsic thermodynamic limits^6,12,13^. First, even though corundum is the thermodynamically stable phase of coarsely crystalized aluminum oxide (Al_2_O_3_), the synthesis of nanocrystalline Al_2_O_3_ usually leads to γ-Al_2_O_3_ because of its lower surface energy based on previous experimental observation and theoretical calculation^12,14,15^. The second reason is the high activation energy barrier of ~485 kJ mol^–1^ for the phase transformation from the cubic close-packed structure of the γ-phase to the hexagonal close-packed structure of the α-phase that involves intensive bond breaking and remaking^16^. Thirdly, the density of α-Al_2_O_3_ (3.99 g cm^–^^3^)^17^ is higher than that of the transition alumina phases (3.6–3.67 g cm^–3^ for γ-, η-, and δ-Al_2_O_3_)^17^, thus sufficient energy or high pressure^18^ is needed for the density uphill process from transition alumina to α-Al_2_O_3_. As a result, the thermal processes usually require temperatures >1470 K with prolonged annealing times of 10 to 20 h to facilitate the phase transformation^16,17^, which could also result in detrimental aggregation and sintering of alumina phases. The high-energy input and extended high-temperature annealing usually lead to surface area <10 m^2^ g^–1^ because of the substantial mass transfer^13^. Moreover, the polymorphism of Al_2_O_3_ during the phase transformation further increases the complexity and could lead to the mixed transition alumina with undesired δ- and θ-Al_2_O_3_^16,19,20^.

The phase transformation and grain growth of alumina usually coexist in any thermal process. Previous kinetics studies show that the activation energies of the phase transformation from γ- to α-Al_2_O_3_ are around 200–500 kJ mol^–1^ depending on the sample conditions^16,21,22^; in contrast, the activation energies for the grain growth are around 500–900 kJ mol^–1^ depending on the grain-boundary orientations^23–26^. This implies that the kinetics of the phase transformation is possibly faster than the grain growth. Hence, based on these prior results, it is reasonable to presume that a rapid and lower-temperature thermal process would reduce the grain coarsening and maintain high surface area during the phase transformation process.

To meet these goals, here, we show a Joule heating process based on pulsed direct current (PDC) to complete the phase transformation from γ- to α-Al_2_O_3_ at a significantly reduced average bulk temperature and reaction duration (~573 K, < 1 s). The rapid transformation is enabled by the resistive hotspot-induced local heating in the PDC process when an appropriate volume fraction ratio of γ-Al_2_O_3_ precursors and carbon black conductive additives are used. The pulsed and local heating mitigates the aggregation, leading to the synthesis of α-Al_2_O_3_ NPs with an average particle size of ~23 nm and a surface area of ~65 m^2^ g^–1^. Ab initio calculations reveal that the topotactic phase transformation process (from γ- to δʹ- to α-Al_2_O_3_) is driven by the surface energy difference of the three phases. The calculations suggest that a particle size of ~17 nm is the thermodynamic limit for the synthesis of anhydrous α-Al_2_O_3_ NPs with the δʹ-Al_2_O_3_ as the intermediate phase by a thermal process, matching well with the experimental values. Also, based on the Joule heating technique, we develop an alternating current sintering (ACS) process and show the ultrafast and pressureless sintering of the α-Al_2_O_3_ NPs into alumina ceramics with nanoscale grain size. The ceramics from these α-Al_2_O_3_ NPs by two-step pressureless sintering process demonstrate hardness of ~15 GPa, superior to commercial standard alumina and comparable to single-crystal sapphire.

## Results

### Phase transformation synthesis of corundum nanoparticles by PDC

**Fig. 1:**
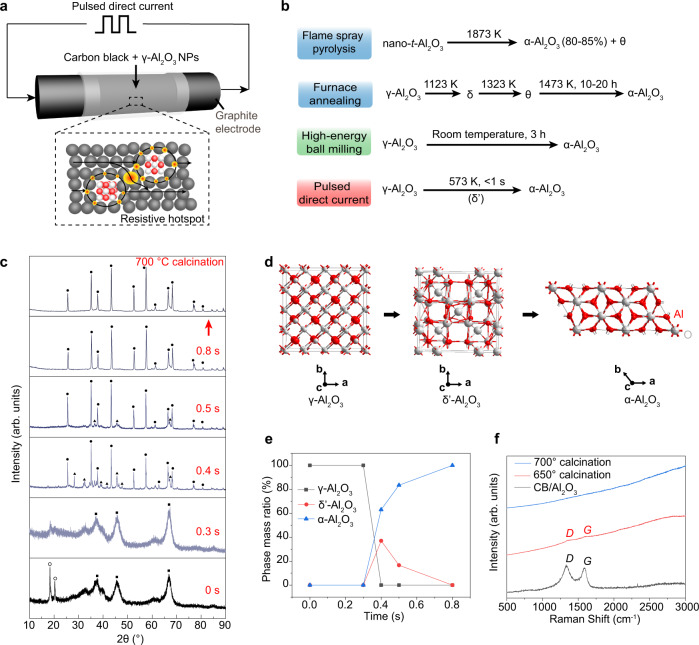
Ultrafast phase transformation of alumina by pulsed direct current (PDC) Joule heating. **a** Schematics of the PDC apparatus, and the resistive hotspots around and at the gaps of the insulative γ-Al_2_O_3_ NPs. The black arrows depict the electric current lines. **b** Representative methods for the phase transformation from γ- to α-Al_2_O_3_: flame spray pyrolysis, ref. 20 furnace annealing, ref. 16 high-energy ball milling, ref. 13 PDC, this work. **c** X-ray diffraction (XRD) patterns of γ-Al_2_O_3_ NPs after different PDC durations and the α-Al_2_O_3_ NPs product after calcination. The marks: γ-Al_2_O_3_ (square), δʹ-Al_2_O_3_ (triangle), α-Al_2_O_3_ (dot), and γ-Al(OH)_3_ (circle). The precursor is γ-Al_2_O_3_ with ~9 wt% γ-Al(OH)_3_ phase (gibbsite, crystal system: monoclinic; space group: P21/n; PDF No. 07-0324). The 0.8 s sample was calcined at 700 °C for 1 h. **d** Crystal structures of alumina phases: γ-Al_2_O_3_ (crystal system: cubic; space group: Fd-3m; PDF No. 10-0425), δʹ-Al_2_O_3_ (crystal system: orthorhombic; space group: P222; PDF No. 46−1215), and α-Al_2_O_3_ (crystal system: trigonal; space group: R-3c; PDF No. 46−1212). For γ-Al_2_O_3_, all the Al sites are depicted to show the crystal structure, while in the actual structure, not all the sites are 100% occupied. **e** Phase mass ratio of alumina polymorphs varied with PDC duration. **f** Raman spectra of as-synthesized α-Al_2_O_3_/carbon black mixture and the purified α-Al_2_O_3_ NPs by calcination at different temperatures.

Since the γ-Al_2_O_3_ NPs precursors are electrically insulative, commercial carbon black (CB) was used as the conductive additive. In a typical experiment, the mixture of γ-Al_2_O_3_ NPs and CB were compressed inside a quartz tube between two graphite electrodes (Fig. 1a, Supplementary Fig. 1a and Supplementary Table 1). The CB also works as separators to avoid the aggregation of Al_2_O_3_ NPs during heating. The carbon black is composed of ultrafine amorphous carbon nanospheres and has a surface area of ~1600 m^2^ g^–1^, which permits the intimate mixing with γ-Al_2_O_3_ precursors, as confirmed by the energy dispersive X-ray spectroscopy (EDS) maps (Supplementary Fig. 2). The resistance was controlled by the compressive force on the two electrodes (Supplementary Table 1). The electrodes were connected to a capacitor bank with total capacitance of *C* = 0.624 F and charging voltage up to *V*_0_ = 500 V. The discharge circuit is a series resistor-inductor-capacitor circuit with the characteristic time of *τ* = 0.1 ms, which permits the PDC with frequency of *f* = 1000 Hz (Supplementary Fig. 1b). Joule heating affects the entire electric conductor; for a homogeneous conductor, the current density is uniform so the Ohmic dissipation enables the homogeneous temperature distribution throughout the sample^27^. However, when an electrical field is applied to an inhomogeneous medium, as in the composite of conductive CB and insulative Al_2_O_3_, the current and powder densities have strong spatial variation^28^. The power dissipation is substantially larger at some regions than the neighbor; these regions are termed resistive hotspots (Fig. 1a). Even though the average bulk temperature is low, the hotspots permit local heating and trigger the transformation that happens at a much higher temperature.

By using this effect, we realized the phase transformation from γ-Al_2_O_3_ to α-Al_2_O_3_ accompanied by the intermediate phase of δʹ-Al_2_O_3_ at an average bulk temperature of ~573 K in <1 s (Fig. 1b, bottom). We compared our process to the representative phase transformation methods reported in the literatures (Fig. 1b)^13,16,20^. The liquid-feed flame spray pyrolysis produces α-Al_2_O_3_ at temperatures near 1873 K (Fig. 1b, top); however, the kinetically controlled process may render it difficult to access the pure phase (80–85% purity of α-phase)^20^. This phase purity is not an issue for ceramics since all other phases would transform to α-phase during the sintering process; nevertheless, the pure phase would be important for other applications such as in catalyst supports. Traditional heating methods that supply heat through the sample boundary, such as furnace annealing, require an extended period to permit uniform heating; hence 1473 K and 10 to 20 h are necessary to complete the phase conversion (Fig. 1b, middle)^16^. Other room-temperature nonequilibrium processes, such as high-energy ball milling, have been reported to form α-Al_2_O_3_ (Fig. 1b, middle)^13^ that shows hydrothermal stability^29^. Nevertheless, the Al_2_O_3_ can aggregate, which leads to loss of surface area during the extended time and high-energy collisions^30,31^.

We investigated the detailed phase transformation process of γ-Al_2_O_3_ by the PDC approach (Fig. 1c, d, e). Commercial γ-Al_2_O_3_ NPs with particle size of 5–10 nm and surface area of ~156 m^2^ g^–1^ were used as the precursors (Supplementary Figs. 3–5). X-ray diffraction (XRD) characterization and Rietveld refinement show that the starting materials are composed of ~91 wt% γ-Al_2_O_3_ with crystalline size of ~4 nm, and ~9 wt% γ-Al(OH)_3_ (Supplementary Fig. 6a). The γ-Al(OH)_3_ could be easily decomposed to γ-Al_2_O_3_ by mild calcination (Supplementary Fig. 6b). The mass ratio of γ-Al_2_O_3_ NPs and CB is 4 to 1, which gives a sample resistance of ~8 Ω (Supplementary Table 1). A discharging voltage of 60 V was applied with different discharging times controlled by a relay. The XRD patterns of the products with different PDC on-state time are shown in Fig. 1c. As the discharging time increased, the γ-Al(OH)_3_ first disappeared at 0.3 s; then, the γ-Al_2_O_3_ was transferred to the mixed δʹ- and α-Al_2_O_3_ at 0.4 to 0.5 s; last, the intermediate δʹ-Al_2_O_3_ was fully converted to α-Al_2_O_3_ after 0.8 s of discharge (Fig. 1e). The orthorhombic δʹ-Al_2_O_3_ is observed as the single intermediate phase (Fig. 1d), which is distinct from other thermal processes where δ- and θ-Al_2_O_3_ usually appear before the final α-Al_2_O_3_ phase (Fig. 1b)^16,17,32^.

Unlike our previous report^33^ on the synthesis of graphene by the high-voltage flash Joule heating at a high temperature of ~3000 K, the 60 V PDC does not provide enough energy to graphitize the CB (Supplementary Fig. 7). As a result, the residual CB (~20 wt%) could be easily removed by heating in air, according to thermal gravimetric analysis (TGA) (Supplementary Fig. 8a). Here, the as-synthesized mixture of α-Al_2_O_3_ NPs and CB was calcined in air at 700 °C for 1 h to purify the product. The X-ray photoemission spectrum (XPS) of the α-Al_2_O_3_ product after calcination showed very minor carbon signal, which could be caused by the carbon adsorption in air (Supplementary Fig. 8b). Raman spectra are sensitive to even a monolayer of carbon^34^; intriguingly, no characteristic Raman bands of carbon were detected after calcination at 700 °C (Fig. 1f), demonstrating the efficient removal of carbon. As a control, we show that the calcination process itself does not trigger the phase transformation and has negligible effect on the coarsening or aggregation of the γ-Al_2_O_3_ phase (Supplementary Fig. 9).

### Characterization of the corundum nanoparticles

**Fig. 2:**
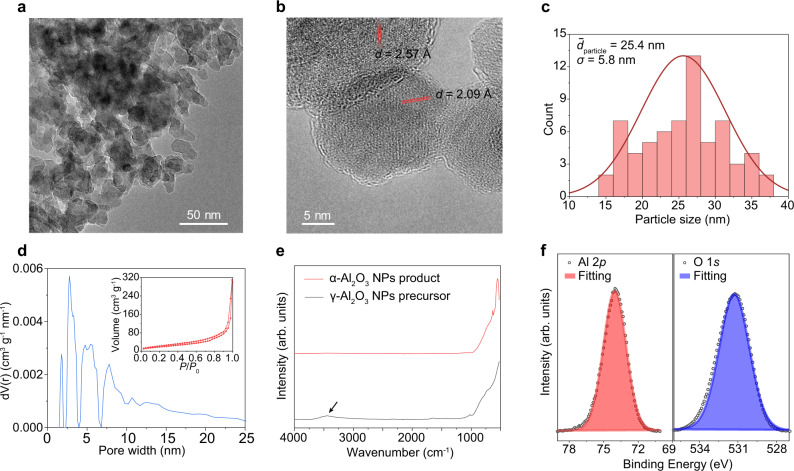
Characterization of the α-Al_2_O_3_ NPs. **a** Bright-field transmission electron microscopy (BF-TEM) image of the α-Al_2_O_3_ NPs. **b** High-resolution TEM (HRTEM) image of the α-Al_2_O_3_ NPs. The *d* spacing of 2.57 Å and 2.09 Å correspond to the *d*($10\bar{1}4$) and *d*($11\bar{2}3$) of α-Al_2_O_3_. **c** Histogram and distribution of the α-Al_2_O_3_ NPs particle size determined by TEM. **d** Pore width distribution determined by the application of density functional theory (DFT) model to the N_2_ isotherm. Inset, N_2_ adsorption–desorption isotherm of as-synthesized α-Al_2_O_3_ NPs at 77 K. **e** Fourier-transform infrared (FT-IR) spectra of the γ-Al_2_O_3_ NPs precursor and the α-Al_2_O_3_ NPs product. The black arrow points to the hydroxyl group absorbance. **f** X-ray photoelectron spectroscopy (XPS) fine spectra of Al and O of the α-Al_2_O_3_ NPs.

The α-Al_2_O_3_ NPs derived by PDC followed by mild calcination were further characterized in detail. Bright-field transmission electron microscopy (BF-TEM) images showed the well-dispersed particles (Fig. 2a). High-resolution TEM (HRTEM) showed the high degree of crystallinity of the α-Al_2_O_3_ NPs (Fig. 2b). The interplanar spacing values of ~2.57 Å and ~2.09 Å correspond to the *d*($10\bar{1}4$) and *d*($11\bar{2}3$) of α-Al_2_O_3_, respectively. The single set of lattice fringes in the NP and the nanobeam diffraction (NBD) show that the as-synthesized α-Al_2_O_3_ NPs are single-crystal particles (Supplementary Fig. 10). We observed some α-Al_2_O_3_ NPs with surface roughness features at a few nm (Supplementary Fig. 10), which are similar to the particle size of the γ-Al_2_O_3_ precursors (Supplementary Fig. 4). This demonstrates that the rapid PDC process triggers the phase transformation while no significant aggregation of the NPs occurs. The TEM images show that the particle size ranged from 14 to 36 nm, with an average particle size of 25.4 nm and standard derivation (*σ*) of 5.8 nm (Fig. 2c).

Brunauer–Emmett–Teller (BET) measurement showed that the surface area of the α-Al_2_O_3_ NPs is ~65 m^2^ g^–1^ (Fig. 2d, inset). The average particle size (*D*) is estimated to be ~23 nm by Eq. (1),

1 $$D=\,6/(\rho S)$$

where *ρ* is the density of α-Al_2_O_3_ (3.96 g cm^–3^) and *S* is the specific surface area^35^. The pore size determined from the N_2_ adsorption–desorption isotherm using the density functional theory (DFT) model^36^ indicates the distribution with high probability at 3 to 10 nm (Fig. 2d and Supplementary Fig. 11). The observed surface area was mainly attributed to the nanoscale grain size or intraparticle pores, and minor interparticle voids and surface roughness features of the NPs (Supplementary Fig. 10). The crystalline size of the α-Al_2_O_3_ NPs was estimated to be ~22 nm based on the Halder-Wagner method (Supplementary Note 1 and Supplementary Fig. 12). The crystalline size (~22 nm) agrees well with the particle size measured from TEM statistics (~25 nm) and BET estimation (~23 nm), demonstrating the single-crystal feature of the NPs. The dynamic light scattering (DLS) measurement shows that the as-synthesized α-Al_2_O_3_ NPs are well-dispersible (Supplementary Fig. 13).

Unlike the starting γ-Al_2_O_3_ NPs that have hydrated surface states, the as-synthesized α-Al_2_O_3_ NPs surfaces are highly dehydrated because of the thermal process (Fig. 2e). The XPS fine spectra showed the dominate O^2-^ peak at a binding energy of ~531.2 eV and single Al^3+^ peak at a binding energy of ~74.0 eV from the α-Al_2_O_3_ NPs (Fig. 2f). This demonstrated that the ultrafast PDC process does not result in obvious oxygen deficiencies or the carbothermic reduction of Al_2_O_3_ even with the existence of CB, presumably due to the high reduction potential of Al^3+^. No other peak was detected in the XPS full spectrum (Supplementary Fig. 8b), indicating the high-purity synthesis ability of the electric thermal process. This makes it superior to the solvent-based methods including ball milling^13^ or co-precipitation^6^, which inevitably suffer from lengthy purification processes and chemical contaminants.

### Resistive hotspot effect revealed by current density simulation

**Fig. 3:**
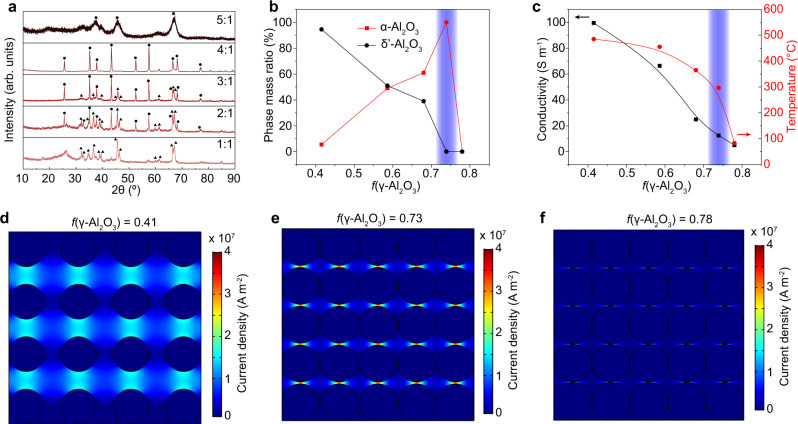
Resistive hotspot effect in pulsed direct current (PDC) process. **a** X-ray diffraction (XRD) patterns of γ-Al_2_O_3_/CB with different mass ratios after the same PDC process. The marks are γ-Al_2_O_3_ (square), δʹ-Al_2_O_3_ (triangle), and α-Al_2_O_3_ (dot). The numbers are the mass ratio of γ-Al_2_O_3_ to carbon black (CB). **b** Phase mass ratios of the product after PDC process varied with volume fraction of γ-Al_2_O_3_, *f*(γ-Al_2_O_3_). The blue region denotes the pure α-phase at *f* = 0.73. **c** Conductivities and temperatures varied with *f*(γ-Al_2_O_3_). The blue region denotes the pure α-phase at *f* = 0.73. **d**–**f** Simulated current density maps of the sample during PDC with different γ-Al_2_O_3_ volume fractions of **d** *f* = 0.41, **e** *f* = 0.73, and **f** *f* = 0.78. The separated balls are γ-Al_2_O_3_ NPs and the continuous phase is CB. The color bars show the current density values.

As discussed before, the composition of an inhomogeneous media is critical for local power dissipation during the PDC process. To quantitatively investigate the effect of the composition on the phase transformation, a series of precursors with different mass ratio of γ-Al_2_O_3_ and CB were treated by PDC under the same voltage and time (Fig. 3a and Supplementary Table 1). According to the densities of γ-Al_2_O_3_ and CB, the volume fractions (*f*) of γ-Al_2_O_3_ are obtained (Supplementary Note 2 and Supplementary Table 2), and the phase mass ratios varied with *f*(γ-Al_2_O_3_) after the PDC process are calculated (Fig. 3b). The phase transformation degree is increased as the *f*(γ-Al_2_O_3_) increased from 0.41 to 0.73; the phase-pure α-Al_2_O_3_ is obtained at *f*(γ-Al_2_O_3_) ~0.73. Further increase in the *f*(γ-Al_2_O_3_) to ≥ 0.78 leads to no phase transformation.

To explain the *f*(γ-Al_2_O_3_)-dependent phase transformation, the electrical conductivity and temperature were measured. The conductivities are calculated based on the measured resistance (*R*) and the feature size of the samples (Supplementary Table 1 and Fig. 3c). The conductivity is inversely proportional to *f*(γ-Al_2_O_3_), which is reasonable since γ-Al_2_O_3_ is electrically insulative. The real-time temperature was measured using an infrared (IR) thermometer (Supplementary Fig. 14). The average bulk temperature is decreased with the increase of *f*(γ-Al_2_O_3_) (Fig. 3c). This could be explained by the power (*P*) equation of Joule heating by Eq. (2),

2 $$P=\frac{{V}^{2}}{R}={V}^{2}\sigma$$

where *V* is the voltage, and *σ* is the conductivity of the sample. Since the start voltages were fixed to *V*_0_ = 60 V, the power was proportional to the conductivity of the sample. Intriguingly, the phase-pure α-Al_2_O_3_ NPs were obtained at a low average bulk temperature of ~573 K with *f*(γ-Al_2_O_3_) ~0.73 (Fig. 3c).

Such a low temperature is not supposed to trigger the phase transformation from γ- to α-Al_2_O_3_ with a high activation energy of ~485 kJ mol^–1^ (ref. 16). The phase transformation temperatures from transition alumina to α-Al_2_O_3_ in other thermal processes are substantially higher (Fig. 1b), e.g., flame spray pyrolysis at 1873 K (ref. 20), furnace annealing at 1473 K (ref. 16), and the annealing process even with α-Fe_2_O_3_ seeds at 973 K (ref. 6). Moreover, the higher phase transformation degree at a lower temperature is counterintuitive (Fig. 3c). To explain the intriguing phenomenon, we conduct a numerical simulation based on the finite element method (FEM) on the current density distribution of the γ-Al_2_O_3_/CB composite during PDC process (see details in Supplementary Note 2, Supplementary Figs. 15–19, and Supplementary Tables 3–5). As shown in Fig. 3d–f, the current density is inhomogeneous in the composite of γ-Al_2_O_3_ and CB; the current densities at the regions of vertical gaps between γ-Al_2_O_3_ NPs are larger than the bulk regions. The gaps become narrower as the *f*(γ-Al_2_O_3_) increased, leading to substantially large current densities in those regions. Considering that the resistivity (*R*) of the conductive CB phase is constant, the heat (*Q*) per volume produced by PDC is proportional to the square of the current density (*j*) by Eq. (3),

3 $$Q\propto {j}^{2}R$$

The large thermal dissipation in the regions with high current densities leads to the hotspots near γ-Al_2_O_3_ NPs with much higher temperature than the bulk regions, which triggers the phase transformation.

Experimentally, *f*(γ-Al_2_O_3_) of ~0.73 is the optimized volume fraction for the rapid and thorough phase transformation. Since the phase transformation temperature from γ- to α-phase using a furnace annealing is reported to be ~1473 K (ref. 16), we here defined the region as the hotspot zone with temperature *T*_hotspot_ ≥ 1473 K. Under such a definition, the hotspot zone is depicted (Supplementary Fig. 18), and it is estimated that ~30% of the particle surface area is heated to above the phase transformation temperature (see details in Supplementary Note 2, Estimation of the temperature and zone size of the hotspot). The quantitative analysis of the current densities suggests a decreased bulk temperature but an increased hotspot temperature as the *f*(γ-Al_2_O_3_) increases (Supplementary Fig. 19), which agrees well with the average bulk temperature measurement (Fig. 3c). Thus, the resistive hotspot-induced local heating during the PDC process well explains the observed phase transformation at a low bulk average temperature.

### Topotactic transition pathway revealed by ab initio calculations

**Fig. 4:**
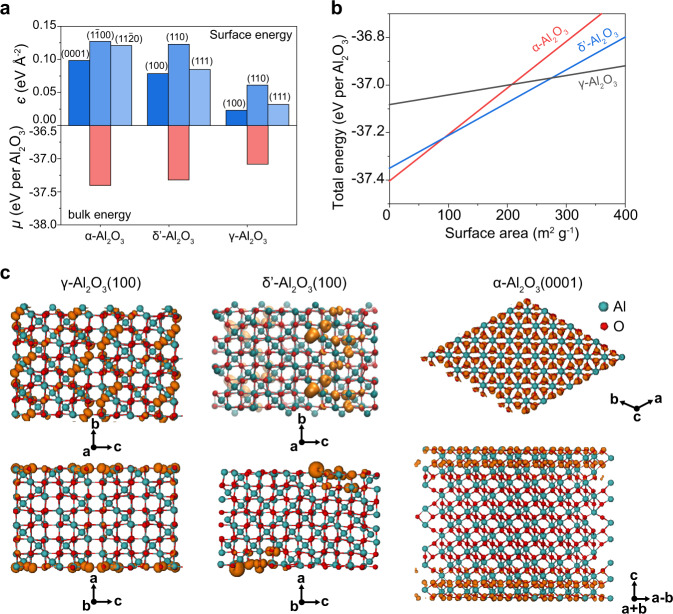
Topotactic phase transformation process revealed by density functional theory (DFT) calculations. **a** The bulk energies (*μ*, eV per Al_2_O_3_) and the surface energies (*є*, eV Å^–2^) of representative crystal surfaces for the three Al_2_O_3_ phases. The δʹ-Al_2_O_3_ and α-Al_2_O_3_ phases are anhydrous and the γ-Al_2_O_3_ phase is hydroxylated with 2 OH nm^–2^. **b** The total energy (the sum of bulk energy and surface energy) of the Al_2_O_3_ nanocrystals of three phases as plotted against the specific surface area. **c** The contour plots of partial charge density at the highest bands (0.3 eV below the Fermi levels) of the anhydrous surface states of γ-Al_2_O_3_(100), δʹ-Al_2_O_3_(100), and α-Al_2_O_3_(0001) from top view (top) and lateral view (bottom).

To provide deeper insight into the topotactic transition pathway, we conducted thermodynamic analysis of the three Al_2_O_3_ phases based on DFT (see details in Methods). Both the bulk energies and surface energies of the three Al_2_O_3_ phases were calculated (Fig. 4a and Supplementary Table 6). The bulk energy of α-Al_2_O_3_ is the lowest, followed by that of δʹ-Al_2_O_3_, and then γ-Al_2_O_3_, indicating that the α-Al_2_O_3_ is the most stable phase as a dense bulk crystal. The surface energies of (100), (110), and (111) facets of the cubic γ-Al_2_O_3_ and δʹ-Al_2_O_3_ were calculated, and the (0001), ($1\bar{1}00$), and $(11\bar{2}0)$ facets were calculated for hexagonal α-Al_2_O_3_ (Supplementary Fig. 20). The surface energy of Al_2_O_3_ is affected by the hydroxylated/anhydrous surface states^13,37^. The FT-IR spectra show that the intermediate δʹ-Al_2_O_3_ phase and the α-Al_2_O_3_ product are anhydrous (Fig. 2e and Supplementary Fig. 21a), while the γ-Al_2_O_3_ is hydroxylated. The TGA shows that the surface hydroxyl group density is ~2 OH nm^–2^ (Supplementary Fig. 21b); hence, the surfaces of γ-Al_2_O_3_ were modeled with this hydroxyl density (Supplementary Note 3 and Supplementary Figs. 22–23). It is found that the surface energy is opposite to the bulk energy, where γ-Al_2_O_3_ has the lowest surface energy, followed by δʹ-Al_2_O_3_, and then α-Al_2_O_3_ (Fig. 4a).

The nanocrystal shapes of the three phases were optimized by the Wulff theorem^38^, and the specific surface area and total energy were calculated (Supplementary Note 4 and Supplementary Fig. 24). The surface energy difference regulates the thermodynamic stability of the three Al_2_O_3_ phases (Fig. 4b), driving the phase transformation from γ-Al_2_O_3_ to δʹ-Al_2_O_3_ and then to α-Al_2_O_3_ phase as the surface area decreases, consistent with the experimental observation (Fig. 1b). When smaller than a surface area of ~93 m^2^ g^–1^, or larger than a particle size of ~17 nm, the α-Al_2_O_3_ phase becomes more stable than the δʹ-phase. Hence, this particle size is suggested as the thermodynamic limit for the synthesis of anhydrous α-Al_2_O_3_ by a thermal process that involves an intermediate δʹ-phase. The particle size of α-Al_2_O_3_ (~23 nm) synthesized by PDC approaches the thermodynamically limited value, and smaller than those obtained by most other thermal processes (Supplementary Table 7). The ultrafast, pulsed, and low-temperature PDC process to a large extent avoids mass transfer and grain coarsening during the phase transformation process. After adding the entropic contribution, which is minor compared to enthalpy, the free energy vs. surface area is plotted which leads to the same conclusions (Supplementary Note 5 and Supplementary Fig. 25). We note that the energy diagram is merely dependent on the specific surface area and irrelevant to the pore features.

To gain insight into the structural origin of the phase-dependent bulk and surface energy, the partial charge density contour at the highest bands (0.3 eV below the Fermi levels) of the surface states of the three Al_2_O_3_ phases were plotted (Fig. 4c). All of the surface atoms on α-Al_2_O_3_(0001) are active, while the sites with missing Al atoms on the δʹ-Al_2_O_3_(100) and γ-Al_2_O_3_(100) surfaces are relatively active (Fig. 4c, top). Closer analysis indicates that the active states go deep into the bulk for the δʹ-Al_2_O_3_(100) and γ-Al_2_O_3_(100) but not for α-Al_2_O_3_(0001) (Fig. 4c, bottom). This explains the bulk as well as surface energy sequences of the three Al_2_O_3_ phases, and identifies the Al vacancies in γ- and δʹ-phases as the structural origin of their thermodynamic stability/instability vs. the α-phase. The calculation explicitly shows that the phase transformation is thermodynamically driven by the surface energy differences among the three Al_2_O_3_ phases.

To further verify this, we conducted ab initio dynamic simulations of phase transformation between γ- and α-phases (Supplementary Note 6 and Supplementary Table 8). Owing to the limitation of current calculation capability, we were unable to depict the full diagram of the phase transformation details. Nevertheless, we found that when the particle size is smaller, the high surface energy of α-phase drives its transformation to a structure with typical local order features of γ-phase (Supplementary Figs. 26 and 27). In contrast, based on the bulk crystal model, the high bulk energy of γ-phase drives its transformation to a structure with higher coordination numbers of Al and O, approaching those of the α-phase (Supplementary Fig. 28 and Supplementary Table 9). Thus, the phase transition from α- to γ-phase is a surface-initiated process while the transition from γ- to α-phase is a bulk defect-initiated process, consistent with the calculated energy landscape (Fig. 4a-b) and the electronic structure analysis (Fig. 4c).

### Sintering of nanograined alumina ceramics

One prominent application of α-Al_2_O_3_ NPs is as the precursor for sintering high-strength nanograined alumina ceramics. The typical alumina ceramics sintering processes occur under high-pressure and high-temperature (HP-HT) conditions, such as hot isostatic pressing^39^, spark plasma sintering^18^, and pulse electric current sintering^40^. The high pressure, usually several GPa, retains the grain growth and advances densification^41^, which is critical for dense ceramic sintering using coarse grained precursors. The use of additives such as MgO can retard grain growth and advance densification^42,43^. However, the HP-HT process is not suitable for complex structures. The nanocrystalline precursors could undergo the pressureless sintering yet it would suffer from an elevated sintering temperature and prolonged time (>10 h)^6,44,45^. Spark plasma sintering that enables a heating rate up to 600 °C min^–1^ has been used for alumina sintering^46^. Very recently, an ultrafast high-temperature sinter method^47^ with heating rate up to 10^4^ °C min^–1^ based on direct current heating is reported for the rapid screening of ceramics.

**Fig. 5:**
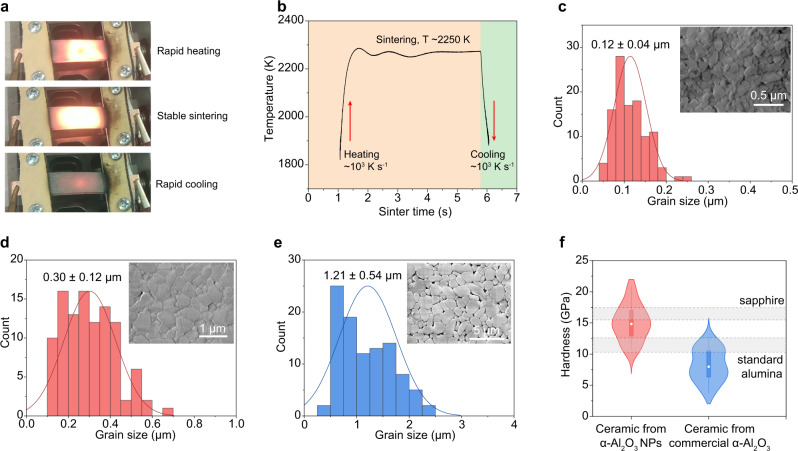
Sintering of the nanograined alumina ceramics. **a** Picture of the carbon papers during heating, sintering, and cooling. **b** Real-time temperature measurement during the alternating current sintering (ACS) process. **c** Grain size distribution of the alumina ceramic by ACS sintering using the α-Al_2_O_3_ NPs as precursor. Inset, scanning electron microscopy (SEM) image of the ceramic. **d** Grain size distribution of the alumina ceramic by two-step pressureless sintering (TS-PS) using the α-Al_2_O_3_ NPs as precursor. Inset, SEM image of the ceramic. **e** Grain size distribution of the alumina ceramic by TS-PS using the commercial α-Al_2_O_3_ nanopowders as precursor. Inset, SEM image of the ceramic. **f** Hardness distribution of the alumina ceramics by TS-PS process using the α-Al_2_O_3_ NPs (red) and commercial α-Al_2_O_3_ nanopowders (blue) as precursors. The dot within the box indicates the median, and the range indicates the 1.5IQR. The hardness of commercial standard alumina^46^ (10.5–12.7 GPa, lower band) and single-crystal sapphire^50^ (15.2–17.4 GPa, upper band) are labeled as reference.

Here, also based on the Joule heating technique, we developed the alternative current sintering (ACS) process for pressureless, ultrafast sintering of alumina ceramics. The ACS system can provide stable and high-energy output with voltages up to 63 V and currents up to 100 A (Supplementary Fig. 29a), making it suitable for the sintering of structural ceramics. Two separated, highly graphitized carbon papers connected to electrodes were used as the heating elements (Supplementary Fig. 29b). The α-Al_2_O_3_ NPs, mixed with polyethylene glycol (PEG) binder^48^, were pressed at 500 MPa into green body (See details in Methods). Commercial α-Al_2_O_3_ nanopowders (APS ~300 nm) were used as a control. After removal of the binder (5 °C min^–1^ to 500 °C for 2 h hold; in air), the green body was put between the carbon papers and under the ACS process at ~15 V (Fig. 5a). The temperature was recorded by fitting the blackbody radiation (Supplementary Fig. 30). The temperature rapidly ramped up to ~2250 K with a heating rate of ~10^3 ^K s^–1^. After stable sintering for 5 s, the sample cooled also with a rapid cooling rate of ~10^3 ^K s^–1^ (Fig. 5b).

Even after ACS sintering for only 1 min, the relative density of the ceramics from the α-Al_2_O_3_ NPs precursor reaches ~97%, higher than that from the commercial α-Al_2_O_3_ nanopowders at ~93% (Supplementary Fig. 31 and Supplementary Table 10). The microstructure of the alumina ceramics from α-Al_2_O_3_ NPs by scanning electron microscopy (SEM) showed the average grain size at ~0.12 μm (Fig. 5c); in comparison, the alumina ceramics sintered from the commercial α-Al_2_O_3_ nanopowders exhibited high residual porosity with grain size of ~1.15 μm (Supplementary Fig. 32), demonstrating that the sinter was in its initial stage. These results shows that the ultrafine particle size of the α-Al_2_O_3_ NPs facilitates the ultrafast sintering, presumably assisted by the grain growth at high temperature^6^. The mechanical properties were measured on the carefully polished ceramics (see details in Methods). Even with only 1 min ACS sintering, the Young’s modulus and hardness of the ceramics from the α-Al_2_O_3_ NPs reach ~86.0 GPa and ~6.2 GPa, respectively (Supplementary Fig. 33), higher than those from the commercial α-Al_2_O_3_ nanopowders (modulus ~40.6 GPa, hardness ~4.2 GPa).

To further improve the mechanical properties, the two-step pressureless sintering (TS-PS) using a high-temperature furnace was applied (see details in Method, Supplementary Fig. 34). Similar to the ACS process, the ceramics sintered by TS-PS from the α-Al_2_O_3_ NPs precursor have a higher density (~99%, Supplementary Fig. 31) and much finer grain size (~0.30 μm, Fig. 5d) than those from the commercial α-Al_2_O_3_ nanopowder precursors (density ~96% shown in Supplementary Fig. 31, and grain size ~1.21 μm shown in Fig. 5e and Supplementary Fig. 35). The mechanical properties of the ceramics were measured (Fig. 5f and Supplementary Fig. 35). The ceramics sintered from α-Al_2_O_3_ NPs precursor demonstrate an average Young’s modulus of ~179 GPa (Supplementary Fig. 36a), significantly higher than that from the commercial α-Al_2_O_3_ nanopowders (~106 GPa). The median hardness of the ceramics sintered from the α-Al_2_O_3_ NPs reaches ~15 GPa (Fig. 5f), which is better than the commercial standard alumina (10.5–12.7 GPa, ref. 49), and comparable to the single-crystal sapphire (15.2–17.4 GPa, ref. 50). We note that the mechanical properties of alumina ceramics are susceptible to various processing parameters^51,52^. For example, the hardness values of 15 – 20 GPa were achieved by Krell and coworkers^50,52^, who applied delicate specimens fabrication by hot isostatic pressing, pressure filtration, gel casting, etc. While we do not seek to systematically optimize the sintering process here, the density, grain size, and hardness obtained from the as-synthesized α-Al_2_O_3_ NPs are already superior to commercial products and comparable to most state-of-the-art reports (Supplementary Table 11).

## Discussion

To conclude, an ultrafast PDC processing of γ-Al_2_O_3_ was developed for the synthesis of fully dehydrated α-Al_2_O_3_ NPs (~23 nm) at significantly reduced temperature and duration (~573 K, < 1 s) than previous thermal processes. Numerical simulations reveal that the phase transformation was enabled by the resistive hotspot effect that induces local heating in the PDC process. Being a highly efficient energy supplies technology, Joule heating has a coefficient of performance of 1.0. The localized heating by resistive hotspots in PDC makes the process more effective because most of the electrothermal energy is directly targeted to the phase transformation. The phase transformation synthesis of α-Al_2_O_3_ NPs is realized with a low-energy input of ~4.77 kJ g^–1^ or $0.027 kg^–1^ in electrical energy cost, which is at least 20× less energy consumptive than a normal furnace annealing process (Supplementary Note 7). Moreover, the PDC process could be scalable by adjusting sample cross-sectional area and the PDC voltage, as suggested by the theoretical analysis of the key parameters (Supplementary Note 8). We demonstrated the synthesis of α-Al_2_O_3_ NPs up to 1.4 g-scale per batch within a similar timescale by using a higher PDC voltage (Supplementary Fig. 37).

The PDC process combined with the resistive hotspot effect greatly reduces the required temperature for reactions that should be originally triggered at a high-energy input, serving as an alternative technique for cost-efficient synthesis. We envision that this would be a universal strategy in electrical heating processes whenever a composite (one conductive phase and one non-conductive phase, or two phases with different conductivities) is used. The ACS process with the ultrafast and energy-efficient features, as demonstrated by the sintering of the alumina ceramic within 1 min, could also be promising in the sintering of functional ceramics, porous ceramics, or for materials screening^47^.

## Methods

### Materials

Commercial γ-Al_2_O_3_ nanopowders (US Nano, 99.99%, hydrophilic, average particle size of 5 nm, made by high-temperature combustion method) were used as the precursors. Prior to use, the starting material is extensively characterized by XRD (Fig. 1c), Rietveld refinement (Supplementary Fig. 6a), FT-IR (Fig. 2e), SEM (Supplementary Fig. 3), TEM (Supplementary Fig. 4), and BET (Supplementary Fig. 5). The XRD refinement shows that the starting material is composed of ~91 wt% γ-Al_2_O_3_ and ~9 wt% γ-Al(OH)_3_ according to the quantitative analysis. The γ-Al(OH)_3_ could be easily decomposed to γ-Al_2_O_3_ by mild calcination (in air, 700 °C for 1 h; Supplementary Fig. 6b). CB (Carbot, BP-2000) was used as the conductive additive. Commercial α-Al_2_O_3_ nanopowders (US Nano, 99.9%, 300 nm) were used as comparison for alumina ceramics sintering.

### PDC system and alumina phase transformation process

The electrical diagram of the PDC system is shown in Supplementary Fig. 1a. The γ-Al_2_O_3_ NPs precursor and CB with specific mass ratios were mixed by planetary ball milling (MSE Supplies, PMV1-0.4 L) for 2 h. The precursors (~150 mg per batch) were loaded into a quartz tube with an inner diameter (I.D.) of 8 mm and outside diameter (O.D.) of 12 mm. Graphite rods were used as the electrodes in both ends of the quartz tube. The use of graphite electrodes prevents the contamination of the product. The tube was then loaded on the reaction stage and connected to the PDC system. The resistance was controlled by the compressive force on the electrodes across the sample. The reaction stage was loaded into a plastic vacuum desiccator chamber under a mild vacuum (~10 mm Hg). A capacitor bank with a total capacitance of 0.624 F was charged by a DC supply that can reach voltages up to 500 V. A relay with programmable ms-level delay time was used to control the discharge time. A variable-frequency drive (VFD) was used to generate pulsed voltage with a frequency that ranges from 0 to 1000 Hz. In the synthesis, a voltage frequency of *f* = 1000 Hz was used. To prevent overheating, the discharge period (or ON state) was set to 20%, and the rest period (or OFF state) was set to 80% (Supplementary Fig. 1b). The heating time was calculated according to the discharge period. Detailed conditions are listed in Supplementary Table 1. After the PDC Joule heating, the apparatus was cooled to room temperature. CAUTION: There is a risk of electrical shock if improperly operated. Safety guidelines can be found in the Supplemental. After PDC Joule heating synthesis, the mixture of Al_2_O_3_ NPs and CB residues were calcined at 700 °C for 1 h in air using a Mafu furnace (NEY, MODELS 6-525) to remove the CB and purify the α-Al_2_O_3_ NPs product.

### Alumina ceramics sintering

#### Green body preparation

PEG was used as the binder^48^. PEG (M.W. 10000) was dissolved into deionized (DI) water at a concentration of 1 wt%. The α-Al_2_O_3_ NPs (~20 mg) and PEG were mixed with the PEG ratio of 3 wt%. After drying at 80 °C for 3 h in air, the α-Al_2_O_3_ NPs were pressed into pellets (diameter of 5 mm, thickness of ~0.5 mm) using a hydraulic press (500 MPa, dwell time of 10 min, Strongway Benchtop 10-Ton Hydraulic Shop Press). The binder was removed by calcining at 500 °C for 3 h in air at a heating rate of 5 °C min^–1^ using a Mafu furnace (NEY, MODELS 6-525). The commercial α-Al_2_O_3_ nanopoweders (~300 nm) were used as control.

#### ACS system and ACS sintering process

The electric diagram of the ACS system is show in Supplementary Fig. 29a. The total capacitance of the capacitor bank is 1.5 F. The system was capable of charging to voltage of 0 to 63 V and the current of 0 to 100 A. Two carbon papers (Toray Carbon Paper 060, FuelCellStore) attached to a glass slide were used as the heating element and sample holder (Supplementary Fig. 29b). The resistance of the carbon paper was ~1 Ω. The α-Al_2_O_3_ green bodies were put in between the carbon papers, which were connected to the ACS system. The voltage was set to ~15 V and the sintering time was 1 min.

#### Two-step pressureless sintering process

The two-step pressureless sintering (TS-PS) of alumina ceramics was conducted using a Mafu furnace (Carbolite RHF 1500) with the maximum temperature of 1500 °C. In the first step, the sample temperature ramps to 1425 °C with the heating rate of 5 °C min^–1^ and is maintained at 1425 °C for 2 h. In the second step, the sample cools to 1350 °C and is maintained for 5 h. Then, the sample slowly cools to room temperature (Supplementary Fig. 34).

#### Density measurement

The densities of the samples, including the green bodies and the sintered ceramics, are measured by Archimedes’ method.

#### Ceramics surface polishing

Prior to the mechanical property measurement, the sintered ceramic samples were carefully ground and polished using a wafer polisher (MultiPrep^TM^ Precision Polishing System). Diamond lapping film with grit of 15, 9, 6, 3, 1, and 0.1 μm were sequentially used. After the polishing process, mirror-like surfaces were obtained for the ceramics sintered from the α-Al_2_O_3_ NPs precursors. In contrast, we cannot obtain mirror-like surface for the ceramics sintered from commercial α-Al_2_O_3_ nanopowders precursors with the same polishing process.

#### Mechanical properties measurement

The Young’s modulus and Vickers hardness of ceramics were measured using a Hysitron TI 980 TriboIndenter. A diamond tip was used as the indenter. 25 curves were measured on each kind of ceramic sample surfaces to account for the deviation caused by residual porosity and heterogeneities. The reduced Young’s moduli were measured, and the Young’s moduli were calculated by Eq. (4),

4 $$\frac{1}{{E}_{{{{{{\rm{r}}}}}}}}=\,\frac{1-{v}^{2}}{E}+\frac{1-{v}_{{{{{{\rm{i}}}}}}}^{2}}{{E}_{{{{{{\rm{i}}}}}}}}$$

where *E*_r_ is the reduce Young’s modules of the sample, *E* is the Young’s modules of the sample, *v* is the Poisson’s ratio of the sample (*v* = 0.22), *E*_i_ is the Young’s modules of the indenter (*E*_i_ = 1220 GPa), and *v*_i_ is the Poisson’s ratio of the indenter (*v*_i_ = 0.2).

#### Microstructure characterization

For the ceramics sintered by the TS-PS process, the polished samples were thermally etching at 1400 °C using a furnace (Carbolite RHF 1500) for 30 min for SEM imaging^20^. For the ceramics sintered by the ultrafast ACS process, the thermal etching process is not suitable since it may change the original microstructures; hence, these samples were cracked and the fracture surfaces were characterized by SEM^42^. The grain size distribution is obtained by measuring 100 grains using ImageJ.

### Characterization

SEM images were obtained using a FEI Helios NanoLab 660 DualBeam SEM system at voltage of 15 kV and beam current of 100 pA. The element maps by EDS were obtained on a FEI Quanta 400 ESEM FEG system with an EDS detector (Oxford Instrument). The Raman spectra were acquired using a Renishaw Raman microscope (laser wavelength of 532 nm, laser power of 5 mW, and lens of 50×). XRD was collected by using a Rigaku Smartlab II system configured with a Cu Kα radiation (*λ* = 1.5406 Å). The Rietveld refinement was conducted using the GSAS-II software^53^. Rwp values <5% were achieved to secure good convergence. XPS analyses were conducted using a PHI Quantera XPS system under a base pressure of 5 × 10^-9 ^Torr. Elemental spectra were collected using a step size of 0.5 eV with the pass energy of 26 eV. All of the XPS spectra were calibrated by using the standard C 1 *s* peak at 284.8 eV. TEM images, selected area electron diffraction (SAED), and NBD patterns were taken on a JEOL 2100 field emission gun transmission electron microscope under the voltage of 200 kV. BET measurements were carried out on a Quantachrome Autosorb-iQ3-MP/Kr BET Surface Analyzer by using N_2_ as the adsorption/desorption gas at 77 K. FT-IR spectra were obtained using a Nicolet FT-IR Infrared Microscope. TGA measurement for carbon black removal was conducted in air at a heating rate of 10 °C min^–1^ by using a Q-600 Simultaneous TGA/DSC from TA instruments. TGA measurement of the surface hydroxyl coverage on γ-Al_2_O_3_ was conducted in N_2_ at a heating rate of 10 °C min^–1^ by using a Mettler Toledo TGA/DSC 3+ system from METTLER TOLEDO. The calcination in air (700 °C for 1 h) to remove the carbon black for purification of α-Al_2_O_3_ NPs was conducted using a Mafu furnace (NEY 6-160A). The DLS measurement was performed using a NanoSight NS300 system. Prior to measurement, the samples were dispersed in DI water and ultrasonicated using a cup horn sonicator for 0.5 h.

### Temperature measurement

For the PDC induce phase transformation process, the temperature was measured using an IR thermometer (Micro-Epsilon) with temperature measurement range of 200 to 1500 °C. The thermometer is connected to LabView software by using a Multifunction I/O (NI USB-6009) for real-time temperature recording (Supplementary Fig. 14a). Prior to use, the temperature was calibrated on the thermometer.

For the ACS of alumina ceramics process, the temperature was measured by fitting the blackbody radiation of the sample using a homemade, time-resolved spectrometer (Supplementary Fig. 30). The light emitted from the sample during Joule heating was collected by a 16-channel photomultiplier tube (PMT) array, with the spectrum range of 640–1000 nm. The sampling rate was 10 kHz, allowing for the temporal resolution of ~100 μs. The emission spectra were then fitted to the blackbody radiation to obtain the temperature (*T*) using Eq. (5),

5 $${B}_{{{{{{\rm{\lambda }}}}}}}\left(\lambda,\,T\right)=\,\gamma \frac{2h{c}^{2}}{{\lambda }^{5}}\frac{1}{{e}^{{hc}/\lambda {k}_{{{{{{\rm{B}}}}}}}T}-1}$$

where *λ* is the wavelength, *γ* is a constant introduced for fitting, *h* is the Planck constant, *c* is the speed of light in vacuum, and *k*_B_ is the Boltzmann constant.

### DFT calculations

The DFT method^54^ was used as implemented in the Vienna ab initio Simulation Package (VASP)^55^. A plane wave expansion up to 500 eV is employed in combination with an all-electron-like projector augmented wave (PAW) potential^56^. Exchange-correlation is treated within the generalized gradient approximation (GGA) using the functional parameterized by Perdew, Burke, and Ernzerhof^57^.

#### Bulk energy calculation

We first calculated the three bulk crystals, α-Al_2_O_3_, δʹ-Al_2_O_3_, and γ-Al_2_O_3_. The α-Al_2_O_3_ is the ground-state structure with a hexagonal R-3c symmetry. Both δʹ-Al_2_O_3_ and γ-Al_2_O_3_ structures are derived from the spinel MgAl_2_O_4_ structure with the space group of Fd-3m and a cubic unit cell (*a* = 8.17Å) of 4-Al_4_O_8_ layers and 8 Mg atoms in between. The Al atoms are octahedrally coordinated and Mg atoms are tetrahedrally coordinated. By replacing Mg with Al atoms, the obtained spinel Al_3_O_4_ has 32 O atoms, 16 Al atoms at the octahedral sites, and 8 Al atoms at the tetrahedral sites. A 1 × 1 × 3 supercell (Al_72_O_96_) is constructed of 12-Al_4_O_8_ layers in *z* direction with 24-tetrahedral Al atoms between the layers. Removing 8 out of the total 72 Al atoms in the supercell yields the Al_2_O_3_ formula^58^. The δʹ-phase is formed by removing an octahedrally coordinated Al atom from each Al_4_O_8_ layers, with one out of every three layers being skipped. The γ-phase is formed by removing tetrahedrally coordinated Al atoms in such a way that every two of the three layers are skipped. Periodic boundary conditions are applied to the unit cell in all three dimensions, with the size of the unit cells or lattice constants being optimized. The Brillouin zone integrations are performed using Monkhorst-Pack type meshes^59^, with sufficient meshes of *k*-points chosen so that the energy and lattice constant are fully converged. All structures are fully relaxed when the maximum force on each atom is smaller than 0.01 eV Å^–1^. The results of calculated bulk properties of the three phases are summarized in Supplementary Table 6.

#### Surface energy calculation

We calculated the surface energies of both pristine and OH-adsorbed surfaces. For the OH-adsorbed surface, H-adsorption exists to balance the charge^37^. For the hexagonal α-Al_2_O_3_ phase, three surfaces of (0001), ($1\bar{1}00$), and $(11\bar{2}0)$ are studied and no surface reconstruction is found to further lower the surface energy (Supplementary Fig. 20a). For both δʹ-Al_2_O_3_ and γ-Al_2_O_3_ phases derived from the original cubic spinel structure, the (001), (110), and (111) surfaces are investigated (Supplementary Figs. 20b, c). These defect-rich surfaces are found to have lower-energy reconstructions with rearrangement of surface atoms. The surface slabs (15–23 Å thick) are constructed so that the two surfaces bounded by vacuum are in inversion symmetry. Low-energy structures of adsorption of OH^─^ (on the Al sites) and H^+^ (on O sites)^37,60^ are searched computationally based on the optimized symmetry and homogenous distribution. The optimized atomic structures of γ-Al_2_O_3_ surfaces with ~2 OH nm^–2^ are shown in Supplementary Fig. 23. The vacuum layers between the slabs are chosen to be 10 Å or thicker. The surface energy (*є*, in eV Å^–2^) is calculated by Eq. (6),

6 $$\epsilon=({E}_{{{{{{\rm{slab}}}}}}}-N\mu -h{E}_{{{{{{{\rm{H}}}}}}}_{2}{{{{{\rm{O}}}}}}})/(2S),$$

where ${E}_{{{{{{\rm{slab}}}}}}}$ is the total energy of the slab, *N* is the total number of Al_2_O_3_ units in the slab, *μ* is the bulk energy in eV per Al_2_O_3_, *h* is the number of water molecules adsorbed in the form of split OH^─^ and H^+^, ${E}_{{{{{{{\rm{H}}}}}}}_{2}{{{{{\rm{O}}}}}}}$ is the total energy of a water molecule, and *S* is the surface area of one side of the slab. The calculated surface energies of typical crystal surfaces for the three Al_2_O_3_ phases, both with and without surface OH group adsorption, are shown in Supplementary Table 6.

#### Nanocrystal shape optimization based on Wulff construction

Strict analysis of formation energy of nanocrystals is based on the generalized Wulff theorem that considers bulk energy, surface energy, edge energy, and vertex energy^61^. However, direct optimization of the Wulff energy is practically forbidden because in most cases it is not feasible to determine the detailed atomic structures of the reconstructed high-index surfaces, edges, vertices. Therefore, the more practical and typical method involves a limited rational search of hypothetical structures of nanocrystals that potentially have the lowest energy^62^. In this study, we optimize the polyhedral shape of nanoparticles based on the surfaces listed in Supplementary Table 6. Optimization of the shape of nanocrystals is based on the generalized Wulff theorem^61^,

7 $${E}_{{{{{{\rm{total}}}}}}}=\mu+\sum ({S}_{i}{\epsilon }_{i}+{l}_{j}{e}_{j}+{v}_{k})/N$$

where *E*_total_ is the total energy, *μ* is the bulk energy per formular unit (or atom), *N* is the total number of formular unit in the particle, *S*_*i*_ is the area of the *i*^th^ facet of the nanocrystal, ${\epsilon }_{i}$ is the surface energy area of the *i*^th^ facet of the nanocrystal, ${l}_{j}$ is the length of the *j*^th^ edge, ${e}_{j}$ is the edge energy, and ${v}_{k}$ is the energy of the *k*^th^ vertex. Then the Eq. (7) can be further simplified by considering only the most probable facets with relatively lower indexes because the higher-index facets normally have much higher energy or complicated reconstruction, which make them unlikely occur in nanocrystals. The detailed shape optimization processes of the three Al_2_O_3_ phases are shown in Supplementary Note 4.

#### Plotting total energy vs. specific surface area

The total energy (*E*_total_) of the nanocrystal is expressed as Eq. (8),

8 $${E}_{{{{{{\rm{total}}}}}}}=(N\mu+\mathop{\sum}\limits_{i}{S}_{i}{\epsilon }_{i})/N$$

where *N* is the total number of Al_2_O_3_ units, *μ* is the bulk energy in eV per Al_2_O_3_, *S*_*i*_ is the area of each facet of the nanocrystal, ${\epsilon }_{i}$ is the surface energy in eV Å^–2^, and *N* = *V* × *n*, where *n* is the density, and volume *V* and *S*_*i*_ are the functions of the structural parameters of the polyhedrons. The specific surface area is calculated by Eq. (9),

9 $${{{{{\rm{surface}}}}}}\; {{{{{\rm{area}}}}}}=\frac{\sum {S}_{i}}{N\left(2{A}_{{{{{{\rm{Al}}}}}}}+3{A}_{{{{{{\rm{O}}}}}}}\right)u}$$

where *S*_*i*_ is the area of each facet of the nanocrystal, *N* is the total number of Al_2_O_3_ units, *A*_Al_ is the atomic mass of Al (26.9815), *A*_O_ is the atomic mass of O (15.9994), and *u* is the atomic mass (1.6605 × 10^–24 ^g). The total energy and the specific surface area of the three Al_2_O_3_ phases are calculated based on the optimized nanocrystal shapes, and their detailed expressions are shown in Supplementary Note 4. The energy of the nanocrystals is thus plotted against surface area (Fig. 4b).

## Supplementary information

Supplementary Information

## Data Availability

The data supporting the findings of this study are available within the article and its Supplementary Information. The source data generated in this study have been deposited in the Zenodo database under 10.5281/zenodo.6850605. Other relevant data are available from the corresponding authors upon request. Source data are provided with this paper.

## Source data

Source Data
