# Supplementary material for: High-surface-area corundum nanoparticles by resistive hotspot-induced phase transformation
Source: Nat Commun. 2022 Aug 26;13:5027. doi: 10.1038/s41467-022-32622-4 (PMC9418197; doi:10.1038/s41467-022-32622-4)
Supplement: Supplementary file 1 — Supplementary Information [file 41467_2022_32622_MOESM1_ESM.pdf]

Supplementary Information

High-surface-area corundum nanoparticles by resistive hotspot-induced phase transformation

Bing Deng[1], Paul A. Advincula[1], Duy Xuan Luong[1], Jingan Zhou[2], Boyu Zhang[3], Zhe Wang[1], Emily A. McHugh[1], Jinhang Chen[1], Robert A. Carter[1], Carter Kittrell[1], Jun Lou[3], Yuji Zhao[2], Boris I. Yakobson[1,3,4], Yufeng Zhao[5,*] & James M. Tour[1,3,4,6,*]

[1] Department of Chemistry, Rice University, Houston, Texas 77005, USA

[2] Department of Electrical and Computer Engineering, Rice University, Houston, Texas 77005, USA

[3] Department of Materials Science and NanoEngineering, Rice University, Houston, Texas 77005, USA

[4] Smalley-Curl Institute, Rice University, Houston, TX 77005, USA

[5] Corban University, 5000 Deer Park Drive SE, Salem, Orgon 97317, USA

[6] NanoCarbon Center and the Welch Institute for Advanced Materials, Rice University, Houston, Texas 77005, USA

*Email: Y.Z. (YZhao@corban.edu), J.M.T. (tour@rice.edu)

Supplementary Note 1. Crystalline size determination by Halder-Wagner's method.

The diffraction peak shows a visible spread if the crystalline size is smaller than 100 nm or if lattice strain is present. Hence, the diffraction peak spread is used to analyze crystalline size and lattice strain. Prior to analysis, the diffraction peak spread is corrected by the X-ray diffractometer. According to the Halder-Wagner's method in Supplementary Equation (1) (ref.[1]),

$$\frac{\beta^2}{\tan^2\theta} = \frac{K\lambda}{L}\frac{\beta}{\tan\theta\cdot\sin\theta} + 16e^2 \quad (1)$$

where θ is the diffraction angle, K is the Scherrer constant (being 1), L is the crystalline size, λ is the wavelength of wavelength of the X-ray (being 1.5406 Å here for Cu Kα), e is the lattice strain, and β is the integral width of corresponding diffraction peak and determined by Supplementary Equation (2),

$$\beta = \beta_{\mathrm{D}} + \beta_{\mathrm{S}} \quad (2)$$

where β_{S} is the integral width of a Lorentzian function which is described as the crystalline size, and β_{D} is the integral width of a Lorentzian function which is described as the lattice strain.

As shown in Supplementary Fig. 12, we plotted $\frac{\beta^2}{\tan^2\theta}$ against $\frac{\beta}{\tan\theta\cdot\sin\theta}$ and obtained the crystalline size L ~22 nm and lattice strain e = 0% of the samples.

Supplementary Note 2. The simulation of current density.

2.1 Volume fraction of γ-Al_2O_3 NPs.

The volume fractions (f) of the γ-Al_2O_3 were calculated based on the mass ratio and the densities of γ-Al_2O_3 and CB by Supplementary Equation (3),

$$f = \frac{M(\gamma-\mathrm{Al_2O_3})/\rho(\gamma-\mathrm{Al_2O_3})}{\frac{M(\gamma-\mathrm{Al_2O_3})}{\rho(\gamma-\mathrm{Al_2O_3})}+\frac{M(\mathrm{CB})}{\rho(\mathrm{CB})}} \quad (3)$$

The densities of the γ-Al_2O_3 and CB are measured to be ρ(γ-Al_2O_3) = 0.24 g cm^{-3} and ρ(CB) = 0.17 g cm^{-3}. The results were shown in Supplementary Table 2.

2.2 Simulation method.

The simulation was conducted based on the finite element method (FEM) by using the COMSOL Multiphysics 5.5 software. The Electric Currents interface in AC/DC module was used as the model. To simplify the simulation, we used a two-dimensional configuration. The geometric configuration and materials parameters were shown in Supplementary Table 3 and Supplementary Table 4, respectively. The electrical conductivity of CB was calculated by measuring the resistance since different commercial CB has different conductivity. Other materials parameters are from the physical constant table. The boundary conditions were shown in Supplementary Table 5. Supposing the linear drop of voltage throughout the sample, the electrical potential (V) could be calculated by Supplementary Equation (4),

$$V = V_0 L / L_0 \quad (4)$$

where V_0 is the overall potential (60 V), L_0 is the length of the sample (5 mm), and L is the geometrical size for the simulation. As a demonstration of f(γ-Al_2O_3) = 0.42, the γ-Al_2O_3 NPs were square packed, and the boundary conditions were shown in Supplementary Fig. 15a. The simulated electric potential map (Supplementary Fig. 15b) showed the linear decrease of electric potential from the Electric Potential boundary to Ground boundary. The simulated current density maps for various volume fraction of γ-Al_2O_3 are shown in Supplementary Fig. 16.

2.3 Estimation of the temperature and zone size of the hotspot.

We focused on the volume fraction of $f(\gamma\text{-}Al_2O_3) = 0.73$ (Fig. 3e), under which condition the phase transformation takes place most rapidly and thoroughly. The generated heat is determined by the Joule heating equation,

$$Q = I^2Rt \quad (5)$$

where Q is the heat amount, I is the current, R is the resistance, and t is the heating time.

On the other hand, the temperature change is determined by the following equation,

$$Q = cm\Delta T \quad (6)$$

where Q is the heat amount, c is the heat capacity of the sample, m is the sample mass, and ΔT is the temperature change. According to Supplementary Equations (5-6), we get the Supplementary Equation (7),

$$\Delta T = \frac{Rt}{cm} I^2 \quad (7)$$

Since c, m, R, and t are constant values for a specific sample, the temperature change is proportional to the square of current. For simplicity, we use the temperature changes and current per volume to revise Supplementary Equation (7), and we got the following equation,

$$\Delta T \propto j^2 \quad (8)$$

where j is the current density. The average bulk temperature could be experimentally measured (T_{bulk} = 573 K, Supplementary Fig. 14), which corresponds to the average bulk current density (j_{bulk}). The current density distribution contour map is obtained by numerical simulation (Supplementary Fig. 18a). We only consider the current passing through the carbon black since the Al_2O_3 phase is insulative and thus its current density is zero. Hence, from the contour map, the average bulk current density was calculated to be $j_{bulk} = 4.4 \times 10^6$ A m^{-2}.

As the name suggests, “hotspot” is a region wherein the temperature is substantially higher than the average bulk temperature. Hence defining a specific “hotspot zone size” is somewhat

blurred. Nevertheless, it is reasonable to define a threshold that the temperature is higher than the phase transformation temperature. Steiner et al. reported the phase transformation temperature from γ- to α-phase at T_{trans} = 1473 K (ref.[2]), which could be regarded as the threshold. Hence, here, we define the region with temperature $T_{hotspot} \geq 1473$ K as the hotspot. According to the above T_{bulk}, j_{bulk}, and $T_{hotspot}$ values, and Supplementary Equation (8), the threshold current density was calculated to be $j_{hotspot} \geq 9.1 \times 10^6$ A m^{-2}. Based on this value, we plotted the hotspot zone (Supplementary Fig. 18b). In the hotspot zone, the temperature is $T_{hotspot} \geq 1473$ K, and even higher at the center part of the hotspot zone. This high temperature triggers the ultrafast phase transformation.

According to Supplementary Fig. 18b, all particles were heated to above the phase transformation temperature at two vertical gaps regions. Furthermore, for a specific particle, ~29% of its surface area was heated to above the phase transformation temperature. This also suggests that phase transformation would take place at the surface and then penetrate into the bulk of the particle.

2.4 The effect of geometric configuration.

To demonstrate the effect of geometric configuration, we also simulated the hexagonal packing order using the same protocols (Supplementary Fig. 17). The current maps also show local maximums, or hotspots, in the vertical gaps between γ-Al_2O_3 NPs. These results show that the hotspot effect is just affected by the gap dimension of the γ-Al_2O_3 NPs and not related to the geometrical configuration. In the real sample, we used ball milling to mix γ-Al_2O_3 NPs and CB to ensure a best mixture. The real configuration of γ-Al_2O_3 NPs in CB matrix is three dimensional and random. Nevertheless, from the simulation results of the square stacking and hexagonal

stacking, it is reasonable to conclude that the proposed hotspot effect is available in the real three-dimensional system.

Supplementary Note 3. Consideration of surface OH group on γ-Al_2O_3.

The surface energy of the pristine and OH-adsorbing Al_2O_3 surfaces are plotted in Supplementary Fig. 20 with respect to OH coverage. Notice that in Supplementary Fig. 20, the surface energy decreases linearly with the increase of OH coverage. This allows for a fit of surface energies at 2 OH nm^{-2}, which are shown in Supplementary Table 6. Also, it is important to see that the surfaces energy of the γ-Al_2O_3 surfaces are significantly lower than α-Al_2O_3 and δ′-Al_2O_3. This is why at smaller sizes, the nanoparticles of γ-Al_2O_3 are more stable. Furthermore, the surface energy of γ-Al_2O_3 surfaces decrease much faster with OH adsorption, which explains the fact that we only observed OH adsorption on γ-Al_2O_3 NPs (Supplementary Fig. 19). In contrast, there is barely any OH on the α-Al_2O_3 and δ′-Al_2O_3 NPs.

The optimized atomic structures of γ-Al_2O_3 with surface OH groups density of ~2 OH nm^{-1} are shown in Supplementary Fig. 21.

Supplementary Note 4. Nanoparticle shape optimization by Wulff theorem.

4.1 Wulff energy for nanocrystals and surface energy of Al_2O_3.

Optimization of the shape of nanocrystals is based on the generalized Wulff theorem[3],

$$f = \mu + \sum (S_i \epsilon_i + l_j e_j + v_k) / N \quad (9)$$

where f is the total energy of the particle, μ is the bulk energy per formular unit (or atom), N is the

total number of formular unit in the particle, S_i is the area of the ith facet of the nanocrystal, ϵ_i is the surface energy of the ith facet of the nanocrystal, l_j is the length of the jth edge, e_j is the edge energy, and v_k is the energy of the kth vertex. The above equation cannot be rigorously treated if the shape is complicated with all possible facets considered. The equation can be further simplified by considering only the most probable facets with relatively lower indexes because the higher-index facets normally have much higher energy or complicated reconstruction, which make them unlikely to occur in nanocrystals. Therefore, in this study, we only consider three types of facets for the Al_2O_3 nanocrystals, equivalent to the surfaces listed in Supplementary Table 6, which are normally considered in this field[4].

4.2 Optimized shape of α-Al_2O_3 nanocrystals.

To optimize the shape of the α-Al_2O_3 nanocrystals with facets equivalent to (0001), $(11\bar{2}0)$, and $(1\bar{1}00)$, we minimized the Wulff energy expressed in Supplementary Equation (5). Considering that $(11\bar{2}0)$ and $(1\bar{1}00)$ surfaces form 150º angle, and they are orthogonal to (0001) surface, these surfaces naturally form a dodecagonal prism with bottom facets being (0001) surfaces and the side facets being $(11\bar{2}0)$ and $(1\bar{1}00)$ surfaces arranged alternatively side by side (Supplementary Fig. 24a). Because the surface energies of $(11\bar{2}0)$ and $(1\bar{1}00)$ are very close, we treated them equally in optimization. Assuming that the radius of circumcircle of the bottom dodecagon is *x* and the height of the dodecagonal prism is *a*, the volume of the prism *V*, the area of bottom facets S_0, and the area of the side facets S_1 can be expressed as,

$$V = 3x^2a, \quad (10)$$

$$S_0 = 6x^2, \quad (11)$$

$$S_1 = 24axsin(\frac{\pi}{12}) = \frac{8V}{x}sin(\frac{\pi}{12}). \quad (12)$$

The reduced Wulff energy (the bulk energy is a constant) is expressed as,

$$f_{(x)} = S_0\epsilon_0 + S_1\epsilon_1 = 6\epsilon_0 x^2 + \frac{8V\epsilon_1}{x} sin(\frac{\pi}{12}), \quad (13)$$

where ϵ_0 is the surface energy of (0001), and $\epsilon_1$is the average energy of $(11\bar{2}0)$ and $(1\bar{1}00)$ surface. Therefore, the above function is minimized at,

$$x = \frac{2a\epsilon_1}{\epsilon_0} sin(\frac{\pi}{12}). \quad (14)$$

With this structural parameter, one can easily find the relationship between the specific surface area S_A of the nanocrystals and the Wulff energy of α-Al_2O_3 nanocrystals (f_α),

$$S_\mathrm{A} = \frac{S_0+S_1}{Vn(2A_\mathrm{Al}+3A_\mathrm{O})u}, \quad (15)$$

$$f_\alpha = \mu_\alpha + \frac{S_0\epsilon_0+S_1\epsilon_1}{N} = \mu_\alpha + \frac{S_0\epsilon_0+S_1\epsilon_1}{Vn}, \quad (16)$$

where the atomic mass $A_\mathrm{Al} = 26.9815$, $A_\mathrm{O} = 15.9994$, atomic unit of mass u = 1.6605 × 10^{-24} g. The bulk energy, surface energies, and density n for α-Al_2O_3 are given in Supplementary Table 6. According to Supplementary Equations (10-12), and (14), for a particular V, there is a unique S_A and f_α. Therefore, we can plot f_α as the function of S_A.

4.3 Optimized shape of γ-Al_2O_3 nanocrystals.

The energy of (100) surface of γ-Al_2O_3 is significantly lower than that of the (110) surface. Therefore, (100) facets will dominate in the formation of nanocrystals. According to our derivation, (110) facets can only coexist with (100) when its surface energy is lower than $\sqrt{2}$ of that of (100). This condition is not satisfied according to the values listed in Supplementary Table 6. That means that the (110) facets cannot occur. Since the (111) surface has a lower energy than the (110) but higher than (100), we consider truncated cubic shape of γ-Al_2O_3 nanocrystals with six square (100) facets and eight triangular (111) facets. Assuming that the edge of the cube is a

and a portion of x ($x \leq \frac{a}{2}$) along the edge is truncated off at each corner (Supplementary Fig. 24b), the volume V, the total area of the (100) facets S_0, and the total area of the (111) facets S_1, are expressed respectively as,

$$V = a^3 - \frac{4}{3}x^3, \tag{17}$$

$$S_0 = 6a^2 - 12x^2 = 6\left(V + \frac{4}{3}x^3\right)^{\frac{2}{3}} - 12x^2, \tag{18}$$

$$S_1 = 4\sqrt{3}x^2. \tag{19}$$

The reduced Wulff energy (the bulk energy is a constant) is

$$f_{(x)} = S_0\epsilon_0 + S_1\epsilon_1, \tag{20}$$

where ϵ_0 and ϵ_1 are the surfaces energy of (100) and (111), respectively. The above energy is minimized at,

$$x = (3\epsilon_0 - \sqrt{3}\epsilon_1)a/2\epsilon_0. \tag{21}$$

With this structural parameter, one can similarly find the relationship between the specific surface area S_{A} of the nanocrystals and the Wulff energy of γ-Al_2O_3 nanocrystals (f_γ),

$$S_{\mathrm{A}} = \frac{S_0 + S_1}{Vn(2A_{\mathrm{Al}} + 3A_{\mathrm{O}})u}, \tag{22}$$

$$f_\gamma = \mu_\gamma + \frac{S_0\epsilon_0 + S_1\epsilon_1}{N} = \mu_\gamma + \frac{S_0\epsilon_0 + S_1\epsilon_1}{Vn}. \tag{23}$$

The bulk energy μ_γ, surface energies of (100) and (111) facets, and density *n* for γ-Al_2O_3 are given in Supplementary Table 6. According to Supplementary Equations (17-19), and (21), for a particular *V*, there is a unique S_{A} and f_γ. Therefore, we can plot f_γ as the function of S_{A}.

4.4 Optimized shape of δ′-Al_2O_3 nanocrystals

We note that in the truncated cube case of γ-Al_2O_3 nanocrystals, the truncated portion of

the edge *x* must be less than half of the length of the edge a. When the energy of (111) surface is further lowered than (100) surface, the optimized $x = (3\epsilon_0 - \sqrt{3}\epsilon_1)a/2\epsilon_0$ can be larger than *a*/2. Then the shape of the nanocrystals transforms into truncated octahedron with six square (100) facets and eight hexagonal (111) facets. This is the case of δ′-Al_2O_3 nanocrystals. Assuming that the edge of the octahedron is *a* and a portion of *x* along the edge is truncated off at each corner (Supplementary Fig. 24c), the volume *V*, the total area of the (100) facets S_0, and the total area of the (111) facets S_1, are expressed respectively as,

$$V = \frac{\sqrt{2}(a^3 - 3xa^3)}{3}, \quad (24)$$

$$S_0 = 6x^2, \quad (25)$$

$$S_1 = 2\sqrt{3}(a^2 - 3x^2). \quad (26)$$

The reduced Wulff energy (the bulk energy is a constant) is expressed as,

$$f_{(x)} = S_0\epsilon_0 + S_1\epsilon_1, \quad (27)$$

where ϵ_0 and $\epsilon_1$are the surfaces energy of (100) and (111), respectively. When minimizing the reduced Wulff energy, one finds the structure parameter of the truncated octahedron at,

$$x = \frac{\sqrt{3}\epsilon_1 - \epsilon_0}{\sqrt{3}\epsilon_1}a. \quad (28)$$

Again, we obtain the specific surface area S_A of the nanocrystals and the Wulff energy of δ′-Al_2O_3 nanocrystals ($f_{\delta'}$),

$$S_\mathrm{A} = \frac{S_0 + S_1}{\mathrm{V}n(2A_{Al} + 3A_O)u}, \quad (29)$$

$$f_{\delta'} = \mu_{\delta'} + \frac{S_0\epsilon_0 + S_1\epsilon_1}{N} = \mu_{\delta'} + \frac{S_0\epsilon_0 + S_1\epsilon_1}{Vn}. \quad (30)$$

The bulk energy $\mu_{\delta'}$, surface energies of (100) and (111), and density *n* for δ′-Al_2O_3 are given in Supplementary Table 6. According to Supplementary Equations (24-26), and (30), for a particular *V*, there is a unique S_A and $f_{\delta'}$. Therefore, we can plot $f_{\delta'}$ as the function of S_A.

Supplementary Note 5. The entropic contribution.

For solid reactions, the entropy contribution to the free energy change is usually much smaller than the enthalpy contribution. Hence, in our calculation, we only considered the energy (including bulk energy and surface energy) of the three phases of Al_2O_3.

We here estimated the entropic contribution to the phase transformation. According to the NIST table[5], under standard conditions, the entropies of three Al_2O_3 phases are (we note that entropy of δ′-Al_2O_3 could not be found in literature; since the δ-Al_2O_3 and δ′-Al_2O_3 phases have similar crystal structures, we here used the entropy of δ-Al_2O_3 as an alternative,

S(γ-Al_2O_3) = 52.300 J mol^{-1} K^{-1},

S(δ-Al_2O_3) = 50.626 J mol^{-1} K^{-1},

S(α-Al_2O_3) = 50.950 J mol^{-1} K^{-1}.

These entropy values could be converted to those per Al_2O_3 unit,

S(γ-Al_2O_3) = 5.4204×10^{-4} eV K^{-1},

S(δ-Al_2O_3) = 5.2469×10^{-4} eV K^{-1},

S(α-Al_2O_3) = 5.2805×10^{-4} eV K^{-1}.

According to the calculation (Fig. 4a, Supplementary Table 6), the bulk energy (or enthalpy) of the three Al_2O_3 phases per Al_2O_3 unit are,

H(γ-Al_2O_3) = -37.08 eV,

H(δ′-Al_2O_3) = -37.35 eV,

H(α-Al_2O_3) = -37.40 eV.

Considering the phase transformation temperature at T = 573 K according to the experimental measurement (Supplementary Fig. 14), the entropic contributions of the three phases per Al_2O_3 unit are calculated to be,

-TS(γ-Al_2O_3) = -0.3106 eV,

-TS(δ-Al_2O_3) = -0.3006 eV,

-TS(α-Al_2O_3) = -0.3026 eV.

These values are about ~1% of the enthalpy values of the three phases, indicating that the entropy contribution is much less than the enthalpy contribution to the free energy.

According to the Gibbs free energy equation, $\Delta G = \Delta H - T\Delta S$, the free energy of the three phases could be calculated. We plot the free energy and specific surface area, as shown in Supplementary Fig. 25. The main conclusion remains the same when just consideration of enthalpy, that is, the surface energy difference between the three phases drives the phase transformation from γ-Al_2O_3 to δ′-Al_2O_3 and then to α-Al_2O_3 phase. The transformative surface area from δ′-Al_2O_3 to α-Al_2O_3 phases happens at ~96 m^2 g^{-1}, comparable to the value of ~93 m^2 g^{-1} that just considering the energy calculation.

Supplementary Note 6. Dynamic simulations of the structural transformation.

In this study, we conduct dynamic simulations of phase transition of nanocrystals at two critical sizes, i.e., α- to γ- phase transition at <2 nm and γ- to α- phase transition at >20 nm. The smaller size (α- to γ-phase) can be directly handled in DFT simulation. But direct MD simulation of the larger particles (γ- to α- phase) using the DFT method is not feasible. To this end, we performed MD modeling of bulk phase transition using the Langevin thermostat in the isobari-

isothermic NPT ensemble[6,7], which allows for transformation of symmetry and size of the supercell. Although the initial crystalline structure can be chosen, the final structures formed are normally amorphous due to the limited time scale of simulation (<100 ps) and, for the bulk phase transition, the incommensurate lattice at the limited size of the supercell. Therefore, alternatively, we here use the coordination number of Al and O atoms as a descriptor of the local order to characterize the structural transformation. Local symmetry may also feature the two different phases. As shown in Supplementary Table 8, the α-Al_2O_3 has hexagonal symmetry and higher coordination numbers than the cubic structure of γ-Al_2O_3. All the Al atoms are in octahedral coordination with coordination number of 6 in α-Al_2O_3, while 1/4 of the Al atoms in γ-Al_2O_3 are tetrahedrally coordinated with coordination number of 4. Moreover, due to the defective bulk structure of γ-Al_2O_3, 1/3 of its O atoms only have three nearest neighboring Al atoms; in contrast, all O atoms in α-Al_2O_3 have 4 coordinated Al atoms.

We first performed NVT dynamics simulation of a 1.2×1.5×1.8 nm^3 α-phase nanocrystal at 1800 K for 10 ps following a 2 ps preheating. The hexagonal structure is transformed into an amorphous structure in less than 5 ps (Supplementary Figs. 26a-b), demonstrating the structural instability of α-phase at very fine particle size. After 10 ps, the system is quenched to 100 K in 2 ps and followed by structure optimization (Supplementary Fig. 26c). On the surface of the particle, the rectangular local bonding network is recognized (circles in Supplementary Fig. 26d); when looking into the particle, a substantial amount of tetrahedral Al atoms can be seen (arrows in Supplementary Fig. 26e). Both are typical features of local order of the γ-phase.

We further performed dynamic simulations of a 1.8-nm slab of the α-phase with both sides being the $(11\bar{2}0)$ surface. The simulation time is set to 20 ps, while the temperature varies from 1700 to 2500 K. Supplementary Fig. 27a-c shows the structure transformation at 2000 K from 2

to 20 ps. The optimized final structures show that four surface layers of atoms were transformed (Supplementary Fig. 27c). Similar structural transformation takes places irreversibly at all temperatures, indicating that these surface structures are energetically more favorable than the original $(11\bar{2}0)$ surface of α-phase. Again, we can recognize the local rectangular order on the surface (circles in Supplementary Fig. 27d), and the tetrahedral bonding configuration of some Al atoms in the deeper layer (arrows in Supplementary Fig. 27e). Therefore, based on the dynamic simulations, we demonstrate the high surface energy of the α-phase drives the structural transformation, consistent with the energy diagrams (Fig. 4b).

When the nanocrystals are big enough, the bulk energy lowering dominates the energy landscape, so the γ-phase would transform into the α-phase. To verify this, we constructed a γ-phase bulk structure using a $1.6\times1.6\times2.4$ nm^3 supercell. Then, NPT dynamics simulations were performed at 2000 K for 30 ps. The initial γ-Al_2O_3 and the final amorphous structures are shown in Supplementary Fig. 28. It is observed that in 2 ps, the structure collapses and the rectangular supercell is substantially distorted. It is not feasible to form a α-Al_2O_3 crystal out of the γ-Al_2O_3 due to the incommensurate lattices when a limited size of the supercell is used. In every 5 ps of the unified MD simulation process, the obtained intermediate structure is quenched separately to room temperature within 2 ps and optimized so that the coordination number of the atoms can be evaluated, as shown in Supplementary Table 9. Clearly, the average coordination numbers of Al and O atoms increase along with the annealing time and approaches to the values of 6.0 and 4.0, which features the local order of α-Al_2O_3, demonstrating the bulk densification. Therefore, based on the dynamic simulation, we demonstrate the high bulk energy of γ-phase drives the structural transformation, consistent with the energy diagrams (Fig. 4b).

Supplementary Note 7. Electrical energy consumption for the phase transformation.

The energy consumption was calculated by Supplementary Equation (31),

$$E = \frac{(V_0^2 - V_1^2) \times C}{2 \times M} \qquad (31)$$

where E is the energy per gram (kJ g^{-1}), V_0 and V_1 are the start voltage and voltage after Joule heating, respectively, C is the capacitance (C = 0.624 F), and M is the mass of Al_2O_3 per batch.

In a typical experiment, V_0 = 60 V, V_1 = 42 V, and M = 0.12 g, the energy consumption was calculated to be,

$$E = 4.77 \text{ kJ g}^{-1} = 1.33 \text{ kWh kg}^{-1}.$$

Given that the industrial price of electric energy in Texas, USA is $0.02/kWh, the electrical energy cost of the synthesis of corundum nanoparticles would be P = 0.027 $ kg^{-1}.

We compared our process with the other thermal processes with regard to energy consumption for the phase transformation synthesis of corundum NPs. We considered a furnace annealing process (1473 K and 10 h)[2] by using a commercial Muffle furnace (KSL-1200X-UL, MTI) with mass loading of ~10 kg and power of 3 kW. The energy consumption is calculated to be ~108 kJ g^{-1}. Hence, the resistive hotspot enabled localized heating in PDC process is 20× less energy consumptive than a normal thermal process.

Supplementary Note 8. Strategy for scaling up.

The synthesis of corundum NPs involves three steps: (1) the mixing of γ-Al_2O_3 with conductive carbon black; (2) the PDC Joule heating process for the ultrafast phase transformation; and (3) calcination in air for removing the excess carbon. In this work, the mixing of γ-Al_2O_3 precursors and carbon black is realized by ball milling (see details in Method). The ball milling is a well-established technique and there are already many industry-scale ball millers. Hence, step #1 is not

an obstacle for scaling up. The mild calcination (see details in Method) is done in a furnace and thus is highly scalable. Here we mainly discuss how we scale up the PDC Joule heating process.

In the PDC process, the temperature is the key thermodynamical handle for the phase transformation of the γ-Al_2O_3. Hence, to maintain a constant temperature values and distribution is critical for the scaling up of the PDC process.

8.1 Parameters determining the current density.

We firstly conducted theoretical analysis of the key parameters of the PDC Joule heating determining the temperature. The heat (Q) per volume produced by PDC Joule heating is proportional to the square of the current density (j) and the resistivity (ρ_e) of the sample according to Supplementary Equation (32),

$$Q \propto j^2 \rho_{\mathrm{e}} \tag{32}$$

Since the resistivity of the sample is constant with the same f(γ-Al_2O_3), to maintain a constant temperature value, we needed to keep a constant current density value when scaling up the sample. The current (I) is determined by the voltage (V) and the resistance (R) of the sample by Supplementary Equation (33),

$$I = V/R \tag{33}$$

The current density is calculated by Supplementary Equation (34),

$$j = \frac{I}{S} = \frac{V}{RS} \tag{34}$$

where S is the cross-sectional area of the sample.

The resistance of the sample is determined by Supplementary Equation (35),

$$R = \frac{\rho_{\mathrm{e}} L}{S} \tag{35}$$

where ρ_e is the resistivity of the sample, and L is the length of the sample.

The sample mass (m) is calculated by Supplementary Equation (36),

$$m = \rho_m S L \tag{36}$$

where ρ_m is the density of the sample.

Above all, we get the expression of the current density by Supplementary Equation (37),

$$j = \frac{I}{S} = \frac{V \rho_m S}{\rho_e m} \tag{37}$$

Since ρ_m and ρ_e are constant for a specific f(γ-Al_2O_3), to maintain a constant current density when increasing the mass, we need to increase either the sample cross sectional area or the voltage proportionally. Practically, increasing the voltage is feasible. High voltage (>1 kV) or even ultrahigh voltage (hundreds of kV) technologies are well-established.

8.2 Scaling up to gram scale.

In this work, most of the synthesis are conducted by using a sample mass of m_0 ~200 mg, tube diameter of D_0 = 8 mm, and V_0 = 60 V. To scale up the synthesis, we used a tube diameter of D_1 = 16 mm (Supplementary Fig. 37). Hence, the cross-sectional area ratio will be $S_1/S_0 = (D_1/D_0)^2$ = 3.5. For the sample mass of $m_1 = 3.5 \times m_0$ = 700 mg, according to Supplementary Equation (37), to maintain a constant current density, $V_1 = V_0$ = 60 V. As shown in Supplementary Figs. 37a-b, we realized the phase transformation using a PDC voltage of 60 V.

For the sample mass of $m_2 = 7 \times m_0$ = 1.4 g, according to Supplementary Equation (37), to maintain a constant current density, $V_2 = 2V_0$ = 120 V. As shown in Supplementary Figs. 37c-d, we realized the phase transformation using a PDC voltage of 120 V. Those results provided a simple scaling rule and supported a conclusion of scalability of the PDC process for alumina phase transformation.

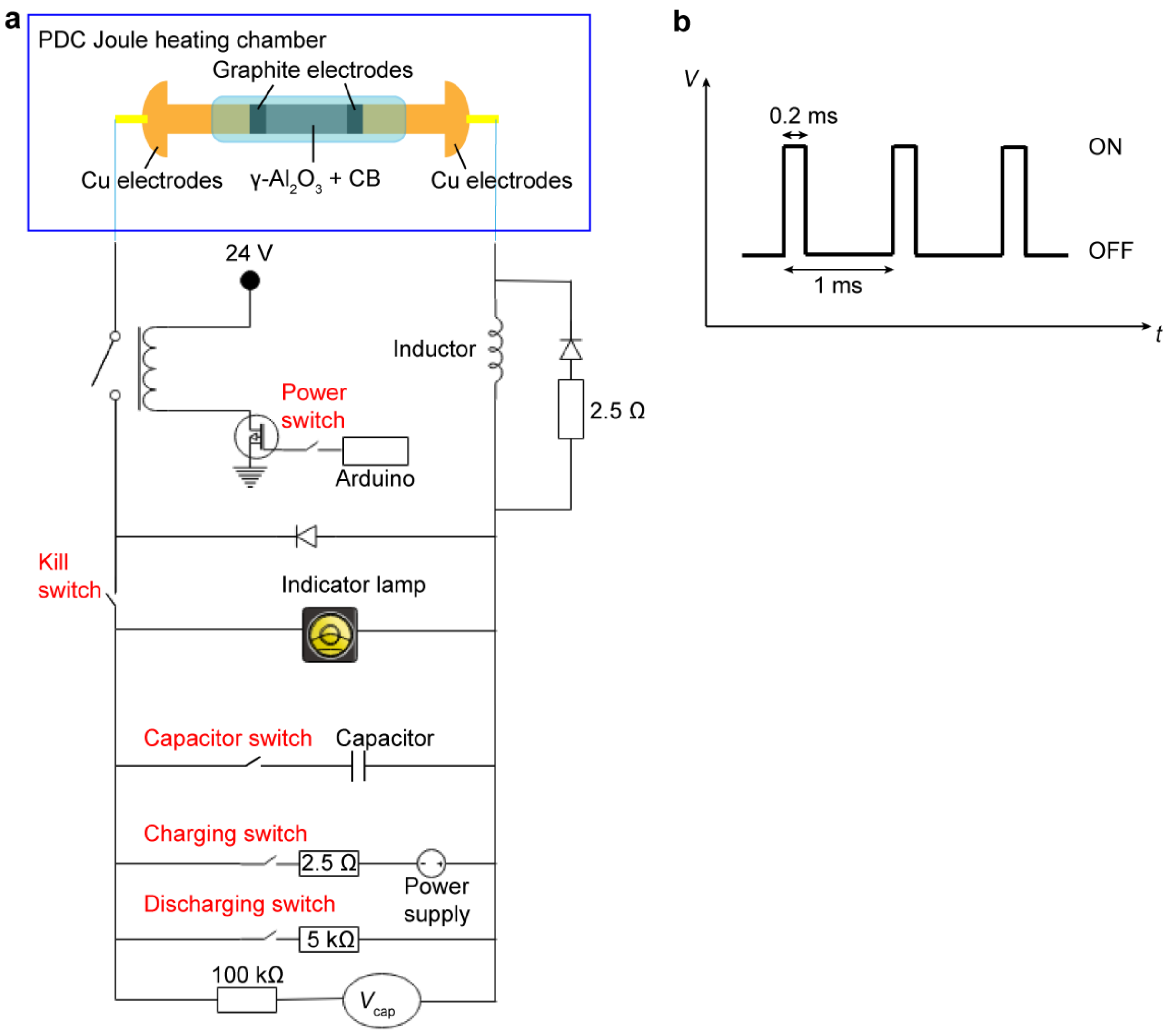

Supplementary Fig. 1. The pulsed direct current (PDC) Joule heating system. (**a**) Electrical diagram. 48 aluminum electrolytic capacitors (500 V, 13 mF) with total capacitance of 0.624 F were used for charging. Additional details of the electrical components could be found in our previous publication[8]. (**b**) Pulsed voltage generation. The frequency is 1000 Hz, and the ON state is set to be 20%, which gives a 0.2 ms voltage pulse.

CAUTION: There is a risk of electrical shock if improperly operated. The below safety instructions are recommended:

1. Enclose or carefully insulate the wire connections.
2. All connections and wires must be suitable for high voltages and currents.
3. Users should obey the one hand rule: use only one hand when working on the system, with the other hand not touching any grounded surface.
4. Keep in mind that the system can discharge thousands of Joules in milliseconds, which could cause components such as relays to explode.
5. Keep a voltmeter with high voltages test available at all times. When working on the capacitor banks, always check the voltage on each.
6. Wear thick rubber gloves that extending to the elbows when using the apparatus to protect from electrocution.
7. The reliability and robustness of the system should be confirmed by an experienced electrical technician.

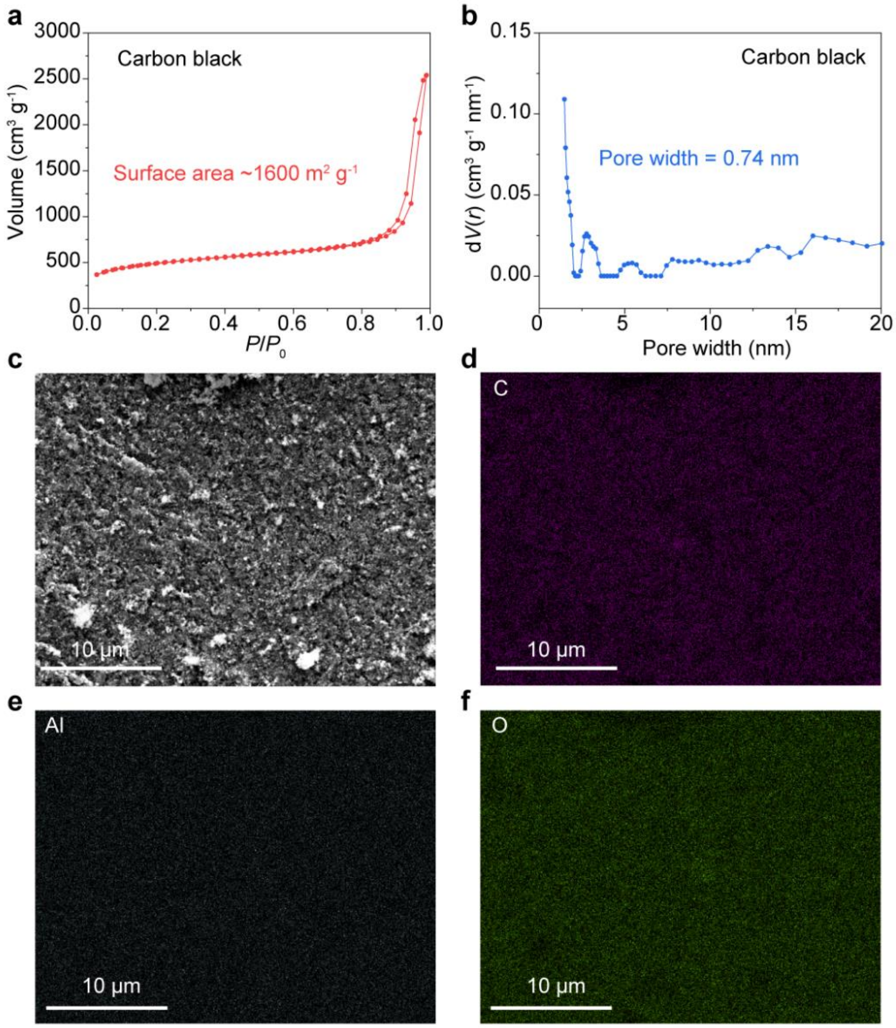

Supplementary Fig. 2. The mixing of carbon black and γ-Al_2O_3 precursors. (**a**) N_2 adsorption-desorption isotherm of carbon black (CB) at 77 K. The surface area is measured to be ~1600 m^2 g^{-1}. (**b**) Pore width distribution of CB determined by the application of the density functional theory (DFT) model to the N_2 isotherm. The pore width shows a wide distribution with maximum probability at ~0.74 nm. (**c**) Scanning electron microscopy (SEM) image of the mixture of γ-Al_2O_3 and CB. (**d-f**) Energy dispersive spectroscopy (EDS) maps of C (**d**), Al (**e**), and O (**f**) corresponding to the SEM image (**c**).

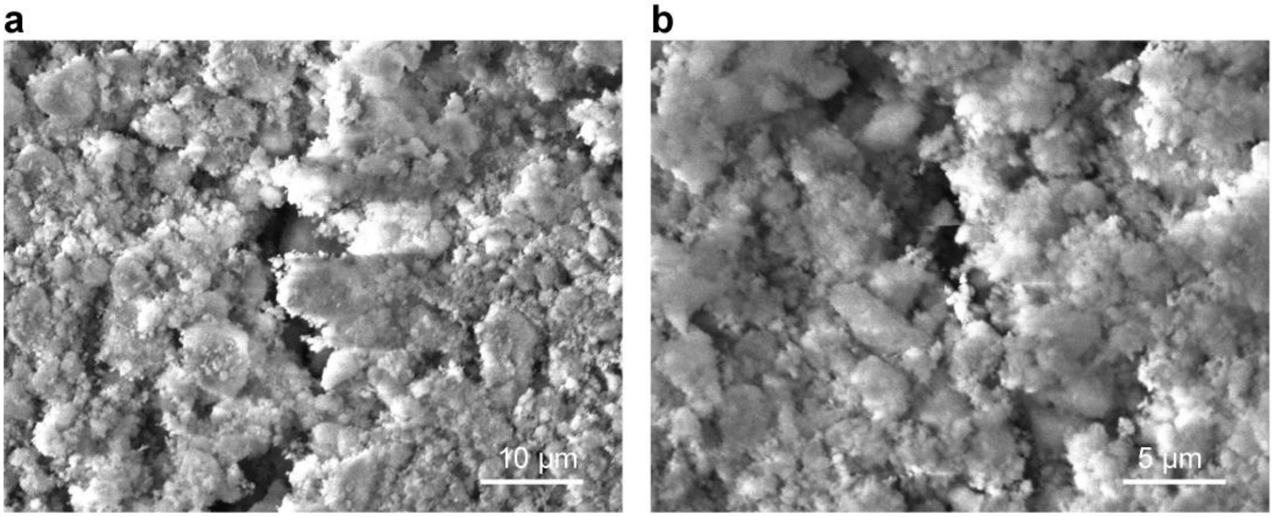

Supplementary Fig. 3. Scanning electron microscopy (SEM) images of the commercial γ-Al_2O_3 NPs precursors. (**a-b**)The SEM images of γ-Al_2O_3 NPs, showing that these nanoparticles are uniform without macroscale aggregate.

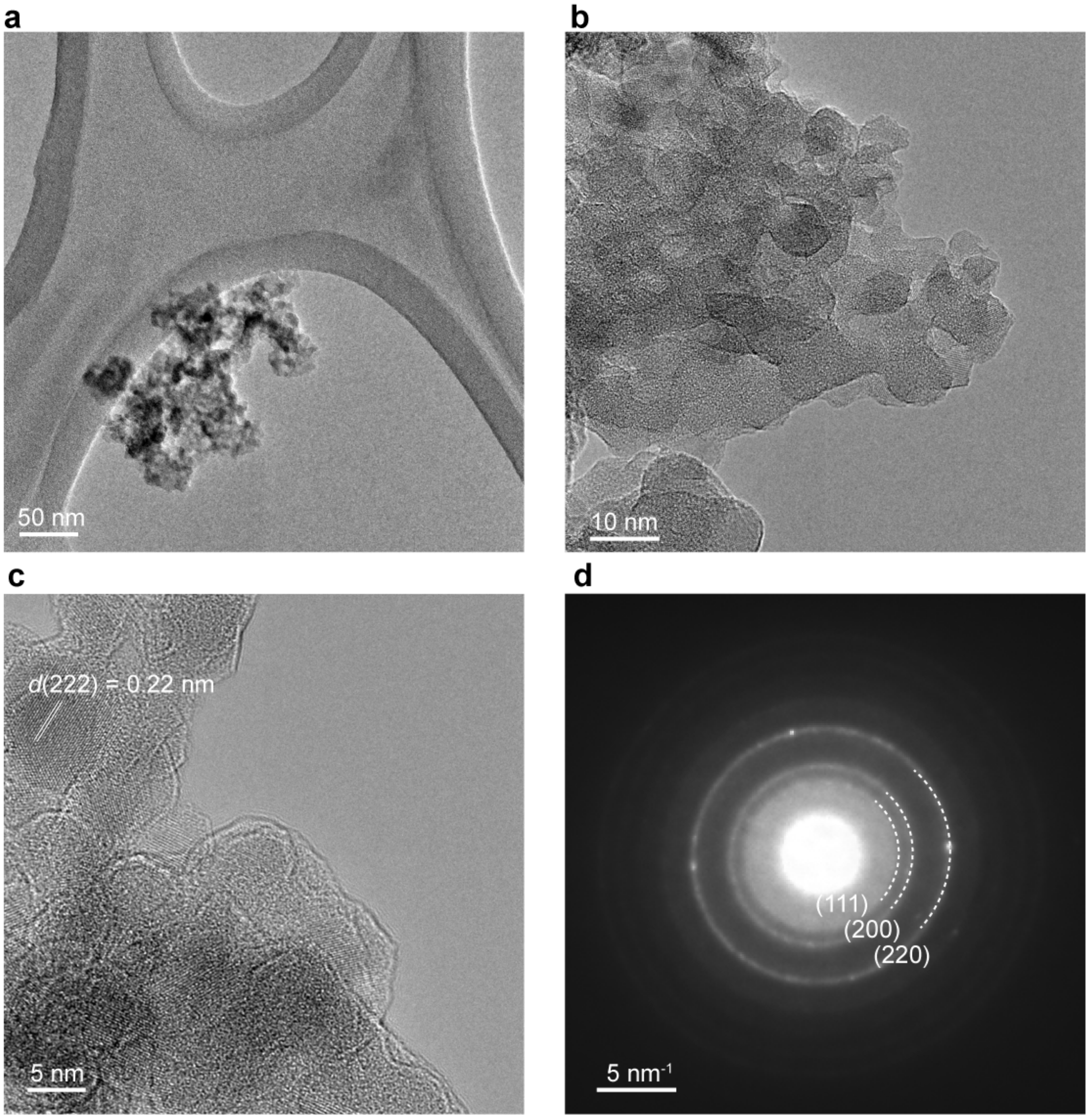

Supplementary Fig. 4. Transmission electron microscopy (TEM) characterization of the commercial γ-Al_2O_3 NPs precursors. (**a**) Bright field TEM (BF-TEM) image of γ-Al_2O_3 NPs. (**b**) High-resolution TEM (HRTEM) image of γ-Al_2O_3 NPs. The average particle size is 5-10 nm. (**c**) Enlarged HRTEM image of γ-Al_2O_3 NPs. The interplanar spacing of 0.22 nm matched well with the *d*(222) of γ-Al_2O_3. (**d**) Selected area electron diffraction (SAED) pattern of the γ-Al_2O_3 NPs. The dashed lines depict the diffraction rings of γ-Al_2O_3.

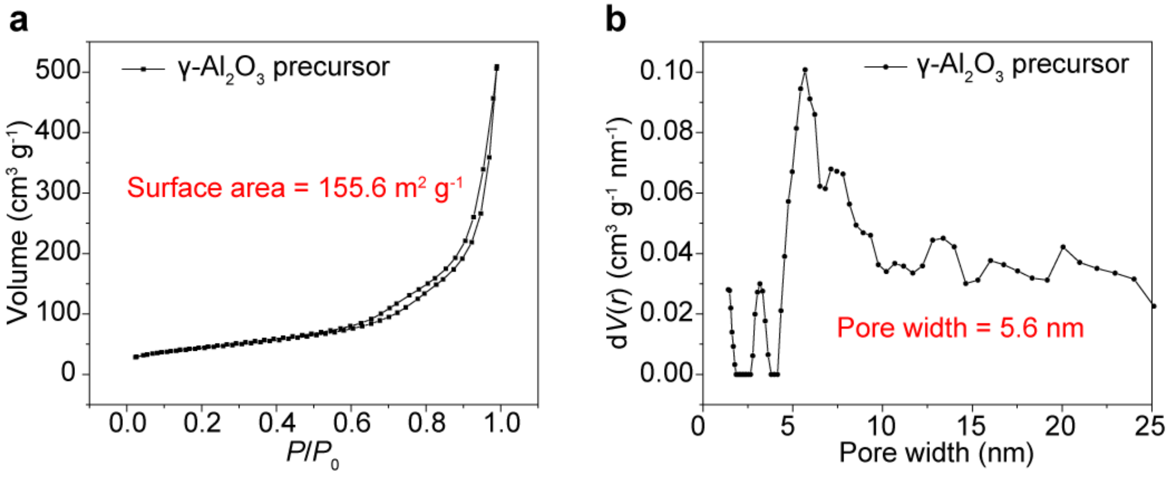

Supplementary Fig. 5. Brunauer-Emmett-Teller (BET) measurement of the γ-Al_2O_3 NPs precursors. (**a**) N_2 adsorption-desorption isotherm at 77 K. The surface area is measured to be ~155.6 m^2 g^{-1}. (**b**) Pore width distribution determined by the application of the density functional theory (DFT) model to the N_2 isotherm. The pore width shows a wide distribution with maximum probability at ~5.6 nm, consistent with the size observed by transmission electron microscopy (TEM) (Supplementary Fig. 4).

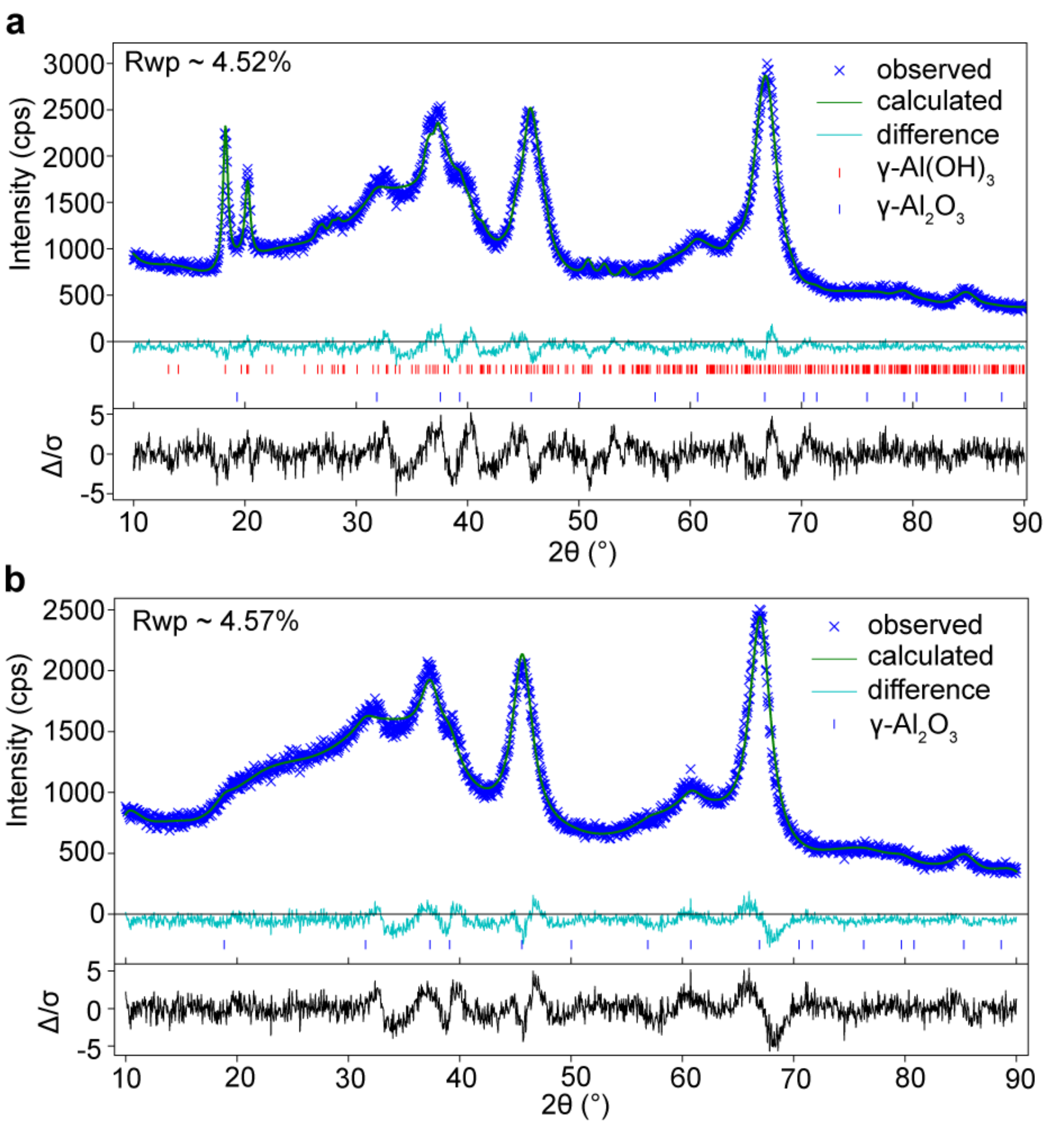

Supplementary Fig. 6. Rietveld refinement of the starting materials. (**a**) X-ray diffraction (XRD) pattern and refinement for the starting materials. The γ-$Al(OH)_3$ phase (gibbsite, crystal system: monoclinic; space group: P21/n; PDF No. 07-0324) and γ-Al_2O_3 phase (crystal system: cubic; space group: Fd-3m; PDF No. 10-0425) are plotted as reference. The Rwp value of ~4.52% demonstrates a good convergence of the refinement. (**b**) XRD pattern and refinement for the starting materials after mild calcination (in air, 700 °C for 1 h) to remove the gibbsite. The γ-Al_2O_3 phase (crystal system: cubic; space group: Fd-3m; PDF No. 10-0425) is plotted as reference. The Rwp value of ~4.57% demonstrates a good convergence of the refinement.

Discussion: The refinement shows that the starting materials are composed of ~91 wt% γ-Al_2O_3 phase and ~9 wt% γ-$Al(OH)_3$ phase (Supplementary Fig. 6a). The crystalline size of the γ-Al_2O_3 phase is estimated to be ~4 nm, and γ-$Al(OH)_3$ being ~23 nm. No other phase (e.g., δ-Al_2O_3 phase) is identified. The γ-$Al(OH)_3$ phase is easily to be converted to γ-Al_2O_3 by a mild condition calcination (Supplementary Fig. 9), or by 0.3-s PDC Joule heating (Fig. 1c). We then conducted the Rietveld refinement of the γ-Al_2O_3 phase obtained by calcination in air at 700 °C for 1 h (Supplementary Fig. 6b). The refinement shows the pure phase of γ-Al_2O_3. No other phase (e.g., δ-Al_2O_3 phase) is identified. The crystalline size of the γ-Al_2O_3 remains the same of ~4 nm. We note that the conversion of γ-$Al(OH)_3$ to γ-Al_2O_3 happens before the phase transformation of γ- to δ′-Al_2O_3 phase. Hence, we will not consider the phase of γ-$Al(OH)_3$ when explaining the phase transformation pathway from γ- to δ′- and then to α-Al_2O_3 phase.

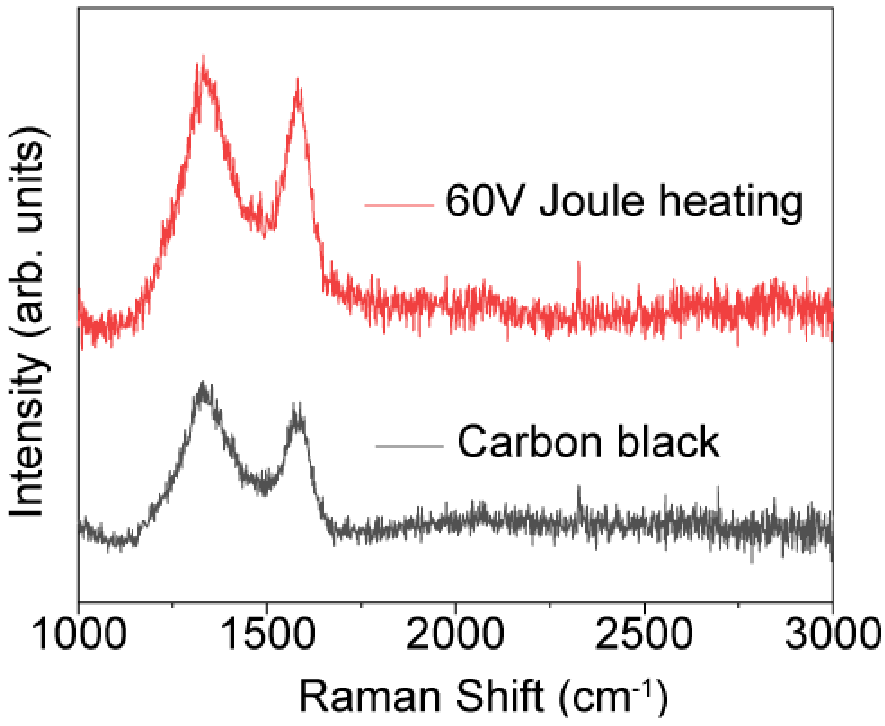

Supplementary Fig. 7. Raman spectra of carbon black (CB) precursor and the product after pulsed direct current (PDC) Joule heating at 60 V for 0.8 s. There was no observation of the 2D peaks for the product after PDC Joule heating at 60 V, indicating that the low-voltage PDC Joule heating process did not provide enough energy to graphitize the CB.

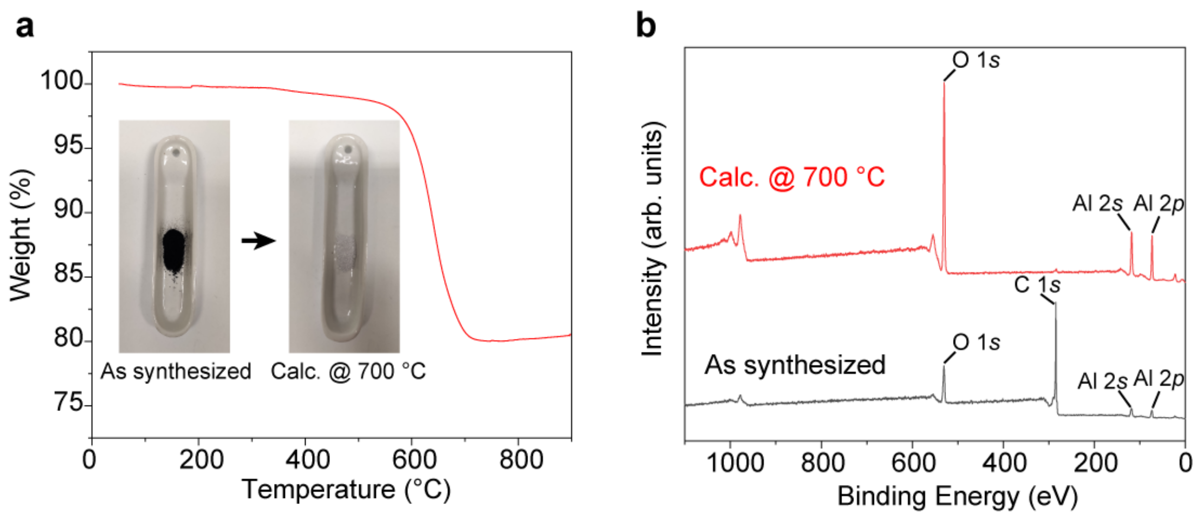

Supplementary Fig. 8. Removal of the carbon black (CB) by calcination in air. (**a**) Thermogravimetric analysis (TGA) curve of the mixture of α-Al_2O_3 and CB in air at a heating rate of 10 °C min^{-1}. Inset, the picture of as-synthesized mixture of α-Al_2O_3 and CB (left), and the purified α-Al_2O_3 NPs after calcination (right). (**b**) X-ray photoemission spectroscopy (XPS) full spectra of the mixture of α-Al_2O_3 and CB, and the α-Al_2O_3 product after calcination at 700 °C in air for 1 h. As-synthesized mixture of α-Al_2O_3 and CB exhibits strong C peaks, while after calcination, the purified α-Al_2O_3 NPs only shows a small peak of C, which might be derived from the adsorbed hydrocarbon in air.

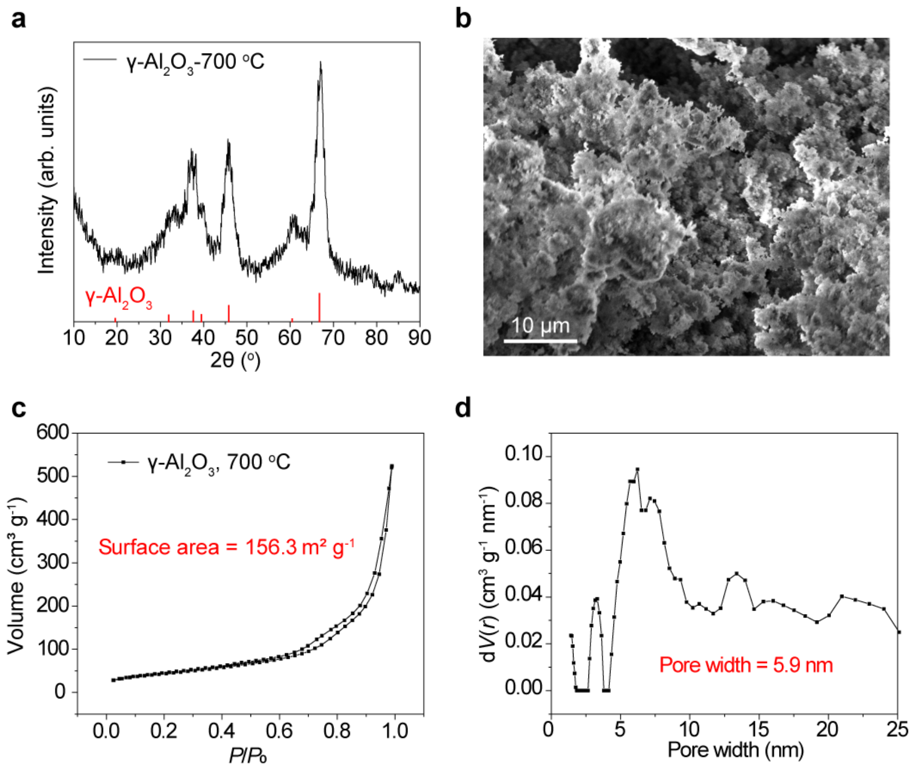

Supplementary Fig. 9. Characterization of the γ-Al_2O_3 NPs after calcination at 700 °C in air for 1 h. (**a**) X-ray diffraction (XRD) pattern. The reference PDF card for γ-Al_2O_3: No. 10-0425. (**b**) SEM image. (**c**) N_2 adsorption-desorption isotherms at 77 K. (**d**) Pore width distribution determined by the application of density functional theory model to the N_2 isotherm.

The XRD pattern of the γ-Al_2O_3 NPs precursors (Fig. 1c, 0 s) shows that the composition of the precursor is mainly γ-Al_2O_3 phase with minor content of γ-$Al(OH)_3$. After calcination (in air, 700 °C for 1 h), the XRD pattern (Supplementary Fig. 9a) shows the disappearance of γ-$Al(OH)_3$ and the retention of γ-Al_2O_3 phase. No other phase is detected. The surface area and pore width of the calcined samples are ~156 m^2 g^{-1} and ~5.9 nm, respectively, which are comparable to the values of the γ-Al_2O_3 precursors at ~156 m^2 g^{-1} and ~5.6 nm, respectively (Supplementary Fig. 5). These results show that the calcination process, 700 °C in air for 1 h, does not trigger the phase transformation and has negligible effect on the coarsening or aggregation of the γ-Al_2O_3 phase.

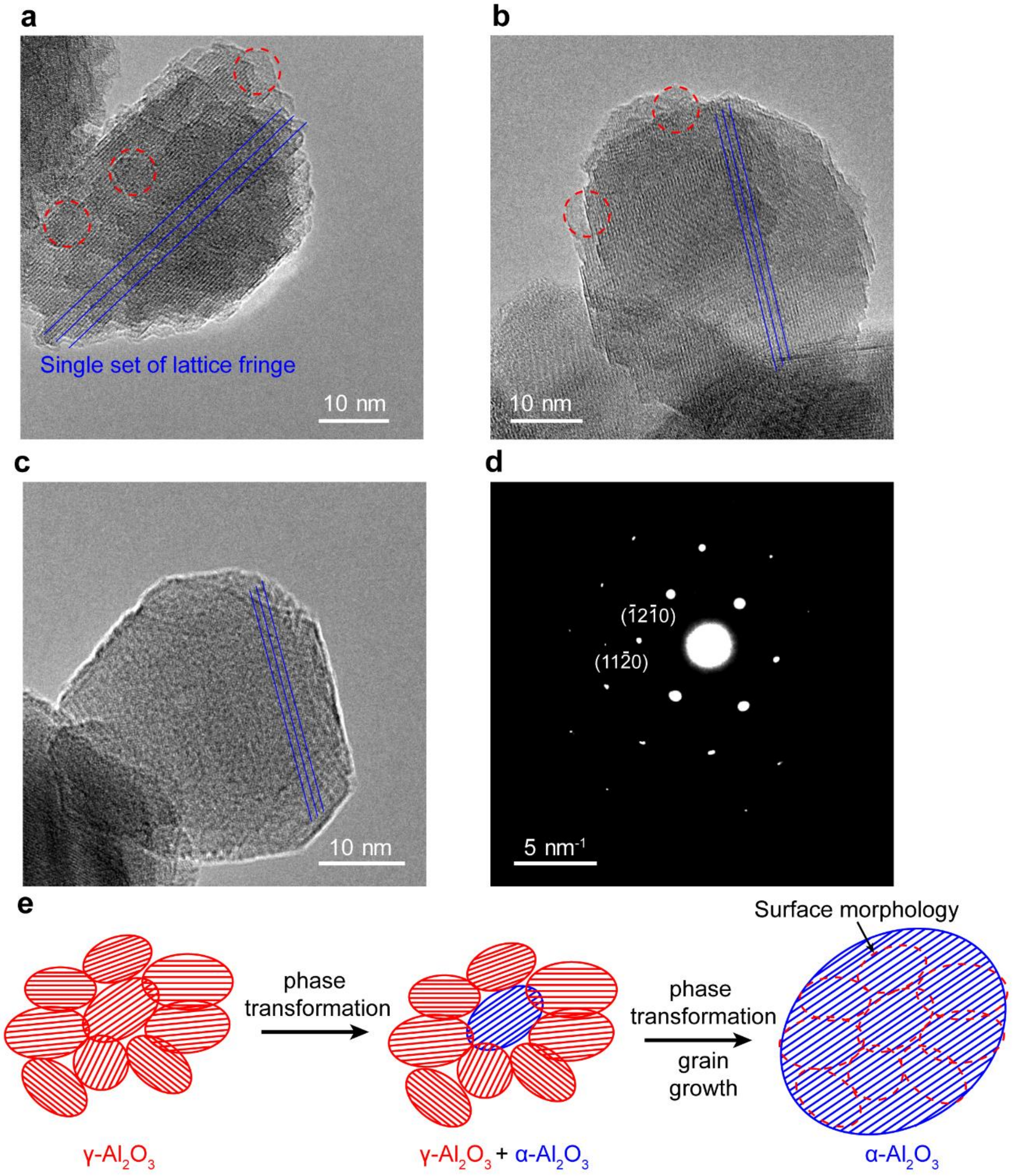

Supplementary Fig. 10. Transmission electron microscopy (TEM) characterization of the α-Al_2O_3 NPs product. (**a-c**) High-resolution TEM (HRTEM) images of the α-Al_2O_3 NPs. The red dash circles showed the surface roughness feature of the NPs. The blue lines denoted the lattice fringe of the NPs. (**d**) Nanobeam diffraction (NBD) pattern of a single α-Al_2O_3 NP. (**e**) The schematic of the phase transformation and grain growth process.

Discussion: The particle size of the γ-Al_2O_3 NPs precursor is 5–10 nm (Supplementary Fig. 4). The HRTEM images of the α-Al_2O_3 NPs in Supplementary Fig. 10a show the particle size of 20–30 nm. The single set of lattice fringe (blue lines in Supplementary Figs. 10a-c) across the entire particle demonstrates the single-crystal feature of the particle. The single set of diffraction pattern by NBD also explicitly proves the single-crystal feature of the NP (Supplementary Fig. 10d). We can observe the surface morphology feature on the nanoparticles (red circles in Supplementary Fig. 10a), which is presumed to be retained from the particle fusing of the of γ-Al_2O_3 NPs precursors. During the PDC Joule heating process, the γ-Al_2O_3 NPs undergo both phase transformation and grain growth. The two processes inevitably happen simultaneously upon heating. One γ-Al_2O_3 NP undergoes phase transformation and nucleates as α-Al_2O_3 NP (Supplementary Fig. 10e, middle). At the same time, the α-Al_2O_3 NP nucleus consumes a few nearby γ-Al_2O_3 NPs and becomes a larger particle (Supplementary Fig. 10e, right). During the process, the morphology feature of the γ-Al_2O_3 NP is retained (red dash circles in Supplementary Fig. 10e, right). We note that the grain growth is inevitable for any thermal process. For the conventional extended high-temperature thermal annealing process, like furnace annealing, the grain coarsening is more detrimental, so the resulting α-Al_2O_3 has a surface area well below 10 m^2 g^{-1}. In contrast, since our PDC process is ultrafast and the nanoparticles are locally heated due to the hotspot effect, the mass transfer and grain coarsening during the phase transformation process are, to a large extent, avoided and hence a high surface area of ~65 m^2 g^{-1} is maintained.

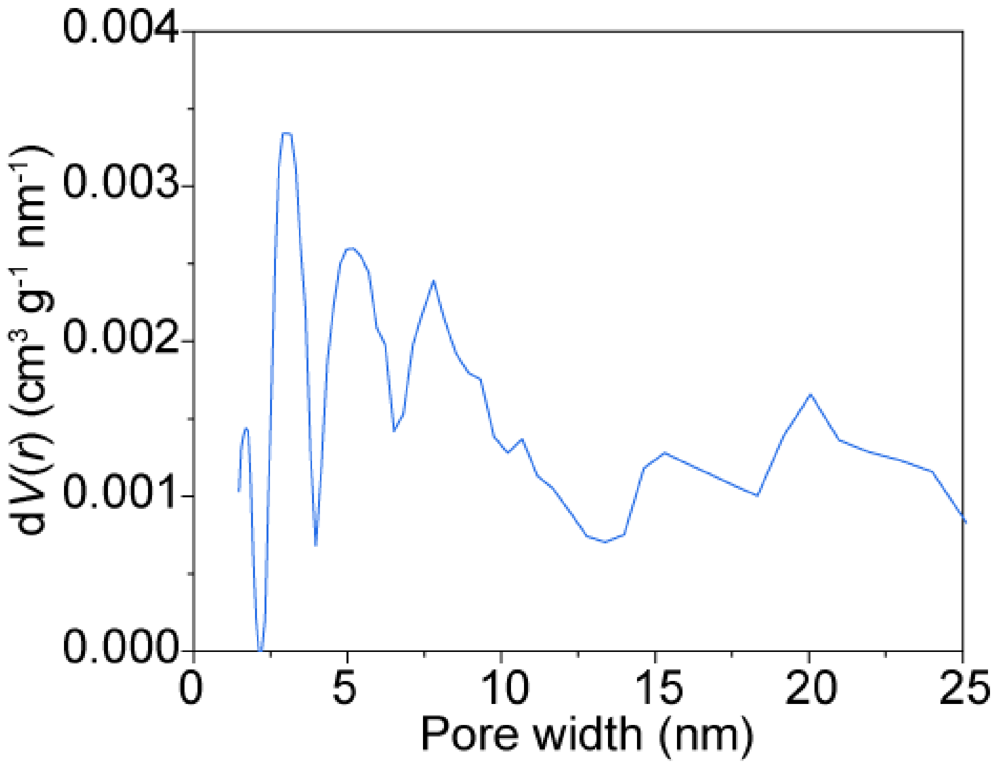

Supplementary Fig. 11. Pore width distribution of another sample with specific surface area ~70 m^2 g^{-1}. Pore width distribution determined by application of the density functional theory (DFT) model to the N_2 isotherm.

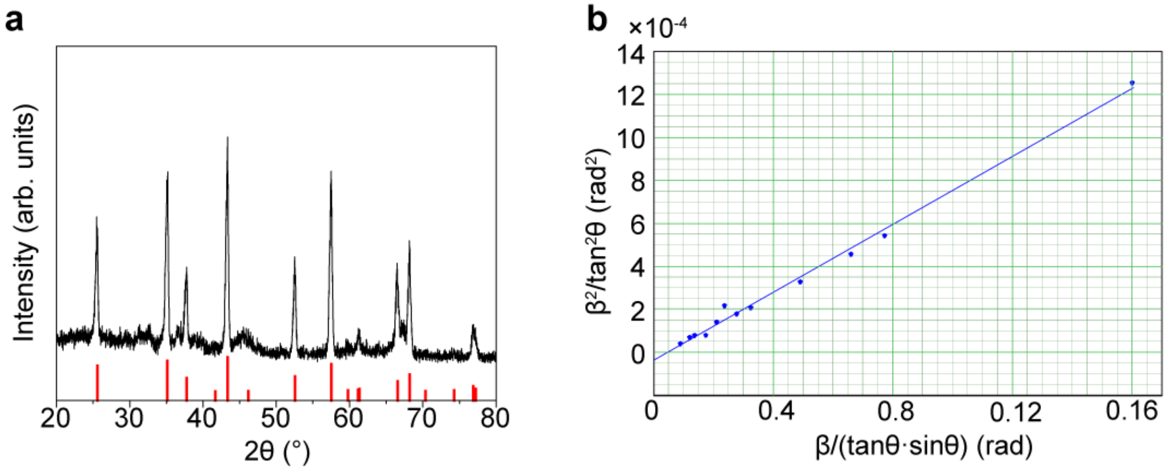

Supplementary Fig. 12. Crystalline size determination based on Halder-Wagner's method. (**a**) Representative X-ray diffraction (XRD) pattern of α-Al_2O_3 product. The reference PDF card for α-Al_2O_3: No. 10-0173. (**b**) The plot of $\frac{\beta^2}{tan^2\theta}$ against $\frac{\beta}{tan\theta \cdot sin\theta}$. The crystal size of the specific sample was determined to be ~21 nm.

By using this method, we have measured the crystal size of a few samples, with the crystal size of 21 nm, 23 nm, 21 nm, 22 nm, 23 nm and 22 nm. The average crystal size determined by XRD was ~22 nm. This value matches well with the average particle size determined by BET measurement (~23 nm), and slight smaller than that determined by TEM results (~25 nm). Since the TEM has a much smaller sample amount than BET and XRD, we used the BET results of ~23 nm as the average particle size of the α-Al_2O_3 product.

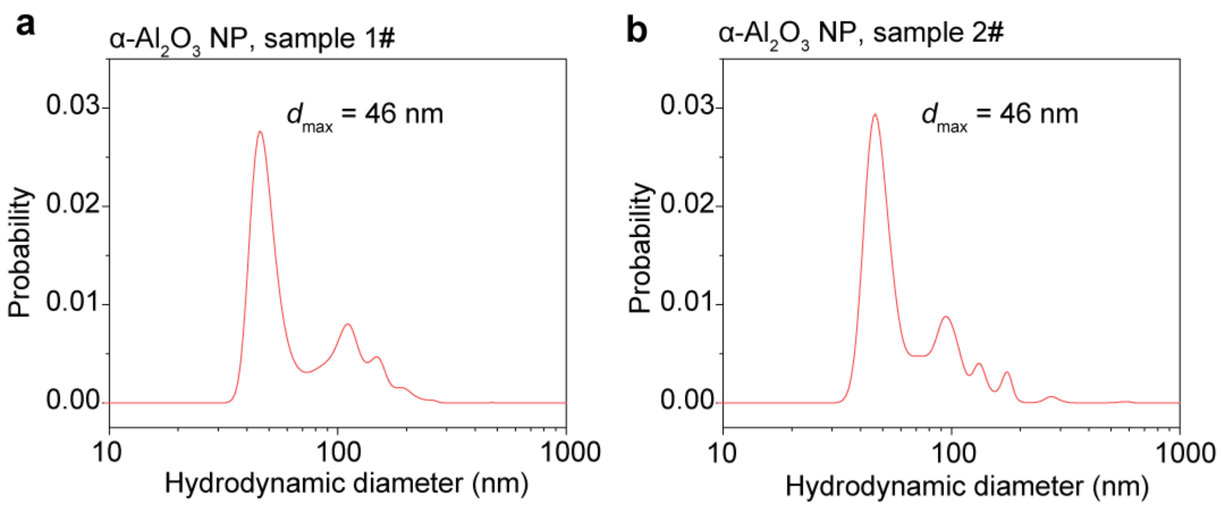

Supplementary Fig. 13. Dynamic light scattering (DLS) measurement of the α-Al_2O_3 NP. (**a, b**) Hydrodynamic diameter distribution of the α-Al_2O_3 NPs for sample 1# (**a**) and sample 2# (**b**).

The hydrodynamic diameter distribution shows the maximum probable diameter (d_{max}) at 46 nm. This value is larger than the particle size from the TEM statistic (~23 nm), which is originated from the different measurement method and consistent with the previous report[4]. There is a tail at the larger diameter side, which might be from some aggregate of the particles. The measurement shows that the α-Al_2O_3 NPs are dispersible.

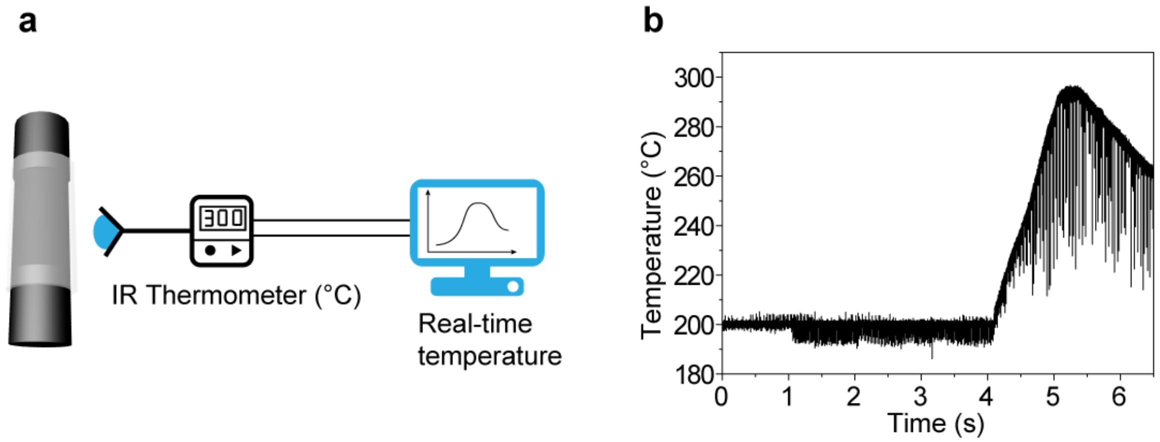

Supplementary Fig. 14. Temperature measurement of the pulsed direct current (PDC) induced phase transformation. (**a**) The scheme of the real-time temperature recording system using an IR thermometer. The temperature range of the thermometer is 200 to 1500 °C. (**b**) The temperature curve of the 60 V PDC treatment of the precursors (*f*(γ-Al_2O_3) ~0.73). The largest temperature was recorded to be ~300 °C.

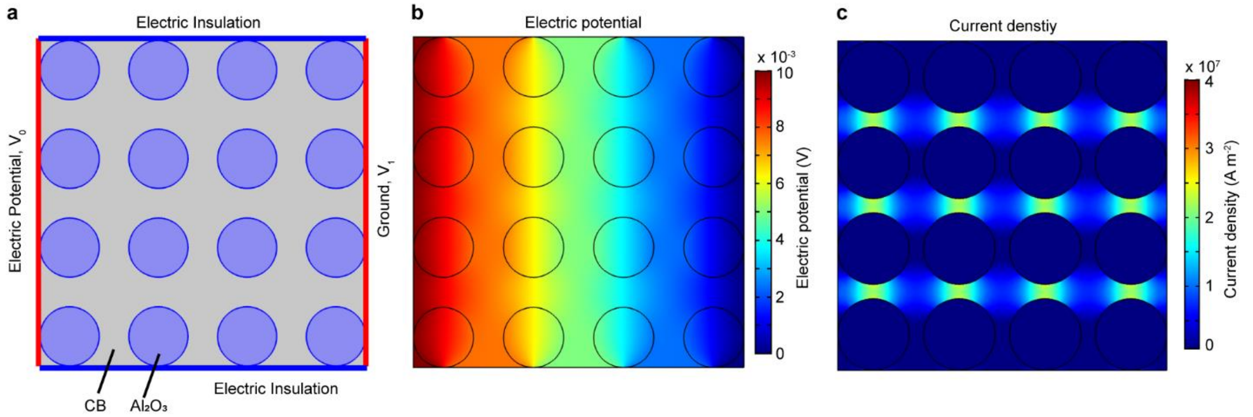

Supplementary Fig. 15. Current density simulation for *f*(γ-Al_2O_3) = 0.42. (**a**) Geometric configuration and boundary conditions. The Electric Potential, V_0, was set on the left boundary and Ground, V_1 = 0 V, on the right boundary to mimic the voltage drop of the Joule heating process. The top boundary and bottom boundary are electric insulation. The separated blue balls are the Al_2O_3 NPs, and the continuous grey regions are carbon black (CB). It is assumed that the Al_2O_3 NPs are uniformly distributed in the CB matrix. (**b**) Electric potential map. The electric potential is nearly linear decreased from the Electric Potential boundary to the Ground boundary. (**c**) Current density map. In the bulk of the Al_2O_3 NPs, the current density is zero. In the CB region, the current density values are strongly affected by the distribution of the Al_2O_3 NPs. There are local maximum regions in the vertical gaps of the Al_2O_3 NPs, while there are local minimum regions in the horizontal gaps of the Al_2O_3 NPs.

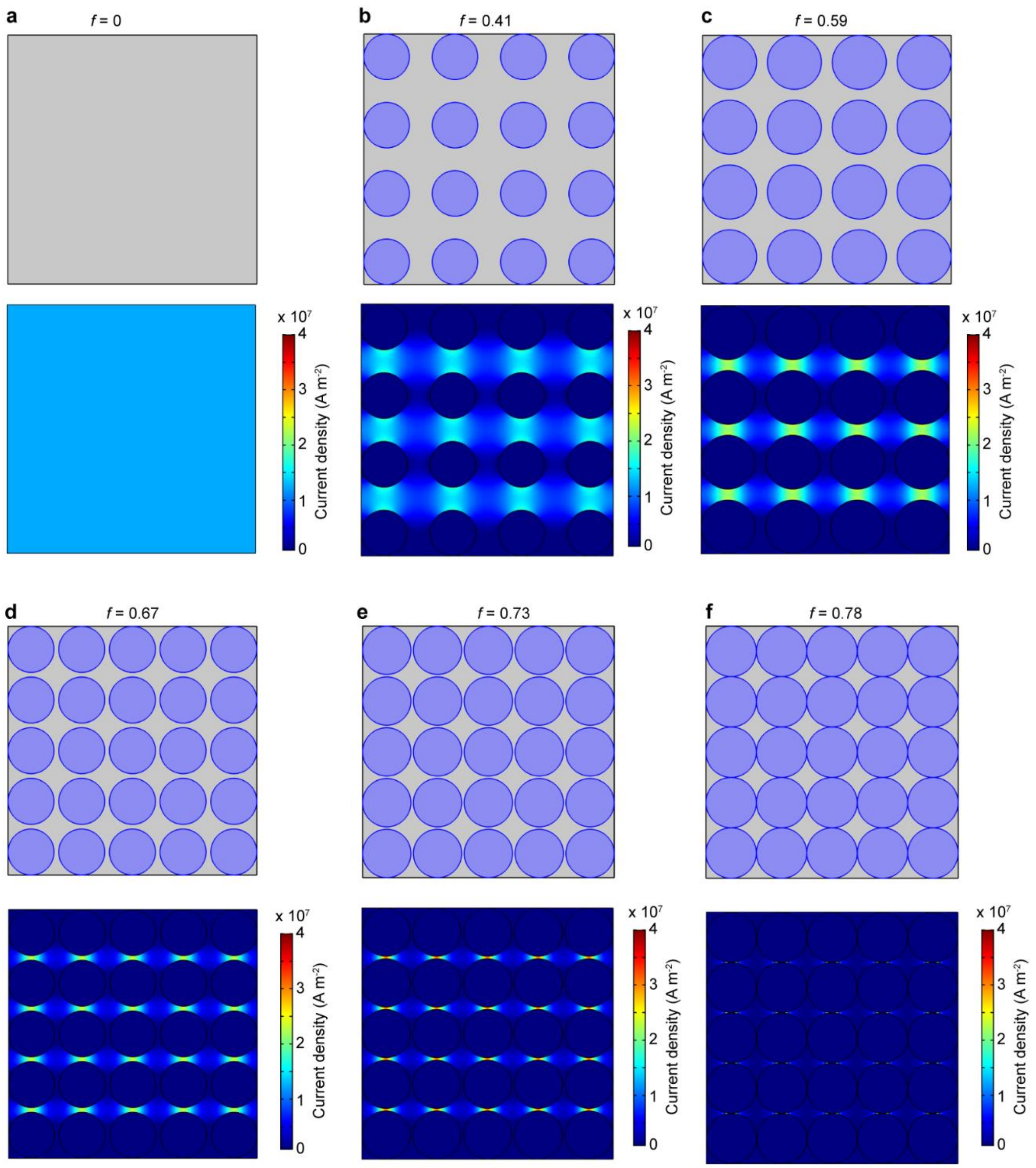

Supplementary Fig. 16. Current density simulation. Geometric configurations (upper) and current density maps (lower) for (**a**) *f*(γ-Al_2O_3) = 0, (**b**) *f*(γ-Al_2O_3) = 0.41, (**c**) *f*(γ-Al_2O_3) = 0.59, (**d**) *f*(γ-Al_2O_3) = 0.67, (**e**) *f*(γ-Al_2O_3) = 0.73, and (**f**) *f*(γ-Al_2O_3) = 0.78.

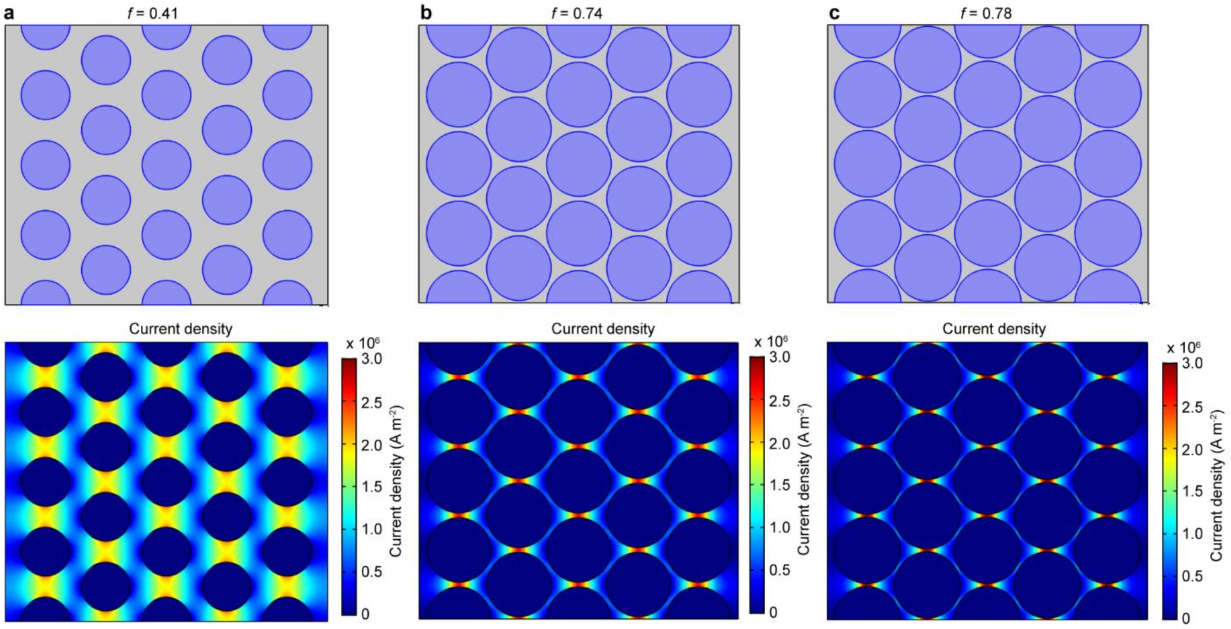

Supplementary Fig. 17. Current density simulation with a hexagonal stacking order geometric configuration. Geometric configurations (upper) and current density maps (lower) for (**a**) $f(\gamma\text{-}Al_2O_3)$ = 0.41, (**b**) $f(\gamma\text{-}Al_2O_3)$ = 0.74, and (**c**) $f(\gamma\text{-}Al_2O_3)$ = 0.78.

The blue balls represent the γ-Al_2O_3 NPs while the grey continuous regions represent the CB. The boundary conditions are similar to those of the square stacking order geometric configuration (Supplementary Fig. 15). The current maps also show local maximums, or hotspots, in the vertical gaps between γ-Al_2O_3 NPs. These results show that the hotspot effect is only affected by the gap of the γ-Al_2O_3 NPs and not related to the geometrical configuration.

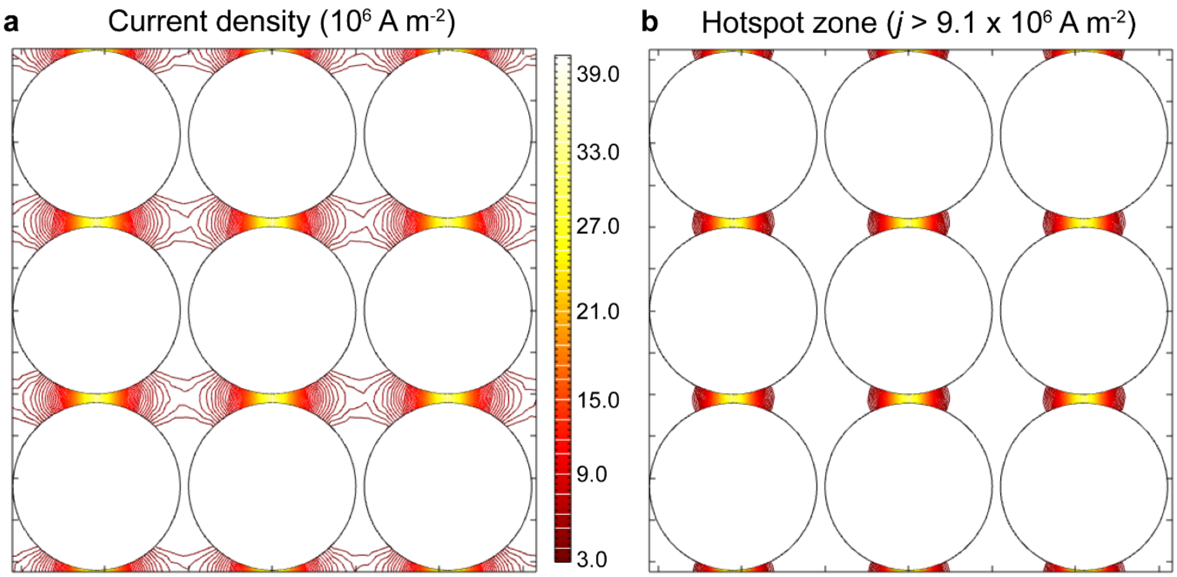

Supplementary Fig. 18. Zone size of the hotspot at *f* = 0.73. (**a**) Current density distribution map. (**b**) Hotspot zone, defined as the region with $j \geq 9.1 \times 10^6$ A m^{-2}. The circles indicate the Al_2O_3 nanoparticles, and the other regions indicates the continuous conductive carbon black.

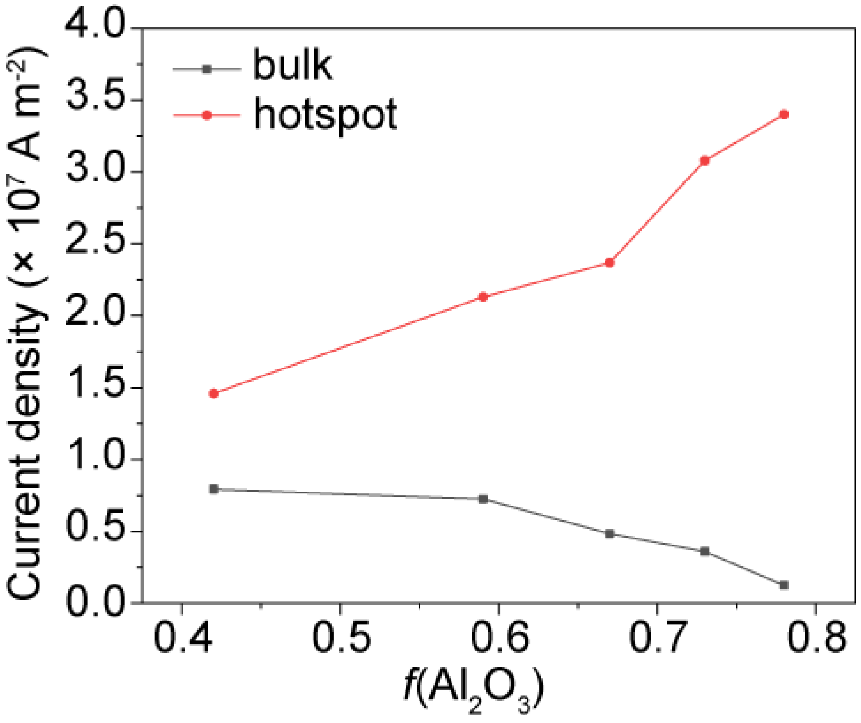

Supplementary Fig. 19. Current density at the bulk regions and the hotspot regions. The quantitative analysis indicates a decreased current density in bulk and an increased current density in hotspots as increasing the f(γ-Al_2O_3).

In addition to the absolute value of the current density in the hotspot, the zone size of the hotspot region should also be considered. Although the sample with f(γ-Al_2O_3) = 0.78 has the largest current density, the current density map shows that the hotspot region is much smaller than other samples (Supplementary Fig. 16). In this case, it could not trigger the phase transformation.

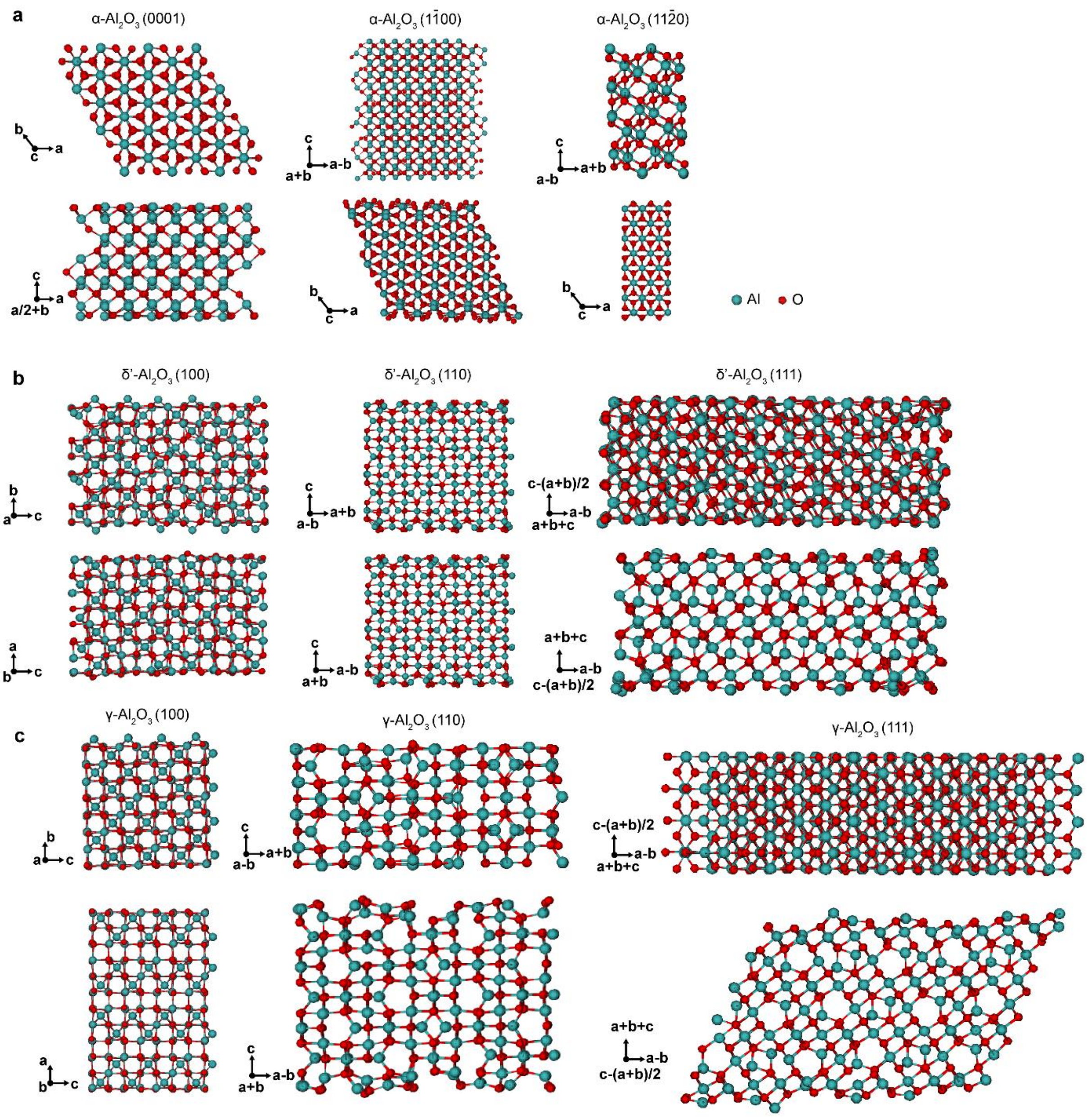

Supplementary Fig. 20. Optimized atomic structures of α-Al_2O_3, δ′-Al_2O_3, and γ-Al_2O_3 surfaces based on DFT modeling. (**a**) Atomic models of (0001), (1$\bar{1}$00), and (11$\bar{2}$0) surfaces of α-Al_2O_3 from top view (top) and lateral view (bottom). (**b**) Atomic models of (100), (110), and (111) surfaces of δ′-Al_2O_3 from top view (top) and lateral view (bottom). (**c**) Atomic models of (100), (110), and (111) surfaces of γ-Al_2O_3 from top view (top) and lateral view (bottom).

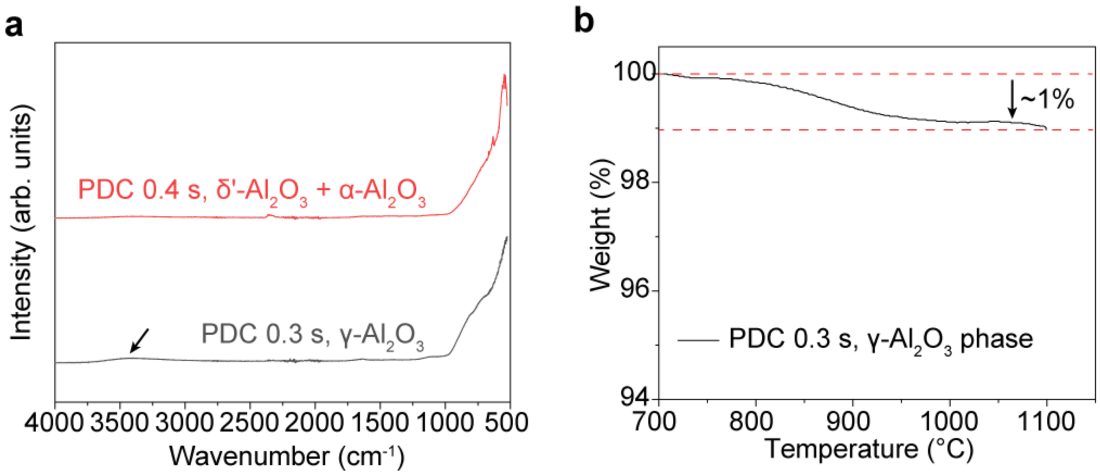

Supplementary Fig. 21. Surface hydroxyl group on γ-Al_2O_3. (**a**) Fourier-transform infrared spectroscopy (FT-IR) spectra of the γ-Al_2O_3 after pulsed direct current (PDC) Joule heating duration of 0.3 s, and the obtained δ′-Al_2O_3 and α-Al_2O_3 by PDC Joule heating duration of 0.4 s. The black arrow points to the hydroxyl group absorbance. (**b**) Thermogravimetric analysis (TGA) curve of the γ-Al_2O_3 after PDC Joule heating duration of 0.3 s. TGA was conducted in N_2 with the heating rate of 10 °C min^{-1}. The dashed lines denote the weight change during the TGA.

Discussion: we measured the hydroxyl group density on the γ-Al_2O_3 surface by TGA (Supplementary Fig. 21b). The OH groups could chemically adsorb on the surface of γ-Al_2O_3 at room temperature. The phase transformation from γ-Al_2O_3 to δ′-Al_2O_3 usually initiates at ~700 °C (ref.[9]); hence, it is reasonable to consider the surface OH group density of γ-Al_2O_3 at 700 °C to avoid overestimation. The weight loss was measured to be ~1% from 700 °C to 1100 °C, which was resulted by the loss of H_2O. The OH density on the γ-Al_2O_3 surface was calculated to be ~2 OH nm^{-2} according to its surface area of ~156 m^2 g^{-1} (Supplementary Fig. 9).

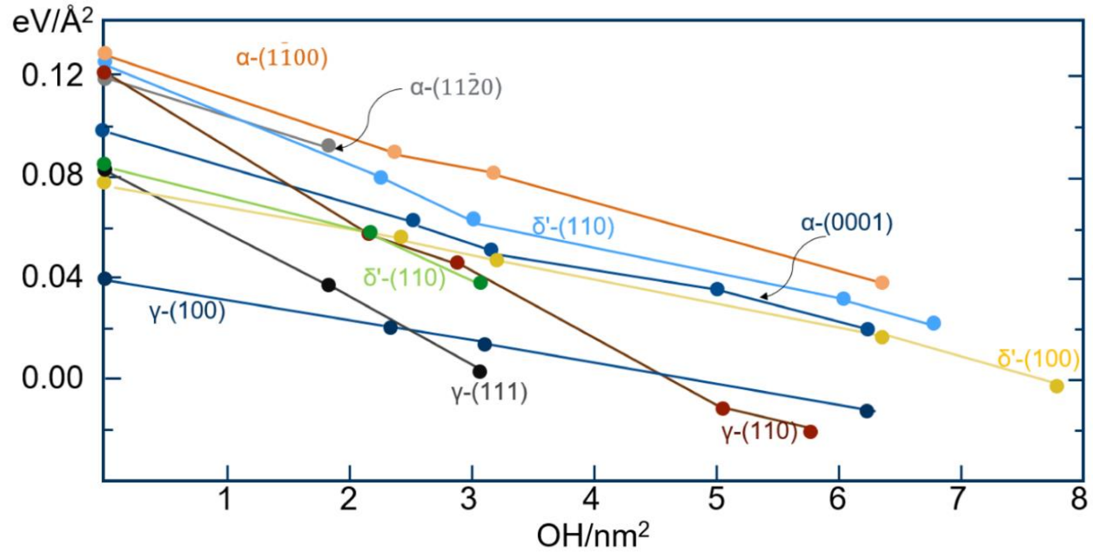

Supplementary Fig. 22. Surface energy of Al_2O_3 at various coverage of OH adsorption. All three phases and their representative surfaces are calculated.

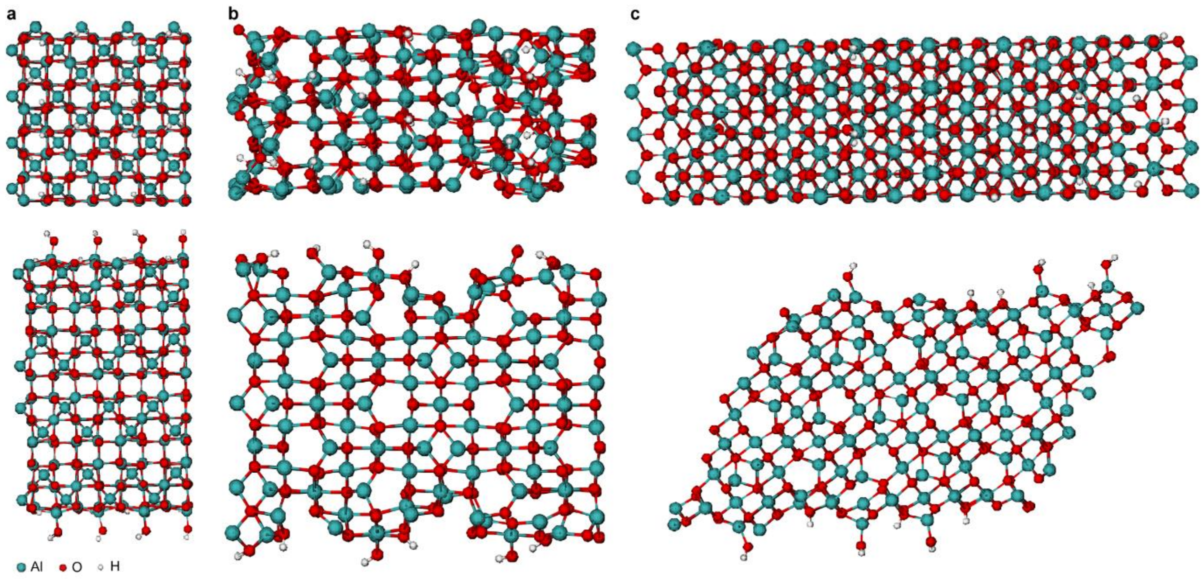

Supplementary Fig. 23. Top and side views of atomic models for γ-Al_2O_3 surfaces with OH groups. (**a**) (100) surfaces with 2.3 OH nm^{-2}. (**b**) (110) surface with 2.2 OH nm^{-2}. (**c**) (111) surfaces with 1.8 OH nm^{-2}.

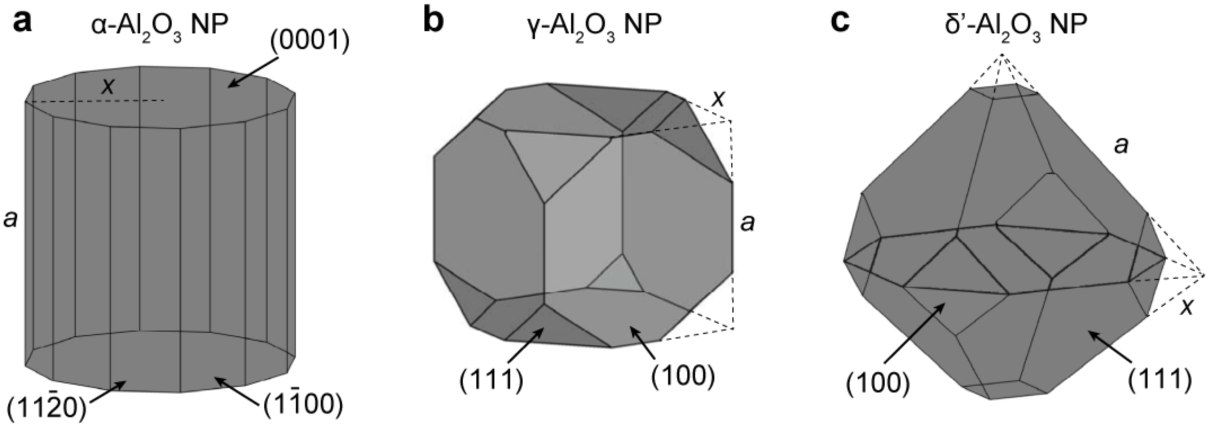

Supplementary Fig. 24. The optimization of the nanocrystal shapes. (**a**) Dodecagonal prism for α-Al_2O_3 nanocrystal. (**b**) Truncated cube for γ-Al_2O_3 nanocrystal (**c**) Truncated octahedron for δ′-Al_2O_3 nanocrystal.

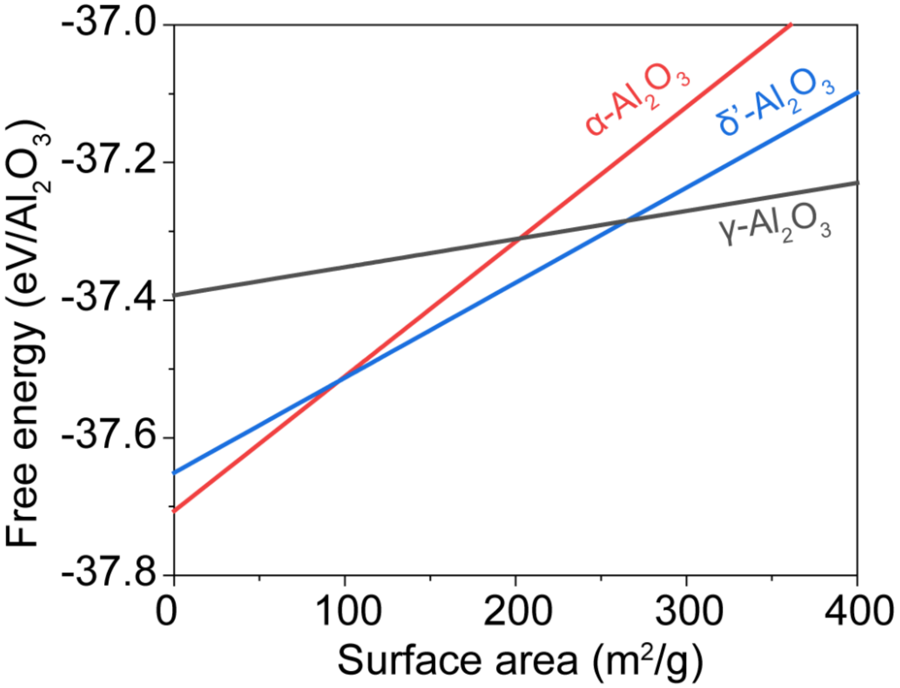

Supplementary Fig. 25. The free energy of the three phases Al_2O_3 nanocrystals. The free energy (the sum of energy and entropic contribution) of the Al_2O_3 nanocrystals of three phases as plotted against the specific surface area.

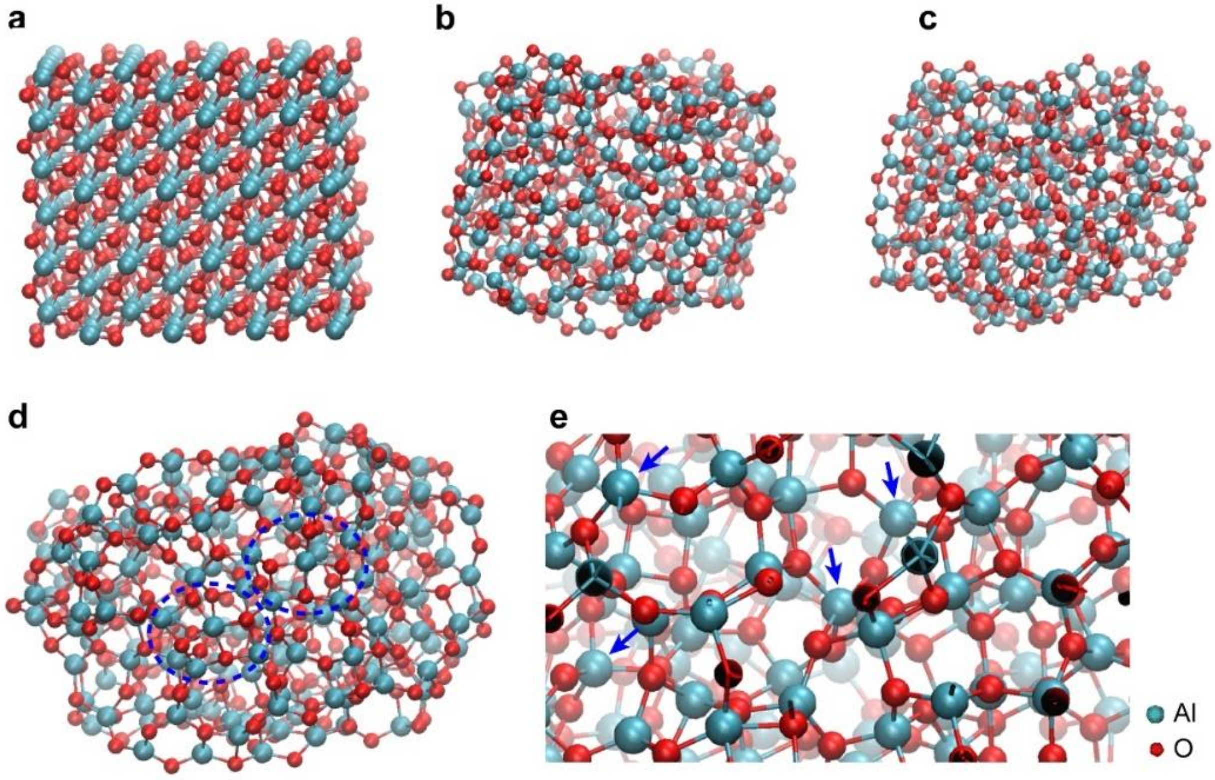

Supplementary Fig. 26. Dynamic simulations of structure transformation of an α-phase Al_2O_3 nanocrystal at 1800 K for 10 ps. (**a**) Lateral view of the α-phase Al_2O_3 nanocrystal, and (**b**) the nanocrystal after annealing for 5 ps, and (**c**)10 ps. (**d**) Top view of the nanocrystal at 10 ps. The dashed circles denote the rectangular local bonding network. (**e**) Zoom-in structure of the nanocrystal at 10 ps. The arrows denote the tetrahedral coordination of Al atoms.

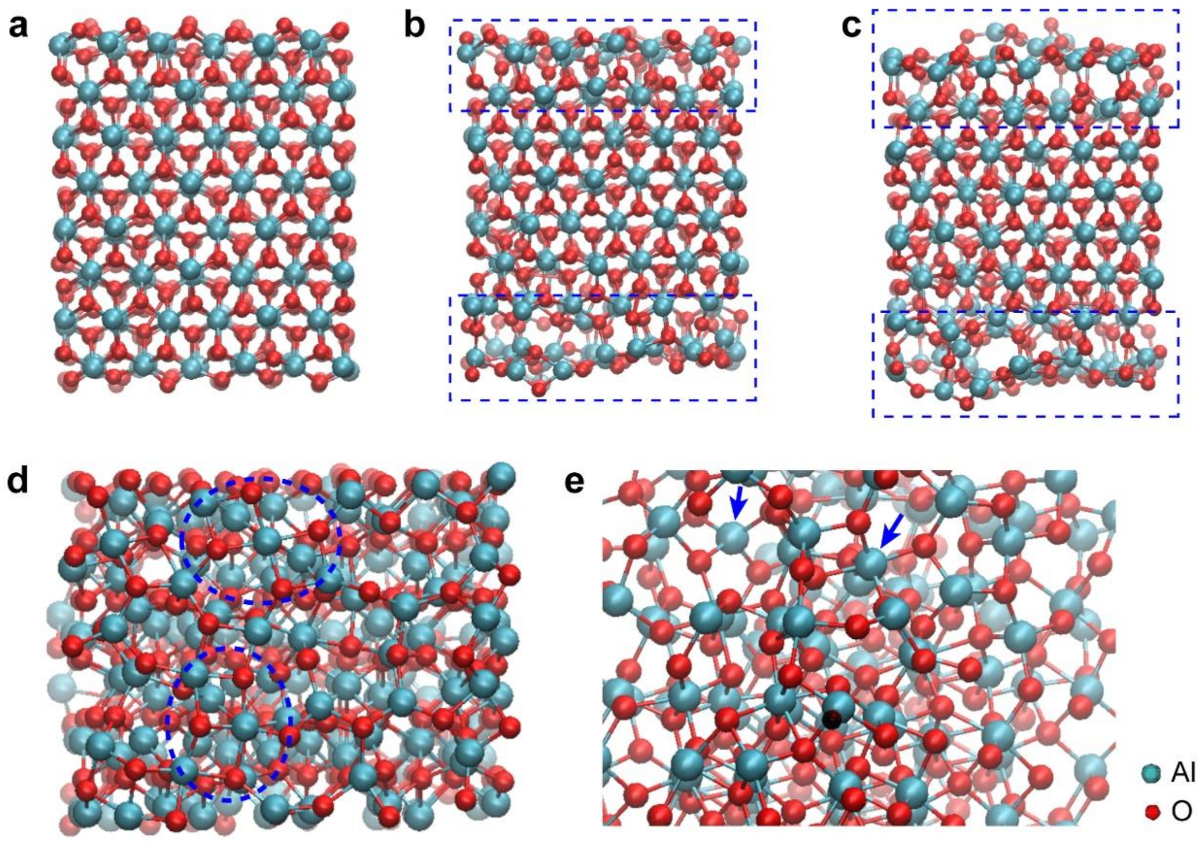

Supplementary Fig. 27. Dynamic simulation of structure transformation of an α-phase Al_2O_3 thin slab at 2000 K. (**a**) Lateral view snapshots of the α-phase Al_2O_3 slab after annealing for t = 2 ps, (**b**) 10 ps, and (**c**) 20 ps. The dashed rectangles denote the distorted surface structure. (**d**) Top view of the slab at 20 ps. The dashed circles denote the rectangular local bonding network. (**e**) Zoom-in structure of the slab at 20 ps. The arrows denote the tetrahedral coordination of Al atoms.

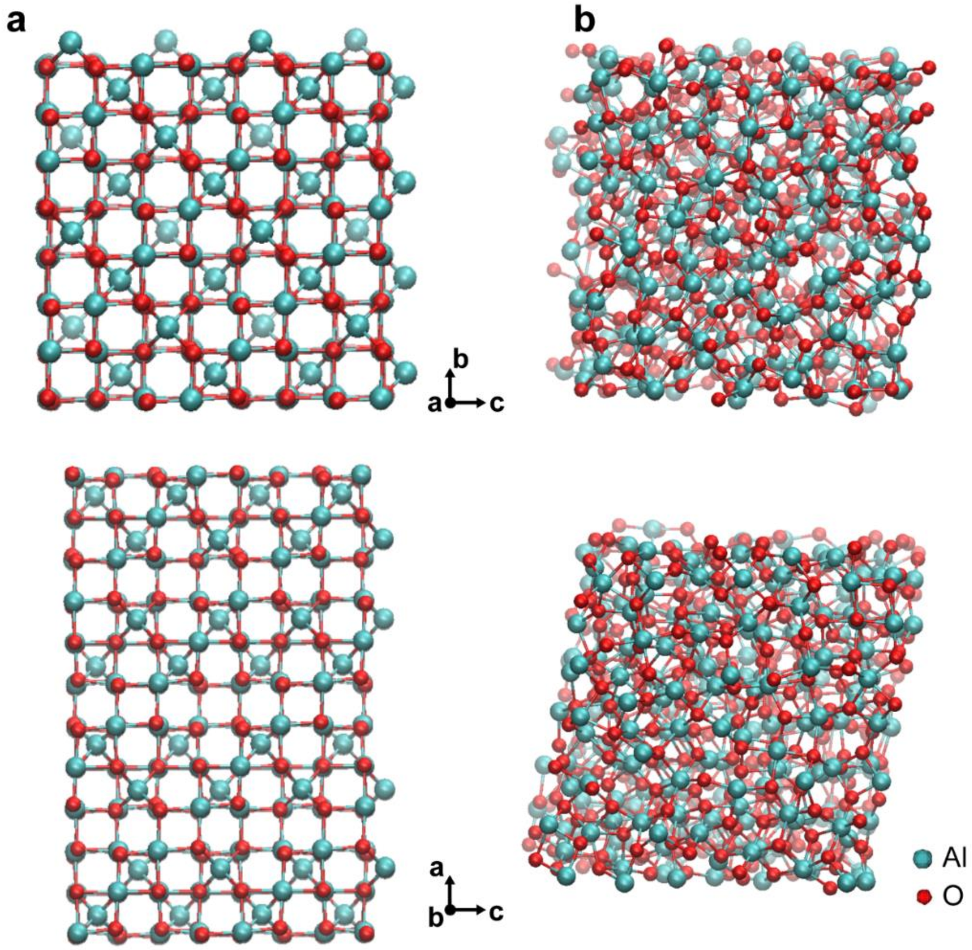

Supplementary Fig. 28. Dynamic simulation of structure transformation of γ-Al_2O_3 bulk crystal (supercell: 1.6×1.6×2.4 nm^3) at 2000 K for 30 ps. (**a**) Top and lateral views of initial crystalline γ-Al_2O_3 structure. (**b**) Top and lateral view of final amorphous structure with local order similar to α-Al_2O_3.

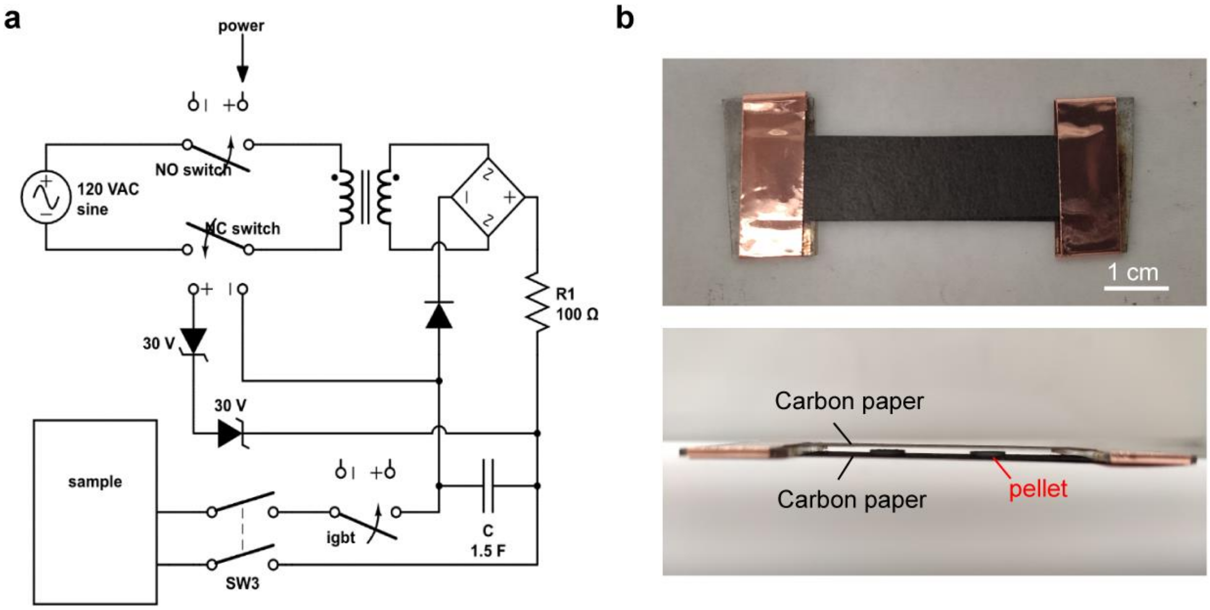

Supplementary Fig. 29. Ultrafast alternating current sintering (ACS) system and sample holder. (**a**) Electric diagram of the ACS system. The total capacitance is 1.5 F and the largest available voltage is 63 V. The capacitor is simultaneous charged by the alternating current (AC) supply and provides energy output to the samples by discharging. The energy output is continuous and enables an extended sinter of seconds with high energy output. (**b**) Picture of the carbon paper holder for sintering. Two carbon papers were attached to a glass slide and adhered by copper taps. The α-Al_2O_3 green body were put in between the carbon papers, which were connected to the ACS system.

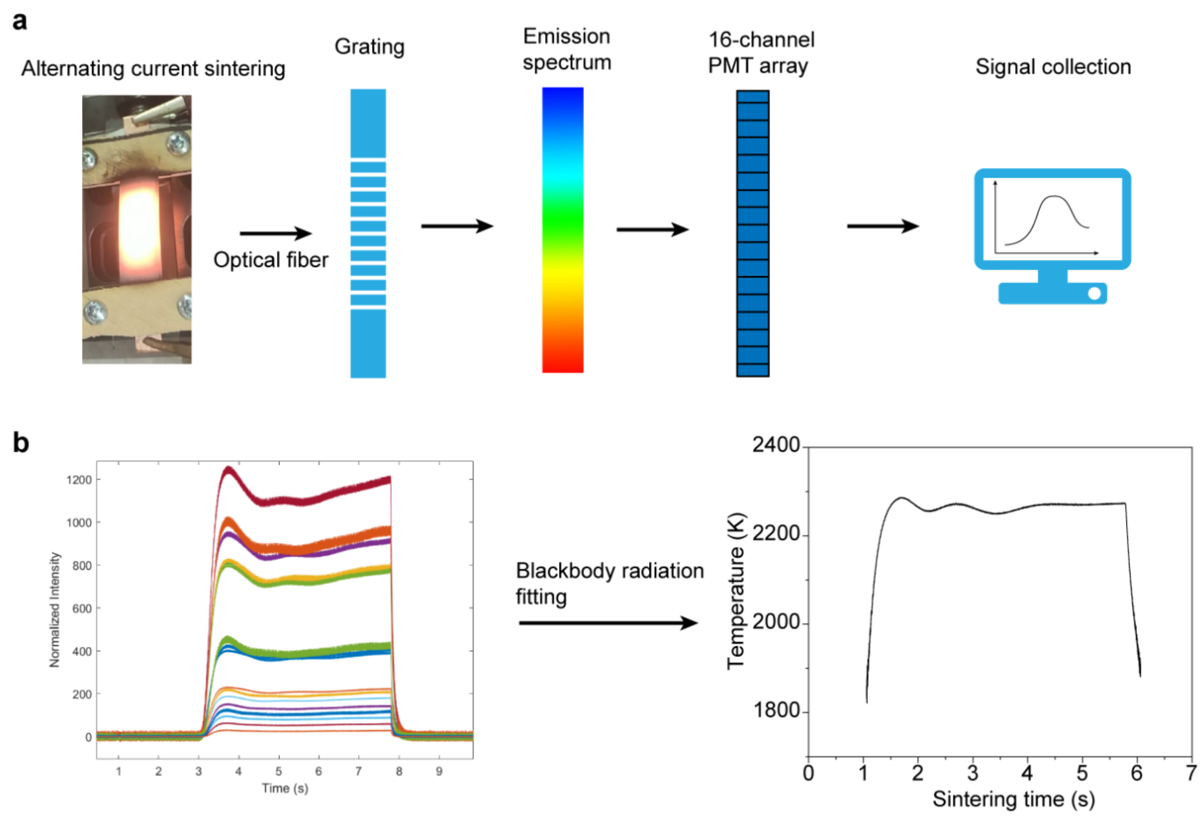

Supplementary Fig. 30. Time-resolved temperature measurement. (**a**) Schematic of the time-resolved spectrometer for emission spectrum collection during ultrafast Joule heating sinter. The emission light was collected by an optical fiber and separated by a customized grating black box. The spectrum radiance was recorded using a 16-channel photodiode array (Hamamatsu S4100-16R) at 600–1000 nm. The bias voltage signals from the photodiode arrays were collected by a National Instrument multifunction I/O device PCIe-6320. (**b**) Temperature determination by fitting the blackbody radiation. The spectra fitting was conducted using MATLAB. The script is little revised from the reference[10]. Prior to measurement, the temperature was calibrated using a 2800 K lamp.

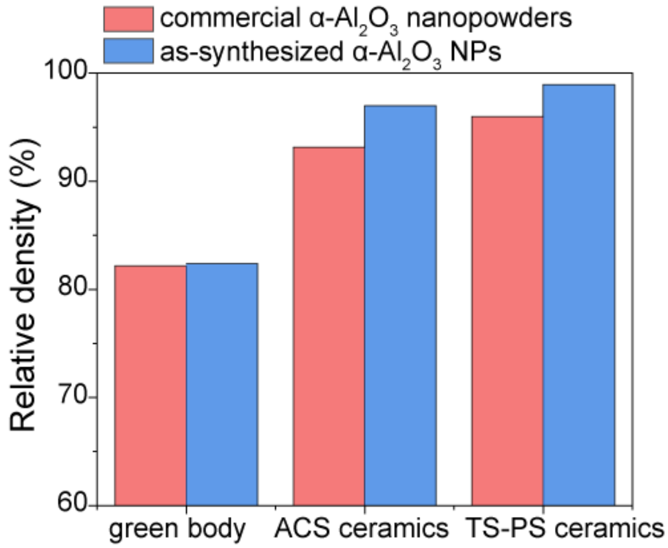

Supplementary Fig. 31. Density of the green body and alumina ceramics by alternating current sintering (ACS) and two-step pressureless sintering (TS-PS). The densities and relative densities values are also shown in Supplementary Table 10.

Discussion:

(1) The green bodies from different precursors have almost the same densities, demonstrating that the hydraulic press process is similar for two precursors under the same conditions.

(2) For both the newly developed ACS process and the TS-PS process, the density of the ceramics from the as-synthesized α-Al_2O_3 NPs is higher than that from the commercial α-Al_2O_3 nanopowders, indicating that the fine nanoparticle precursors facilitate the densification process.

(3) The density of the ceramic sintered by TS-PS process is somewhat higher than that by ACS process. Nevertheless, it is already promising that the 1 min ACS process could achieve a density of ~97% for the as-synthesized α-Al_2O_3 NPs, once again demonstrating that the fine nanoparticles as precursors is beneficial for the fast deification.

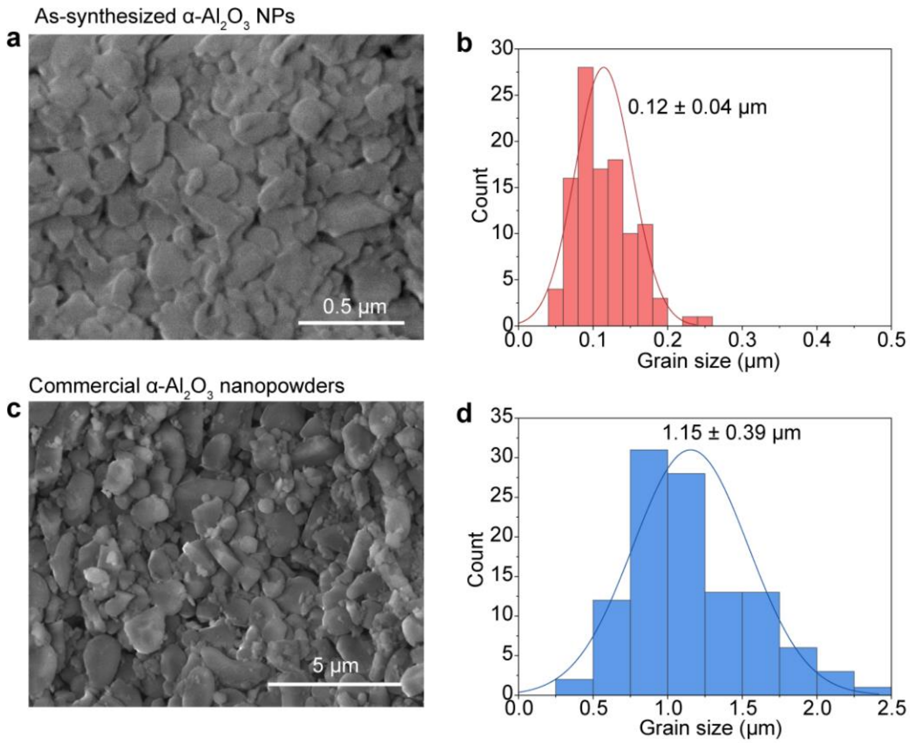

Supplementary Fig. 32. Microstructure and grain size distribution of the ceramics by alternating current sintering (ACS). (**a,b**) Representative scanning electron microscopy (SEM) image (**a**) of the sintered ceramics using as-synthesized α-Al_2O_3 NPs as precursor, and corresponding grain size distribution (**b**). (**c,d**) Representative SEM image (**c**) of the sintered ceramics using commercial α-Al_2O_3 nanopowders as precursor, and corresponding grain size distribution (**d**).

Discussion: By using the ACS process, the ceramics from the as-synthesized α-Al_2O_3 NPs precursor has a fine average grain size (~0.12 μm), much smaller than that from the commercial α-Al_2O_3 nanopowders (~1.15 μm).

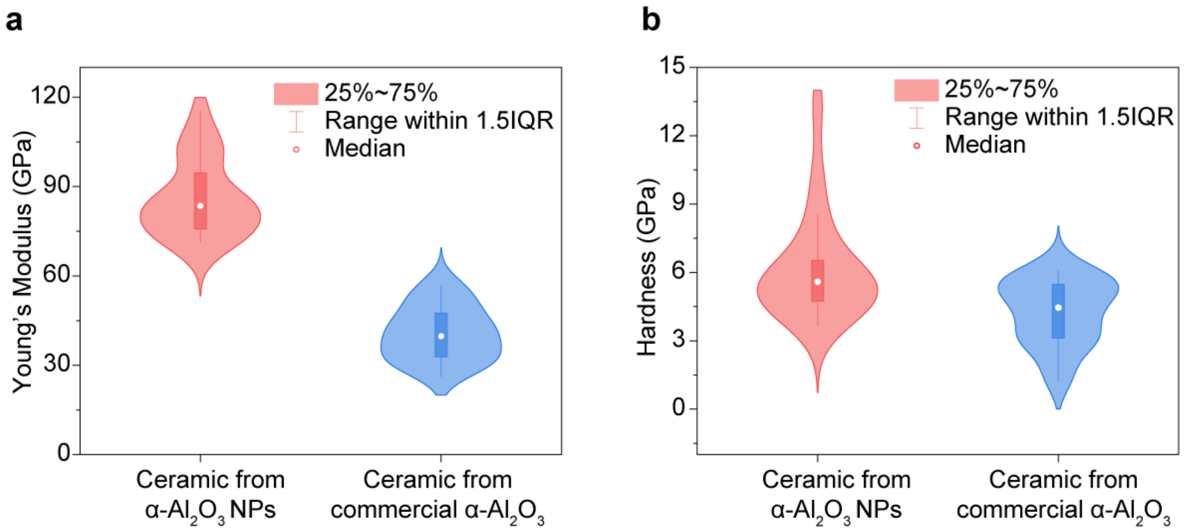

Supplementary Fig. 33. Mechanical properties measurement of the ceramics sintered by alternating current sintering (ACS) process. (**a**) Young's moduli distribution of the alumina ceramics using the α-Al_2O_3 NPs (red) and commercial α-Al_2O_3 nanopowders (blue) as precursors. The dot within the box indicates the median, and the range indicates the 1.5IQR. (**b**) Hardness distribution of the alumina ceramics using the α-Al_2O_3 NPs (red) and commercial α-Al_2O_3 nanopowders (blue) as precursors. The dot within the box indicates the median, and the range indicates the 1.5IQR.

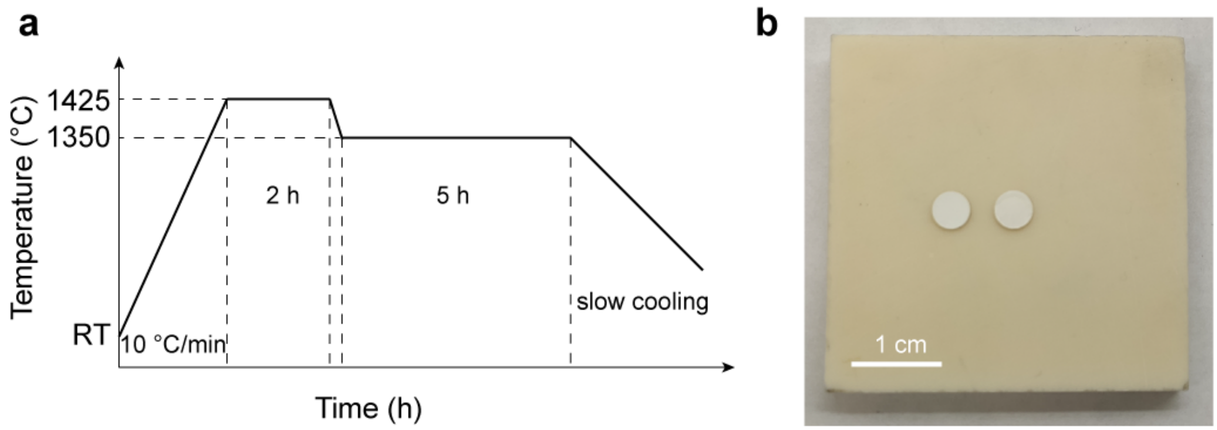

Supplementary Fig. 34. Ceramics sintering by two-step pressureless sintering (TS-PS) using a high-temperature furnace. (**a**) The temperature profile used for the sintering. (**b**) The picture of the sintered ceramics by using the as-synthesized α-Al_2O_3 NPs as precursor (left) and the commercial α-Al_2O_3 nanopowders as the precursor (right).

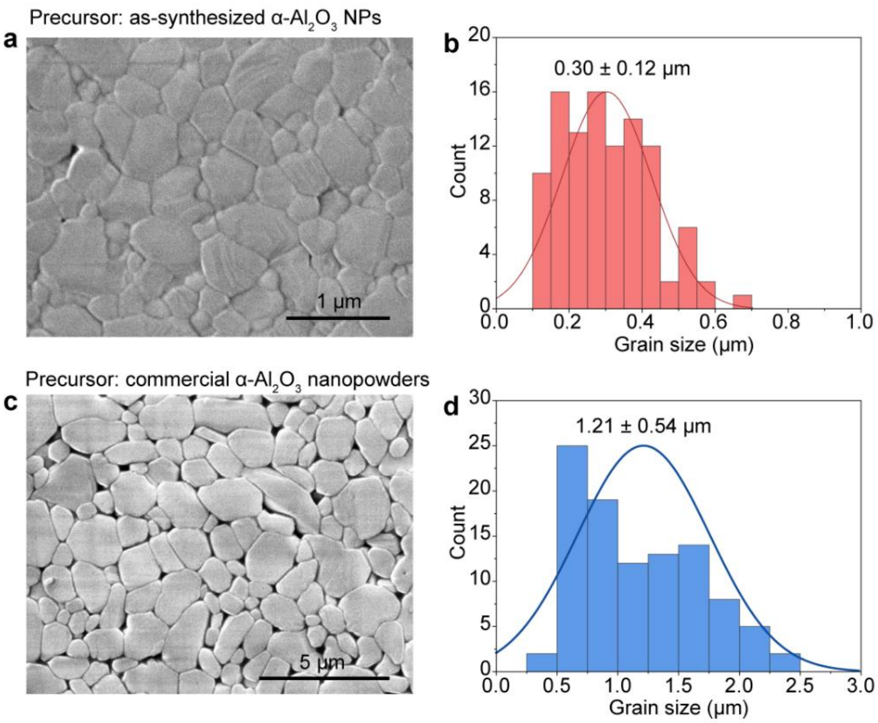

Supplementary Fig. 35. Microstructure and grain size distribution of the ceramics by two-step pressureless sintering (TS-PS) process. (**a,b**) Representative scanning electron microscopy (SEM) image (**a**) of the sintered ceramics using as-synthesized α-Al_2O_3 NPs as precursor, and corresponding grain size distribution (**b**). (**c,d**) Representative SEM image (**c**) of the sintered ceramics using commercial α-Al_2O_3 nanopowders as precursor, and corresponding grain size distribution (**d**).

Discussion: The nanoparticle precursors help to obtain the nano-grained ceramics with average grain size of ~0.3 μm, much smaller than the commercial ones (~1.21 μm). In addition, the SEM images show that the ceramic sintered from the as-synthesized α-Al_2O_3 NPs is fully sintered, while the ceramic from the commercial α-Al_2O_3 nanopowders is still with high porosity even after long-time sintering. This indicates that the nanoparticle precursors facilitate the grain growth and densification process.

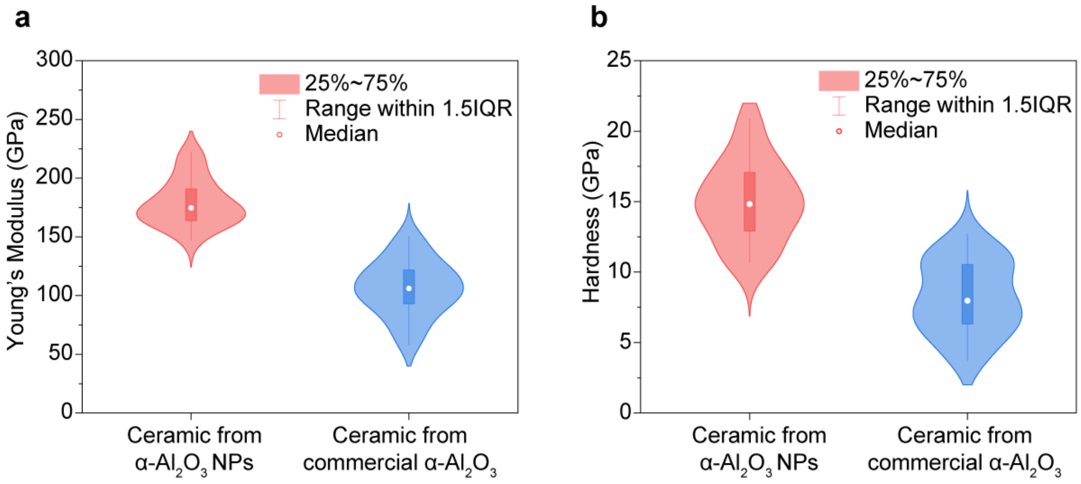

Supplementary Fig. 36. Mechanical properties measurement of the ceramics sintered by two-step presureless sintering (TS-PS) process. (**a**) Young's moduli distribution of the alumina ceramics by TS-PS process using the α-Al_2O_3 NPs (red) and commercial α-Al_2O_3 nanopowders (blue) as precursors. The dot within the box indicates the median, and the range indicates the 1.5IQR. (**b**) Hardness distribution of the alumina ceramics by alternating current sintering (ACS) process using the α-Al_2O_3 NPs (red) and commercial α-Al_2O_3 nanopowders (blue) as precursors. The dot within the box indicates the median, and the range indicates the 1.5IQR.

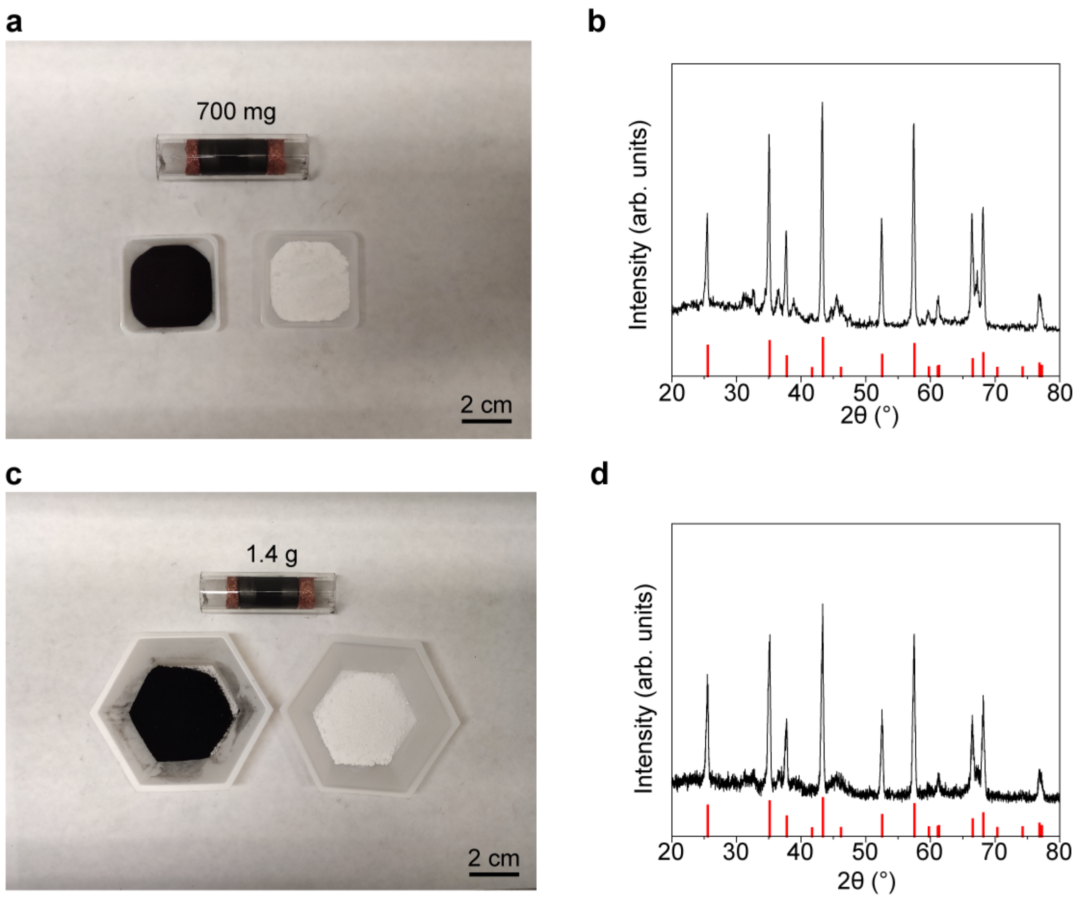

Supplementary Fig. 37. Scalability of the pulsed direct current (PDC) process. (**a**) Picture of the sample with mass of 700 mg synthesized using a tube (D = 15 mm) and PDC voltage of 60 V. (**b**) The X-ray diffraction (XRD) pattern of the product. The reference PDF card for α-Al_2O_3: No. 46-1212. (**c**) Picture of the sample with mass of 1.4 g synthesized using a tube (D = 15 mm) and PDC voltage of 120 V. (**d**) The XRD pattern of the product. The reference PDF card for α-Al_2O_3: No. 46-1212. The black powders in **a** and **c** are as-synthesized mixture of carbon black (CB) and α-Al_2O_3, and the white powders are α-Al_2O_3 after calcination.

Supplementary Table 1. Parameters for PDC Joule heating.

Precursors	Mass Ratio	Initial Mass (mg)	***R*** **(Ω)**	V_0 **(V)**	V_1 **(V)**	Duration (ms)	Final Mass (mg)
γ-Al_2O_3:CB	1:1	150	1.5	60	9	800	132 mg
γ-Al_2O_3:CB	2:1	150	2	60	35	800	128 mg
γ-Al_2O_3:CB	3:1	150	4	60	38	800	140 mg
γ-Al_2O_3:CB	4:1	150	8	60	42	800	135 mg
γ-Al_2O_3:CB	5:1	150	20	60	50	800	146 mg
γ-Al_2O_3:CB	4:1	150	8	60	55	300	132 mg
γ-Al_2O_3:CB	4:1	150	8	60	52	400	135 mg
γ-Al_2O_3:CB	4:1	150	8	60	40	500	130 mg
γ-Al_2O_3:CB	4:1	150	8	60	42	800	135 mg
CB	--	150	0.8	60	12	500	122 mg

Note: V_0: the start voltage of the capacitor bank, V_1: the voltage of the capacitor bank after PDC Joule heating.

Supplementary Table 2. Volume fraction of γ-Al_2O_3.

Mass ratio	Mass ratio of CB	*f*(γ-Al_2O_3)
γ-Al_2O_3:CB = 1:1	0.50	0.42
γ-Al_2O_3:CB = 2:1	0.33	0.59
γ-Al_2O_3:CB = 3:1	0.25	0.67
γ-Al_2O_3:CB = 4:1	0.20	0.73
γ-Al_2O_3:CB = 5:1	0.17	0.78

Supplementary Table 3. Geometry parameters for simulation.

f(γ-Al_2O_3)	Size	Diameter of γ-Al_2O_3	Number of γ-Al_2O_3
0.42	110 nm × 110 nm	20 nm	16
0.59	92 nm × 92 nm	20 nm	16
0.67	108 nm × 108 nm	20 nm	25
0.73	104 nm × 104 nm	20 nm	25
0.78	100 nm × 100 nm	20 nm	25

Supplementary Table 4. Materials parameters for simulation.

Materials	Carbon black	γ-Al_2O_3
Relative permittivity	30	10
Electrical conductivity	124 S m^{-1}	1.5×10^{-7} S m^{-1}

Supplementary Table 5. Boundary conditions for simulation.

f(γ-Al_2O_3)	Electrical Potential (V)	Ground (V)
0.42	1.3×10^{-3}	0
0.59	1.1×10^{-3}	0
0.67	1.3×10^{-3}	0
0.73	1.2×10^{-3}	0
0.78	1.2×10^{-3}	0

Supplementary Table 6. Calculation results summary.

**Phase**	**γ-Al_2O_3**		**δ'-Al_2O_3**		**α-Al_2O_3**	
Lattice constants (Å)	$a = b = 8.003$<br>$c = 24.220$		$a = b = 8.041$<br>$c = 23.664$		$a = b = 4.807$<br>$c = 13.117$	
Density (Al_2O_3/Å^3)	0.02064		0.02092		0.02285	
Cohesive energy: μ (eV/Al_2O_3)	-37.08		-37.35		-37.40	
Surface energy without surface OH: ϵ_i (eV/Å^2)	(100)	0.0382	(100)	0.0786	(0001)	0.0983
	(110)	0.1210	(110)	0.1229	$(1\bar{1}00)$	0.1271
	(111)	0.0826	(111)	0.0847	$(11\bar{2}0)$	0.1209
Surface energy with 2 OH/nm^2: ϵ_i (eV/Å^2)	(100)	0.0231	(100)	0.0597	(0001)	0.0715
	(110)	0.0611	(110)	0.0845	$(1\bar{1}00)$	0.0943
	(111)	0.0320	(111)	0.0596	$(11\bar{2}0)$	0.0887
Optimized nanoparticle shapes	Shape: Truncated cube<br>Square facets: (100)<br>Triangular facets: (111)		Shape: Truncated octahedron<br>Square facets: (100)<br>Hexagonal facets: (111)		Shape: Dodecagonal Prism<br>Bottom facets: (0001)<br>Side facets: $(11\bar{2}0)$, $(1\bar{1}00)$	

Supplementary Table 7. Synthesis of α-Al_2O_3 by thermal process.

Method	Particle size (nm)	Surface area (m^2 g^{-1})	Temperature (K)	Reference
Flame spray pyrolysis	29–88	40–60	1873	Laine *et. al*, 2006[11]
Furnace calcination	~30	~50	1473	Johnston *et. al*, 1992[12]
Precipitation/calcination	100	---	1173	Li *et. al*, 2000[13]
Furnace calcination	150	---	1273	Zhang et al., 2008[14]
Furnace calcination	35	---	1473	Yoo et al., 2009[15]
PDC Joule heating	~23	~65	573	This work

Supplementary Table 8. Lattice symmetry and the coordination number of O and Al atoms defined by the first neighbors.

	α (Hexagonal)	γ (Cubic)	
Al	6	6 (3/4 atoms)	4(1/4 atoms)
O	4	4 (2/3 atoms)	3(1/3 atoms)

Supplementary Table 9. Evolution of average coordination number of Al and O during the MD simulation of the γ-Al_2O_3 structure transformation at 2000 K.

t (ps)	0	5	10	15	20	25	30
Al	5.50	5.67	5.70	5.75	5.78	5.73	5.80
O	3.67	3.79	3.80	3.83	3.96	3.92	3.88

Supplementary Table 10. Density measurement.

Precursors	Samples	Density (g cm^{-3})	Relative density
Commercial α-Al_2O_3 NPs	Green body	3.28	82%
	ACS sintering	3.72	93%
	TS-PS sintering	3.83	96%
As-synthesized α-Al_2O_3 NPs	Green body	3.29	82%
	ACS sintering	3.87	97%
	TS-PS sintering	3.95	99%

Note:
ACS condition: 10 V, 1 min;
TS-PS condition: RT to 1425 °C at 10 °C/min; 1425 °C for 2 h; 1350 °C for 5 h.

Supplementary Table 11. Sintering of the alumina ceramics from alumina nanoparticles.

**Sintering method**	**Starting materials**	**Sintering conditions**	**Relative Density**	**Grain size**	**Mechanics**	**Reference**
TS-PS	α-Al_2O_3, 30–40 nm	Step 1: 1425 °C 2 h Step 2: 1350 °C 5 h	≥99.5%	≤500 nm	No report	*Nat. Mater*. **5**, 710-712 (2006)[11]
TS-PS	α-Al_2O_3, 10–80 nm	Step 1: 1450 °C 1 h Step 2: 1350 °C 50 h	95%	70 nm	No report	*J. Am. Ceram. Soc.* **89**, 139 (2006)[16]
TS-PS	α-Al_2O_3, 2–27 nm	Step 1: 1175 °C Step 2: 1025 °C 20 h	99.6%	12-104 nm	No report	*J. Am. Ceram. Soc.* **99**, 3556 (2016)[17]
PS	α-Al_2O_3, 200 nm	1450 °C, 2 h	99.2%	1.25 µm	Hardness: ~17 GPa	*J. Am. Ceram. Soc.* **78**, 1118-1120 (1995)[18]

PS	α-Al_2O_3, 200 nm	1300–1450 °C (depending on the shaping method), 2 h	92–100%	0.32–0.63 μm	Hardness: 15–20 GPa	*J. Eur. Ceram. Soc.* **16**, 1189-1200 (1996)[19]; *Mater. Sci. Eng. A* **307**, 172-181 (2001)[20]
PS	α-Al_2O_3, 40 nm	Step 1: 800 °C 2 h Step 2: 1200 °C 2 h	97%	0.25 μm	Hardness: ~15 GPa	*J. Am. Ceram. Soc.* **86**, 546-553 (2003)[21]
PS	α-Al_2O_3, 150–200 nm	1425 °C 2 h	98.3%	1.49 μm	No report	*J. Am. Ceram. Soc.* **86**, 1985-1992 (2006)[22]
RVS	α-Al_2O_3, 350 nm MgO (30 nm, 0.25 wt%)	Pre-heating: 1200 °C 2 h Sintering: 1650 °C 5 min	99.5%	4 μm	Flexural strength: 570 MPa	*Ceram. Int.* **41**, 12499-12503 (2015)[23]
TS-PS	Commercial α-Al_2O_3, ~300 nm	Step 1: 1425 °C 2 h Step 2: 1350 °C 5 h	96%	1.21 μm	Modulus: 106 ± 24 GPa Hardness: 8.2 ± 2.6 GPa	This work
TS-PS	Synthesized α-Al_2O_3, ~23 nm	Step 1: 1425 °C 2 h Step 2: 1350 °C 5 h	99%	0.30 μm	Modulus: 179 ± 20 GPa Hardness: 15.0 ± 2.8 GPa	This work
ACS	Commercial α-Al_2O_3, ~300 nm	Voltage input: ~12 V Time: 1 min	93%	1.15 μm	Modulus: 40.6 ± 9.1 GPa Hardness: 4.2 ± 1.4 GPa	This work
ACS	Synthesized α-Al_2O_3,	Voltage input: ~12 V	97%	0.12 μm	Modulus: 86.0 ± 12.8 GPa	This work

	~23 nm	Time: 1 min			Hardness: 6.2 ± 2.3 GPa	
Commercial alumina AD-99			99%	15–20 μm	Hardness: 12 GPa	*J. Am. Ceram. Soc.* **86**, 546-553 (2003)[21]
Commercial Standard Alumina			95%	7–13 μm	Hardness: 10.5–12.7 GPa	Ref[24]
Sapphire			100%	Single crystal	Hardness: 15–17 GPa	*Mater. Sci. Eng. A* **307**, 172-181 (2001)[20]

Note: Pressureless sintering (PS), Two-step pressureless sintering (TS-PS), Rapid vacuum sintering (RAS), Alternating current sintering (ACS).

Supplementary References

1 Halder, N. C. & Wagner, C. N. J. Separation of particle size and lattice strain in integral breadth measurements. *Acta. Crystallogr.* **20**, 312-313 (1966).

2 Steiner, C. J. P., Hasselman, D. P. H., Sprigges & R. M. Kinetics of the gamma-to-alpha alumina phase transformation. *J. Am. Ceram. Soc.* **54**, 412-413 (1971).

3 Zhao, Y. F. & Yakobson, B. I. What is the ground-state structure of the thinnest Si nanowires? *Phys. Rev. Lett.* **91**, 035501-035504 (2003).

4 Amrute, A. P., Lodziana, Z., Schreyer, H., Weidenthaler, C. & Schuth, F. High-surface-area corundum by mechanochemically induced phase transformation of boehmite. *Science* **366**, 485-489 (2019).

5 National Institute of Standards and Technology, U.S. Department of Commerce, NIST-JAAF Thermochemical Tables (1998).

6 Parrinello, M. & Rahman, A. Crystal structure and pair potentials: A molecular dynamics study. *Phys. Rev. Lett.* 4**5,** 1196-1199 (1980).

7 Parrinello, M. & Rahman, A. Polymorphic transitions in single crystals: A new molecular dynamics method. *J. Appl. Phys.* **52,** 7182-7190 (1981).

8 Luong, D. X. et al. Gram-scale bottom-up flash graphene synthesis. *Nature* **577**, 647-651 (2020).

9 Levin, I. & Brandon, D. Metastable alumina polymorphs: Crystal structures and transition sequences. *J. Am. Ceram. Soc.* **81**, 1995-2012 (1998).

10 Sathyanarayan Rao (2021). Blackbody Radiation Spectrum from Wien's Law and Planck's law (https://www.mathworks.com/matlabcentral/fileexchange/48253-blackbody-radiation-spectrum-from-wien-s-law-and-planck-s-law), MATLAB Central File Exchange. Retrieved March 14, 2021.

11 Laine, R. M., Marchal, J. C., Sun, H. P. & Pan, X. Q. Nano-alpha-Al_2O_3 by liquid-feed flame spray pyrolysis. *Nat. Mater.* **5**, 710-712 (2006).

12 Johnston, G. P., Muenchausen, R., Smith, D. M., Fahrenholtz, W. & Foltyn, S. Reactive laser ablation synthesis of nanosize alumina powder. *J. Am. Ceram. Soc.* **75**, 3293-3298 (1992).

13 Li, J. G. & Sun, X. D. Synthesis and sintering behavior of a nanocrystalline alpha-alumina powder. *Acta. Mater.* **48**, 3103-3112 (2000).

14 Zhang, X. X., Ge, Y. L., Hannula, S. P., Levanen, E. & Mantyla, T. Nanocrystalline alpha-alumina with novel morphology at 1000 °C. *J. Mater. Chem.* **18**, 2423-2425 (2008).

15 Yoo, Y. S., Park, K. Y., Jung, K. Y. & Cho, S. B. Preparation of alpha-alumina nanoparticles via vapor-phase hydrolysis of $AlCl_3$. *Mater. Lett.* **63**, 1844-1846 (2009).

16 Li, J. & Ye, Y. Densification and grain growth of Al_2O_3 nanoceramics during pressureless sintering. *J. Am. Ceram. Soc.* **89**, 139-143 (2006).

17 Guo, R. Y., Cao, W. B., Mao, X. & Li, J. G. Selective corrosion preparation and sintering of disperse α-Al_2O_3 nanoparticles. *J. Am. Ceram. Soc.* **99**, 3556-3560 (2016).

18 Krell, A. & Blank, P. Grain size dependence of hardness in dense submicrometer alumina. *J. Am. Ceram. Soc.* **78**, 1118-1120 (1995).

19 Krell, A. & Blank, P. The Influence of shaping method on the grain size dependence of strength in dense submicrometre alumina. *J. Eur. Ceram. Soc.* **16**, 1189-1200 (1996).

20 Krell, A. & Schädlich, S. Nanoindentation hardness of submicrometer alumina ceramics. *Mater. Sci. Eng. A* **307**, 172-181 (2001).

21 Krell, A., Blank, P., Ma, H., Hutzler, T. & Nebelung, M. Processing of high-density submicrometer Al_2O_3 for new applications. *J. Am. Ceram. Soc.* **86**, 546-553 (2003).

22 Krell, A. & Klimke, J. Effects of the homogeneity of particle coordination on solid-state sintering of transparent alumina. *J. Am. Ceram. Soc.* **89**, 1985-1992 (2006).

23 Zhang, H. et al. Effects of heating rate on the microstructure and mechanical properties of rapid vacuum sintered translucent alumina. *Ceram. Int.* **41,** 12499-12503 (2015).

24 https://www.ceramics.net/ceramic-materials-solutions/aluminas/std-alumina, accessed 8th May, 2022.
